# Suppressor of IKKɛ is an essential negative regulator of pathological cardiac hypertrophy

**DOI:** 10.1038/ncomms11432

**Published:** 2016-06-01

**Authors:** Ke-Qiong Deng, Aibing Wang, Yan-Xiao Ji, Xiao-Jing Zhang, Jing Fang, Yan Zhang, Peng Zhang, Xi Jiang, Lu Gao, Xue-Yong Zhu, Yichao Zhao, Lingchen Gao, Qinglin Yang, Xue-Hai Zhu, Xiang Wei, Jun Pu, Hongliang Li

**Affiliations:** 1 Department of Cardiology, Renmin Hospital of Wuhan University, Wuhan 430060, China; 2 Animal Experiment Center/Animal Biosafety Level-III Laboratory, Wuhan University, Wuhan 430060, China; 3 Medical Research Institute, School of Medicine, Wuhan University, Wuhan 430071, China; 4 College of Veterinary Medicine, Hunan Agricultural University, Changsha 410128, China; 5 Division of Cardiothoracic and Vascular Surgery, Heart-Lung Transplantation Center, Sino-Swiss Heart-Lung Transplantation Institute, Tongji Hospital, Tongji Medical College, Huazhong University of Science and Technology, Wuhan 430000, China; 6 Department of Cardiology, Institute of Cardiovascular Disease, Union Hospital, Tongji Medical College, Huazhong University of Science and Technology, Wuhan 430000, China; 7 Department of Cardiology, Shanghai Renji Hospital, School of Medicine, Shanghai Jiaotong University, Shanghai 200127, China; 8 Department of Nutrition Sciences, University of Alabama at Birmingham, Birmingham, Alabama 35294-3360, USA

## Abstract

Although pathological cardiac hypertrophy represents a leading cause of morbidity and mortality worldwide, our understanding of the molecular mechanisms underlying this disease is still poor. Here, we demonstrate that suppressor of IKKɛ (SIKE), a negative regulator of the interferon pathway, attenuates pathological cardiac hypertrophy in rodents and non-human primates in a TANK-binding kinase 1 (TBK1)/AKT-dependent manner. *Sike*-deficient mice develop cardiac hypertrophy and heart failure, whereas Sike-overexpressing transgenic (*Sike*-TG) mice are protected from hypertrophic stimuli. Mechanistically, SIKE directly interacts with TBK1 to inhibit the TBK1-AKT signalling pathway, thereby achieving its anti-hypertrophic action. The suppression of cardiac remodelling by SIKE is further validated in rats and monkeys. Collectively, these findings identify SIKE as a negative regulator of cardiac remodelling in multiple animal species due to its inhibitory regulation of the TBK1/AKT axis, suggesting that SIKE may represent a therapeutic target for the treatment of cardiac hypertrophy and heart failure.

## Abstract (web summary)

Identifying pathways that cause pathological cardiac hypertrophy holds great therapeutic potential. Here the authors discover one such pathway and show that SIKE, an inhibitor of interferon signalling, prevents pathological but not physiological cardiac hypertrophy by interacting with TBK1 and modulating the TBK1/AKT signalling in rodents and monkeys.

## Graphical abstract

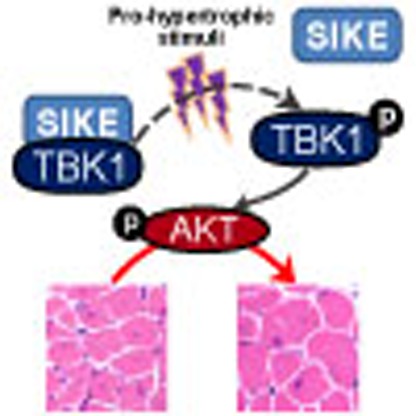

Pathological cardiac hypertrophy is characterized by an increase in cardiomyocyte size, increased protein synthesis, re-expression of fetal genes and a shift from fatty acids to glucose as an energy source^1^,^2^,^3^. Although cardiac hypertrophy initially might be compensatory and adaptive, prolonged pathological hypertrophy is deleterious and can lead to decompensation, diastolic dysfunction and ultimately heart failure and sudden death^4^. Thus, cardiac hypertrophy represents a leading cause of morbidity and mortality worldwide^5^,^6^,^7^,^8^. Accumulating evidence suggests that the pathological hypertrophy response is coordinated by an orchestrated hypertrophic network that comprises numerous signalling pathways, including β-adrenergic receptor-cAMP-PKA, GPCR-Gaq/PLC-PKCα, phosphatidylinositol 3-kinase (PI3K)/AKT, Ca^2+^/calmodulin-dependent kinase II and small G protein-mitogen-activated protein kinase (MAPK) signalling cascades^1^,^9^,^10^. However, much less is known about how cardiac hypertrophy is suppressed. These negative regulators could be of significant therapeutic value and could facilitate the development of gene-based therapies for the treatment of cardiac hypertrophic pathologies^11^,^12^.

Suppressor of IKKɛ (SIKE) is a small coiled–coil domain-containing protein comprised of 207 amino-acid residues, and is ubiquitously expressed in most tissues, including the heart. Since being initially identified as an interaction partner for IKKɛ and a negative regulator of the interferon pathway by negatively modulating TANK-binding kinase 1 (TBK1)-involved signalling^13^, SIKE has not been further studied for its additional biological activities. In the current study, we demonstrate that SIKE expression is suppressed in pathological hypertrophic hearts. Employing cardiac-specific *Sike* knockout and transgenic (TG) mice, we demonstrate that Sike suppress pathological cardiac remodelling elicited by pressure overload or angiotensin II (Ang II) challenge. The protective effects of SIKE on cardiac hypertrophy are confirmed in rats and monkeys. In addition, our mechanistic investigations emphasize the role of TBK1-AKT cascades during SIKE-regulated cardiac hypertrophy.

## Results

### SIKE expression is downregulated in hypertrophic hearts

**Figure 1:**
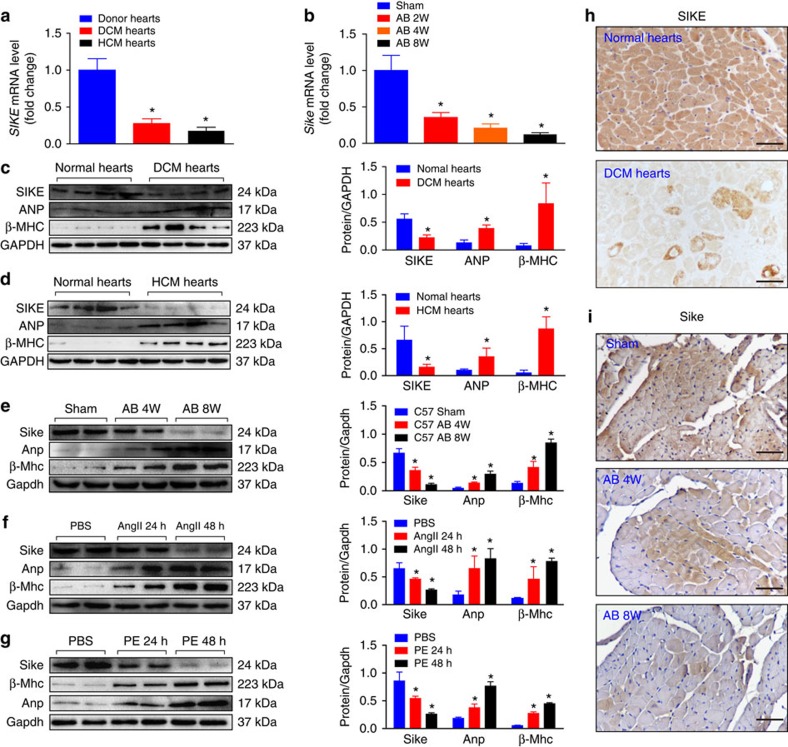
SIKE expression is downregulated in hypertrophic hearts. (**a**) *SIKE* mRNA levels in normal, DCM and HCM human hearts. (**b**) *Sike* mRNA levels in the hearts of sham-operated controls and AB-induced cardiac hypertrophic mice at the indicated times. Values represent the fold changes relative to the sham controls, set as 1; the *GAPDH* mRNA level was used as the internal control in **a** and **b**. (**c**–**g**) Immunoblot and quantification of SIKE, ANP and β-MHC protein levels in normal and DCM human hearts (**c**), normal and HCM human hearts (**d**), control and 4- to 8-week AB-induced hypertrophic mouse hearts (**e**), and 1 μM Ang II-treated (**f**) or 100 μM PE-treated (**g**) neonatal rat cardiomyocytes (NRCMs) for the indicated times; *n*=4 samples or repeats/group; the GAPDH protein level was used for normalization. (**h**,**i**) Representative images of immunohistochemical staining of normal or DCM human heart sections (**h**) and control or hypertrophic mouse heart sections (**i**) with an antibody against SIKE; scale bars, 50 μm. *P<0.05 versus the corresponding controls (normal hearts, sham-operated mouse hearts or PBS-treated cardiomyocytes in **a**–**g**). Data are presented as the mean±s.d. from at least three independent experiments. For **c** and **d**, statistical analysis was carried out by Student's two-tailed *t*-test; for **a**,**b** and **e**–**g** statistical analysis was carried out by one-way analysis of variance.

To explore the potential roles of SIKE in cardiac hypertrophy, we first investigated whether the *SIKE* expression level was altered under cardiac pathological conditions. The *SIKE* mRNA level was significantly reduced in human hearts with dilated cardiomyopathy (DCM) and hypertrophic cardiomyopathy (HCM) compared with normal hearts (Fig. 1a), as well as in aortic banding (AB)-challenged hypertrophic mouse hearts compared with sham-operated hearts (Fig. 1b). Consistently, human and mouse hypertrophic hearts showed significantly decreased SIKE protein with remarkably increased hypertrophic marker proteins, that is, atrial natriuretic peptide (ANP) and β-myosin heavy chain (β-MHC; Fig. 1c–e). This decrease in the SIKE level in hypertrophic cardiac cells was confirmed in cultured neonatal rat cardiomyocytes (NRCMs) stimulated with either Ang II (1 μM) or phenylephrine (PE; 100 μM) for 48 h to induce hypertrophy (as evidenced by increased Anp and β-Mhc levels; Fig. 1f,g). Furthermore, immunohistochemical staining of human DCM heart and mouse hypertrophic heart sections also demonstrated reduced SIKE expression in cardiomyocytes, compared with their corresponding controls (Fig. 1h,i). Collectively, these findings suggested a potential role of SIKE in pathological heart hypertrophy.

### SIKE deficiency in the heart promotes AB-induced hypertrophy

**Figure 2:**
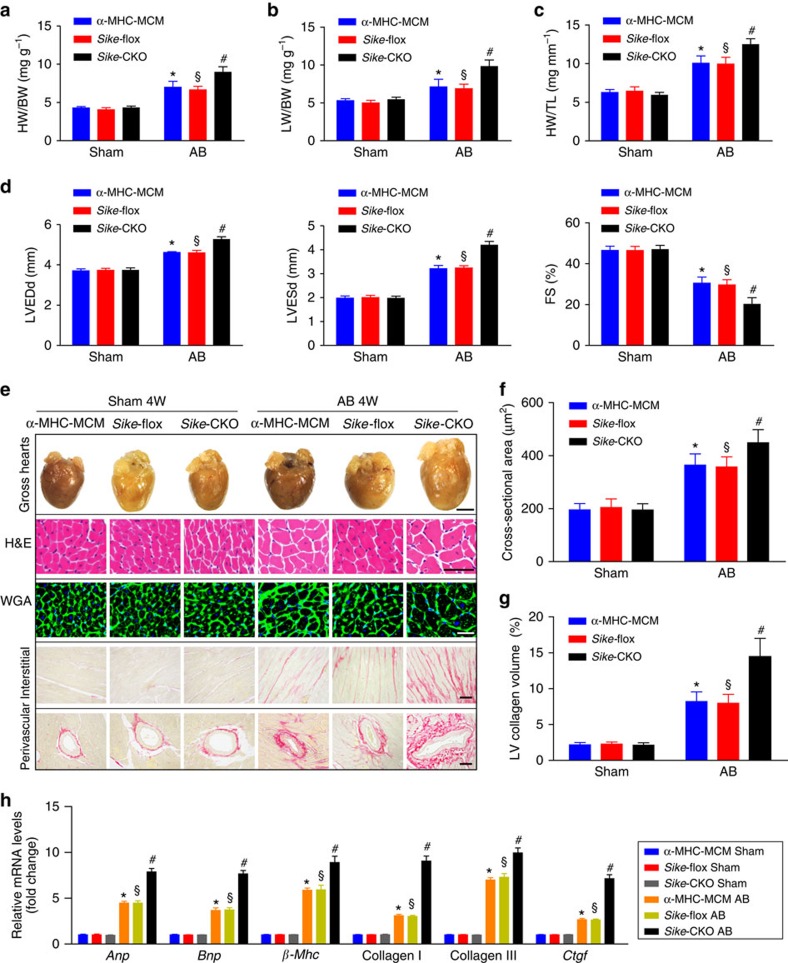
Sike deficiency in the heart promotes pressure overload-induced hypertrophy. (**a**–**c**) Comparison of the HW/BW (**a**), LW/BW (**b**) and HW/TL (**c**) ratios in different genotypic mice (α-MHC-MCM, *Sike*-flox and *Sike*-CKO) after sham or AB surgery, *n*=12–15 mice/group. (**d**) Comparison of echocardiographic parameters (LVEDd, LVESd and FS) in the indicated groups, *n*=10–12 mice per group. (**e**) Histological analyses of whole hearts (the first row; scale bar, 2,000 μm) and heart sections stained with H&E (the second row; scale bar, 50 μm), WGA (the third row; scale bar, 20 μm) or PSR (the fourth and fifth row; scale bars, 50 μm) from the indicated groups 4 weeks after sham or AB surgery, *n*=6–8 mice per group. (**f**) Comparison of the average cross-sectional area of cardiomyocytes from the indicated groups, *n*≥100 cells per group. (**g**) Comparison of the LV collagen volume in the indicated groups, *n*≥40 fields per group. (**h**) mRNA levels of the hypertrophic marker genes (*Anp*, *Bnp* and *β-Mhc*) and fibrotic marker genes (collagen I, collagen II and *Ctgf*) in the indicated groups, *n*=4 independent experiments. *P<0.05 versus the sham-operated α-MHC-MCM group; ^§^*P*<0.05 versus the sham-operated *Sike*-flox group; ^#^*P*<0.05 versus the AB-operated α-MHC-MCM or *Sike*-flox group in **a**–**d**, **f**–**h**. Data are presented as the mean±s.d. from at least three independent experiments. Statistical analysis was carried out by one-way analysis of variance.

As SIKE expression was suppressed in hypertrophic hearts, we investigated whether the reduced SIKE level affected cardiac remodelling and therefore generated cardiac-specific *Sike*-knockout (*Sike*-CKO) mice (Supplementary Fig. 1a). The tissue-specific loss of Sike protein in the hearts from these mice was confirmed by western blotting (Supplementary fig. 1b). We subsequently performed sham or AB surgery on *Sike*-CKO mice. Age-matched α-MHC-MCM mice and *Sike*^*flox/flox*^ (*Sike*-flox) mice subjected to the same surgery in parallel served as controls. Notably, the *Sike*-CKO mice did not exhibit marked abnormalities in heart structure or function under basal conditions. However, the *Sike*-CKO mice showed significantly increased ratios of heart weight to body weight (HW/BW), lung weight to BW (LW/BW) and HW to tibia length (HW/TL) compared with the α-MHC-MCM or *Sike*-flox mice 4 weeks after AB surgery (Fig. 2a–c). Furthermore, the AB-operated *Sike*-CKO mice exhibited remarkably exacerbated cardiac dilation and dysfunction on the echocardiography assay, as compared with the control α-MHC-MCM or *Sike*-flox mice (Fig. 2d). Moreover, enlargement of the heart and cardiomyocytes was identified via haematoxylin and eosin (H&E) or wheat germ agglutinin (WGA)-stained heart sections and according to the cardiomyocyte cross-sectional area in the AB-operated *Sike*-CKO and control mice (Fig. 2e,f), whereas picrosirius red (PSR)-stained heart sections exhibited more prominent interstitial and perivascular fibrosis in *Sike*-CKO mice compared with the controls (Fig. 2e,g). The transcript levels of several hypertrophic marker genes (*Anp*, brain natriuretic peptide (*Bnp*) and *β-Mhc*) and fibrotic markers (collagen I, collagen III and connective tissue growth factor (*Ctgf*) were dramatically increased in the AB-operated *Sike*-CKO mice compared with the control mice 4 weeks after AB surgery (Fig. 2h). These data demonstrated that Sike ablation in the heart sensitized the mice to pressure overload challenge, which thereby promoted cardiac hypertrophy and fibrosis.

In addition to the pathological cardiac hypertrophy, we also examined the influence of Sike deficiency on the physiological hypertrophy induced by physical conditioning. After swimming training, a significant increase in the ratio of HW/BW and in the cardiac cross-section area were observed in the exercise group as compared with sedentary control animals without myocardial interstitial fibrosis (Supplementary Fig. 2a–c). However, interestingly, the hypertrophic response induced by physiological challenge failed to be regulated significantly by Sike deficiency (Supplementary Fig. 2a–c), and thus our subsequent studies focused on the role of SIKE in pathological cardiac remodelling.

### Cardiac Sike overexpression blunts AB-induced hypertrophy

**Figure 3:**
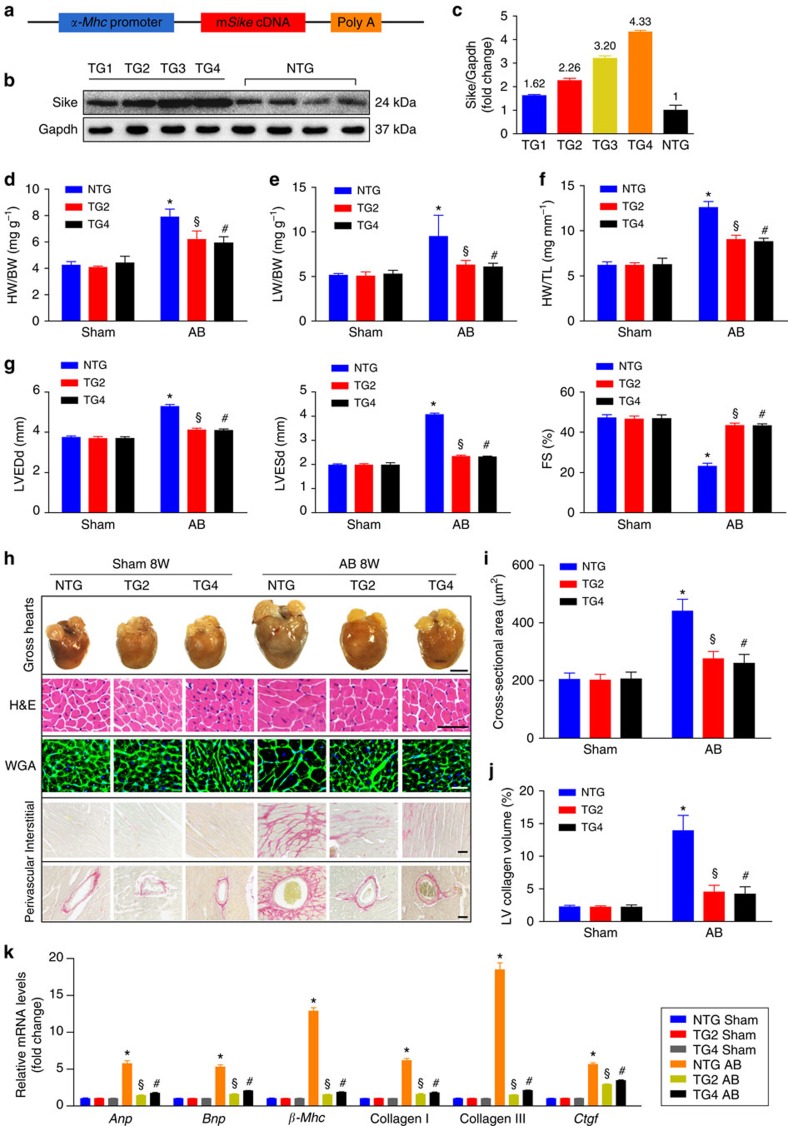
Cardiac Sike overexpression blunts the AB-induced hypertrophic response. (**a**) Schematic of the *α-Mhc* promoter-driven mouse cDNA of the *Sike* transgenic construct. (**b**,**c**) Immunoblot (**b**) and quantification (**c**) of Sike expression levels in the hearts of four different transgenic (TG) founders: TG1, TG2, TG3 and TG4. (**d**–**f**) Comparison of the HW/BW (**d**), LW/BW (**e**) and HW/TL (**f**) ratios in different genotypic mice (NTG, TG2 and TG4) that received sham or AB surgery, *n*=10–12 mice per group. (**g**) Comparison of the echocardiographic parameters in the indicated groups, *n*=8–10 mice per group. (**h**) Histological analyses of whole hearts (the first row; scale bar, 2,000 μm) and heart sections stained with H&E (the second row; scale bar, 50 μm), WGA (the third row; scale bar, 20 μm) or PSR (the fourth and fifth row; scale bars, 50 μm) from the indicated groups 8 weeks after sham or AB surgery, *n*=6–8 mice per group. (**i**) Comparison of the average cross-sectional area of cardiomyocytes from the indicated groups, *n*≥100 cells per group. (**j**) Comparison of the LV collagen volume in the indicated groups, *n*≥40 fields per group. (**k**) Transcript levels of hypertrophic marker genes (*Anp*, *Bnp* and *β-Mhc*) and fibrotic marker genes (collagen I, collagen II and *Ctgf*) in the indicated groups, *n*=4 independent experiments. *P<0.05 versus the sham-operated NTG group; ^§^*P*<0.05 versus the AB-operated NTG group; ^#^*P*<0.05 versus the AB-operated NTG group in **d**–**g**, **i**–**k**. Data are presented as the mean±s.d. from at least three independent experiments. Statistical analysis was carried out by one-way analysis of variance.

To further corroborate the role of SIKE in cardiac hypertrophy, we generated cardiac-specific *Sike*-TG mice (Fig. 3a). Several distinct mouse founders expressing different levels of increased Sike protein in the hearts were created (Fig. 3b,c), and we selected the TG2 and TG4 mouse lines that exhibited medium and high expression levels of Sike, respectively, for subsequent experiments. The increased levels of Sike in the TG2 or TG4 mice did not cause detectable changes in cardiac structure or function at baseline. The *Sike* TG2 and TG4 mice, together with age-matched non-transgenic (NTG) mice, were subjected to sham or AB surgery, and extensive examinations were conducted 8 weeks after surgery. The AB-induced cardiac hypertrophic responses were markedly blunted in both the TG2 and TG4 mice compared with the NTG controls, as indicated by the decreased HW/BW, LW/BW and HW/TL ratios in the TG2 and TG4 mice compared with the NTG mice (Fig. 3d–f). AB-triggered cardiac dilation and dysfunction were also inhibited in the TG2 and TG4 mice, compared with the NTG mice (Fig. 3g). Consistently, the TG2 and TG4 mice exhibited a smaller heart size, fewer enlarged cardiomyocytes, less fibrosis and lower mRNA expression of hypertrophic and fibrotic genes than the NTG mice (Fig. 3h–k). These results indicated that Sike overexpression in the heart blunted the cardiac hypertrophy and fibrosis induced by pressure overload.

### SIKE inhibits agonist-induced cardiac hypertrophy

To determine whether SIKE had similar cardioprotective effects on agonist-induced cardiac hypertrophy, we exposed different Sike genetic mouse models to continuous Ang II infusion for 4 weeks. As expected, Ang II stimulation induced pathological hypertrophy in the control α-MHC-MCM and *Sike*-flox mice, as reflected by increased HW/BW, LW/BW and HW/TL ratios compared with the saline group, which were further exacerbated in the *Sike*-CKO mice (Supplementary Fig. 3a–c). Furthermore, Ang II-induced cardiac dilation and dysfunction were more pronounced in the *Sike*-CKO mice than in the control mice (Supplementary Fig. 3d). Consistently, larger cross-sectional heart size, more severe fibrosis and greater left ventricle (LV) collagen volume in the *Sike*-CKO mice were observed in comparison with the controls (Supplementary Fig. 3e–g). Conversely, the extent of Ang II-induced cardiac hypertrophy and fibrosis was substantially attenuated in the Sike-overexpressing mice compared with the NTG controls at 4 weeks after Ang II infusion (Supplementary Fig. 3h–n). These findings suggest that Sike also regulates the development of Ang II-triggered cardiac hypertrophy and remodelling *in vivo*.

**Figure 4:**
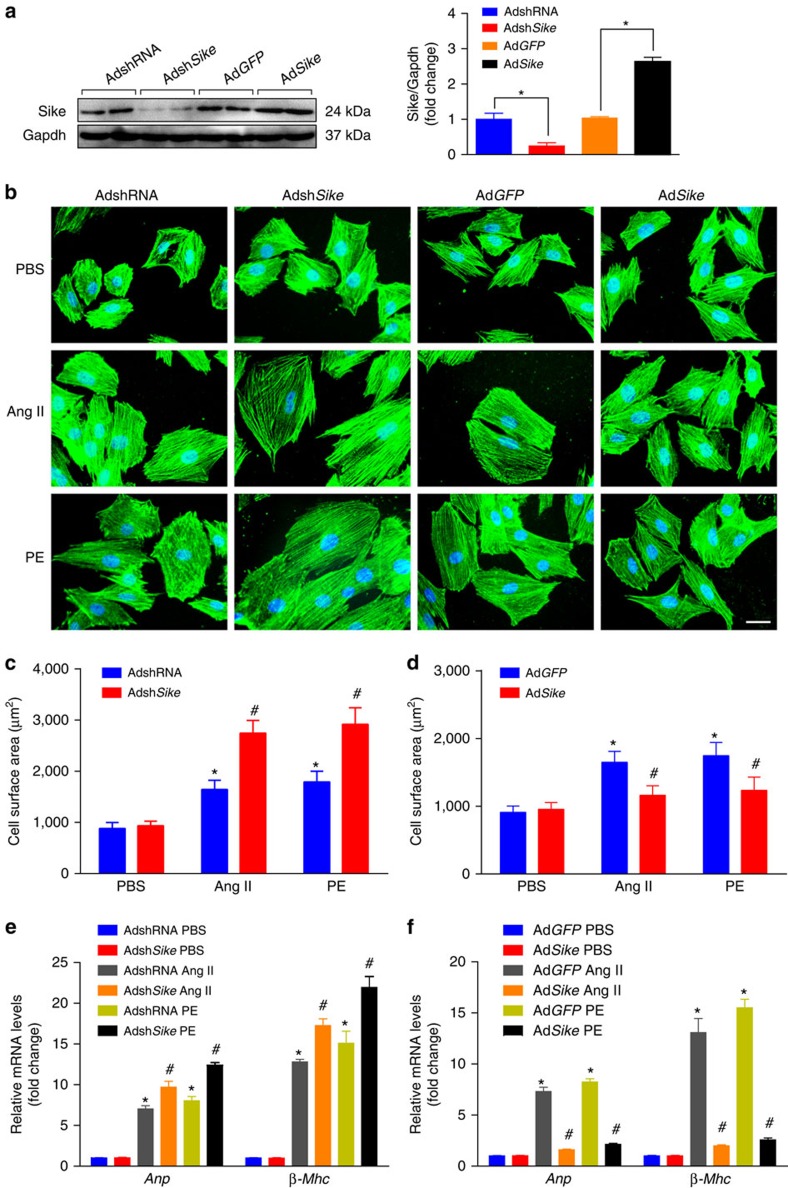
Effects of Sike on Ang II- or PE-induced cardiac cell hypertrophy *in vitro*. (**a**) Immunoblotting and quantification indicated decreased or increased Sike expression mediated by adenovirus that expressed short hairpin RNA targeting *Sike* (Adsh*Sike*) or adenovirus that overexpressed Sike (Ad*Sike*) relative to the corresponding controls and adenovirus that expressed non-targeting shRNA (AdshRNA) and adenovirus that overexpressed GFP (Ad*GFP*), respectively, in cultured rat cardiomyocytes; *n*=4 samples per group; *P<0.05 versus AdshRNA- or Ad*GFP*-infected cells. (**b**) Representative images of α-actinin- (green) and DAPI- (blue) stained cardiomyocytes infected with the indicated adenoviruses followed by 48 h of PBS, Ang II or PE treatment; scale bars, 20 μm. (**c**,**d**) Quantification of the average cell surface area of rat cardiomyocytes infected with AdshRNA or Adsh*Sike* (**c**) and Ad*GFP* or Ad*Sike* (**d**) in response to PBS, Ang II or PE treatment; *n*≥50 cells per group. (**e**,**f**) Transcript levels of hypertrophic marker genes (*Anp* and *β-Mhc*) in AdshRNA- or Adsh*Sike*-infected PBS-, Ang II- or PE-treated cells (**e**) and Ad*GFP*- or Ad*Sike*-infected PBS-, Ang II- or PE-treated cells (**f**); *n*=4 independent experiments. *P<0.05 versus PBS-treated AdshRNA-infected group, ^#^*P*<0.05 versus Ang II- or PE-treated AdshRNA-infected group in **c** and **e**; *P<0.05 versus PBS-treated Ad*GFP*-infected group, ^#^*P*<0.05 versus Ang II- or PE-treated Ad*GFP*-infected group in **d** and **f**. Data are presented as the mean±s.d. Statistical analysis was carried out by one-way analysis of variance.

Considering that *in vivo* cardiac hypertrophy models are complex and encompass both the direct effects of inducing stimuli and the secondary effects of myocardial responses, including cardiac fibrosis and failure, we used the well-established NRCM hypertrophy model to define more specifically the function of Sike in cardiomyocytes. We treated NRCMs with a panel of hypertrophic agonists and examined the effects of Sike on cardiac hypertrophy. NRCMs were infected with Adsh*Sike* to knock down Sike or with Ad*Sike* to overexpress Sike (Fig. 4a). The infected cells were treated with Ang II, PE or PBS for 48 h and then were immunostained with α-actinin antibody to determine cell size or collected for the analysis of fetal cardiac gene expression. Sike depletion caused a significant increase in the cell surface area and the mRNA expression levels of *Anp* and *β-Mhc*, whereas Sike overexpression led to a decrease in these hypertrophic phenotypes, compared with the PBS-treated cells (Fig. 4b–f). These findings confirmed the role of Sike in the modulation of Ang II- or PE-induced cardiomyocyte hypertrophy *in vitro*.

### SIKE regulates AKT signalling during cardiac hypertrophy

**Figure 5:**
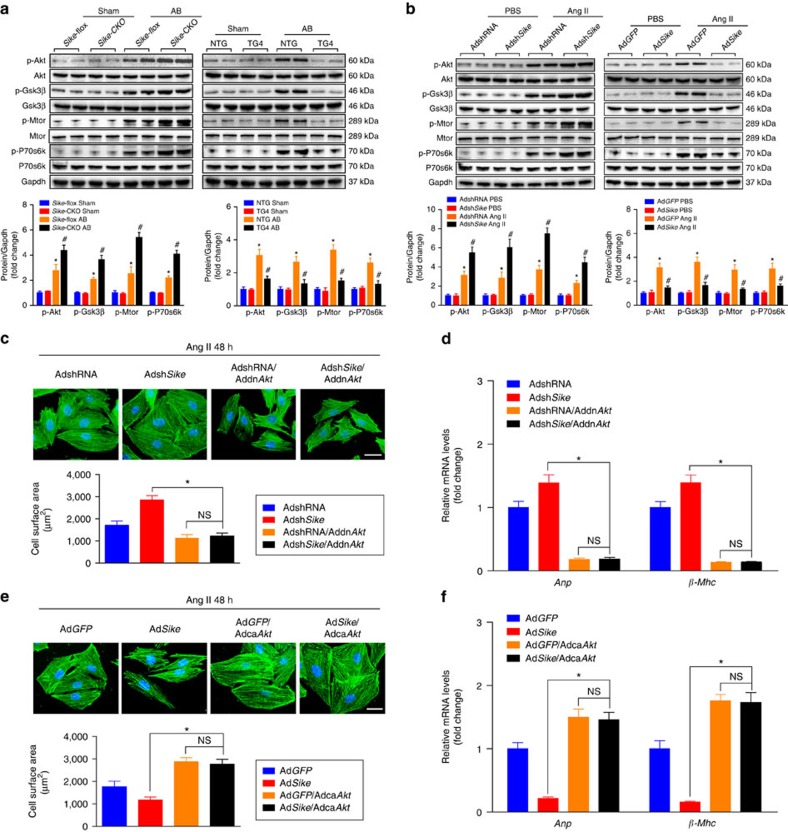
SIKE regulates AKT signalling during cardiac hypertrophy. (**a**) Immunoblot (top) and quantification (bottom) of the activities of Akt/Gsk3β/Mtor/P70s6k signalling components in the hearts of different *Sike* genotypic mice (*Sike*-flox and *Sike*-CKO, NTG and TG4) 4 weeks after sham or AB surgery. *P<0.05 versus the sham-operated *Sike*-flox or NTG group, ^#^*P*<0.05 versus the AB-operated *Sike*-flox or NTG group. *n*=4 mice per group. (**b**) The activities of Akt/Gsk3β/Mtor/P70s6k signalling components in cultured rat cardiomyocytes infected with AdshRNA or Adsh*Sike*, Ad*GFP* or Ad*Sike* and then treated with PBS or Ang II. *P<0.05 versus the PBS-treated AdshRNA- or Ad*GFP*-infected group, ^#^*P*<0.05 versus the Ang II-treated AdshRNA- or Ad*GFP*-infected group. (**c**) Comparison of the average cell surface area of cultured rat cardiomyocytes infected with AdshRNA or Adsh*Sike* alone or in combination with Addn*Akt*, followed by 48 h of Ang II treatment. Representative images of α-actinin- (green) and DAPI- (blue) stained cardiomyocytes and quantification of the average cell area in the indicated groups; *n*≥50 cells per group, scale bar, 20 μm. (**d**) Transcript levels of hypertrophic marker genes (*Anp* and *β-M*hc) in the groups described in **c**; *n*=4 repeats per group. (**e**) Representative images of α-actinin- (green) and DAPI- (blue) stained cardiomyocytes infected with Ad*GFP* or Ad*Sike* alone or in combination with Adca*Akt*, followed by 48 h of Ang II treatment and quantification of the average cell area in the indicated groups; *n*≥50 cells per group, scale bar, 20 μm. (**f**) mRNA levels of the hypertrophic marker genes (*Anp* and *β-M*hc) in the groups described in **e**; *n*=4 independent experiments. *P<0.05 compared between the two indicated groups; NS indicates no significance. Data are presented as the mean±s.d. from at least three independent experiments. Statistical analysis was carried out by one-way analysis of variance.

Next, we explored the cellular mechanisms by which SIKE protects against cardiac hypertrophy. Cardiac pathogenesis is regulated by a series of intracellular signalling pathways, among the most notable of which are MAPKs^14^,^15^. Compared with the sham operation or PBS treatment, a pressure overload *in vivo* or a Ang II challenge *in vitro* caused significantly increased phosphorylation of MAPK members, including Mek1/2, Erk1/2, Jnk1/2 and p38. Unexpectedly, these increases in MAPK activities were unaffected by either Sike deficiency or overexpression in heart tissue or cardiomyocytes (Supplementary Fig. 4a,b), indicating that MAPK signalling might not be involved in SIKE-regulated hypertrophic stresses. The AKT signalling cascade is another signalling pathway that plays an important role in the process of cardiac hypertrophy^16^. Our data indicated that the significantly enhanced phosphorylation of Akt and its downstream targets, including Gsk3β, P70s6k and Mtor, by pro-hypertrophic stimuli were markedly activated in heart tissue and cardiomyocytes because of Sike deficiency, whereas the activation of AKT signalling was blocked by Sike overexpression (Fig. 5a,b). As a key player in AKT signalling, GSK3β can phosphorylate NFAT proteins to stimulate NFAT nuclear export, and therefore antagonists calcineurin-NFAT cascades, a pivotal molecular event during the pathological cardiac remodelling progress^9^,^17^. Consistently, we found that the expression of Nfatc3 and p-Nfatc3 was dramatically decreased in the cytoplasm, whereas the content of Nfatc3 was increased in the nucleus after pressure overload for 4 weeks; moreover, the alteration of these conditions was significantly blunted by Sike overexpression but was further enhanced by Sike deficiency (Supplementary Fig. 5a,b).

To further evaluate whether the SIKE-regulated cardiomyocyte enlargement was AKT dependent, we infected NRCMs with AdshRNA or Adsh*Sike* alone or in combination with Addn*Akt* (dominant negative form of Akt). At the same time, Ad*GFP* or Ad*Sike* alone or in combination with Adca*Akt* (constitutively active form of Akt) was infected into cardiomyocytes. The infected cells were treated with Ang II for 48 h. The results indicated that inhibition of Akt activity completely reversed the pronounced increase in cardiomyocyte size and the expression of fetal cardiac genes induced by Sike downregulation because of Ang II treatment (Fig. 5c,d). Conversely, the activation of AKT by infection with Adca*Akt* impaired the protective functions of Sike overexpression and exhibited a further enlarged cardiomyocyte size and higher mRNA levels of fetal cardiac genes, compared with the Ad*GFP*-infected cells (Fig. 5e,f). These data robustly verified the critical involvement of AKT signalling in SIKE-mediated regulation of cardiac hypertrophy.

### SIKE attenuates cardiac remodelling via the TBK1-AKT axis

**Figure 6:**
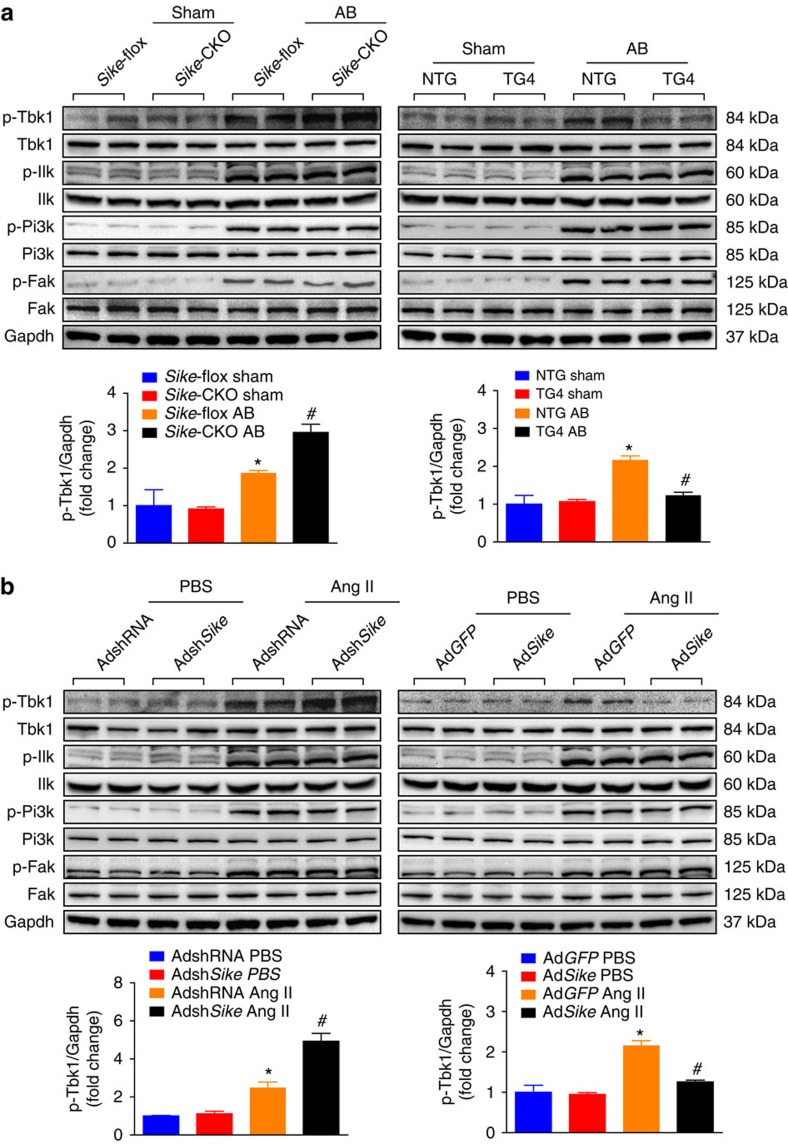
The TBK1-AKT axis is involved in SIKE-regulated cardiac hypertrophy. (**a**) Immunoblots (top) and quantification (bottom) of the active levels of the signaling molecules that regulate the AKT pathway (e.g., Tbk1, Ilk, Pi3k and Fak) in the hearts of different *Sike* genotypic mice (*Sike*-flox and *Sike*-CKO, NTG and TG4) 4 weeks after sham or AB surgery, *P<0.05 versus the sham-operated *Sike*-flox or NTG group, ^#^*P*<0.05 versus the AB-operated *Sike*-flox or NTG group. *n*=4 mice per group. (**b**) Immunoblots (top) and quantification (bottom) of the activities of the signaling proteins upstream of Akt in cultured NRCMs infected with AdshRNA or Adsh*Sike*, Ad*GFP* or Ad*Sike*, followed by PBS or Ang II treatment. *P<0.05 versus the PBS-treated AdshRNA- or Ad*GFP*-infected group; ^#^P<0.05 versus the Ang II-treated AdshRNA- or Ad*GFP*- infected group. Data are presented as the mean±s.d. from at least three independent experiments. Statistical analysis was carried out by one-way analysis of variance.

**Figure 7:**
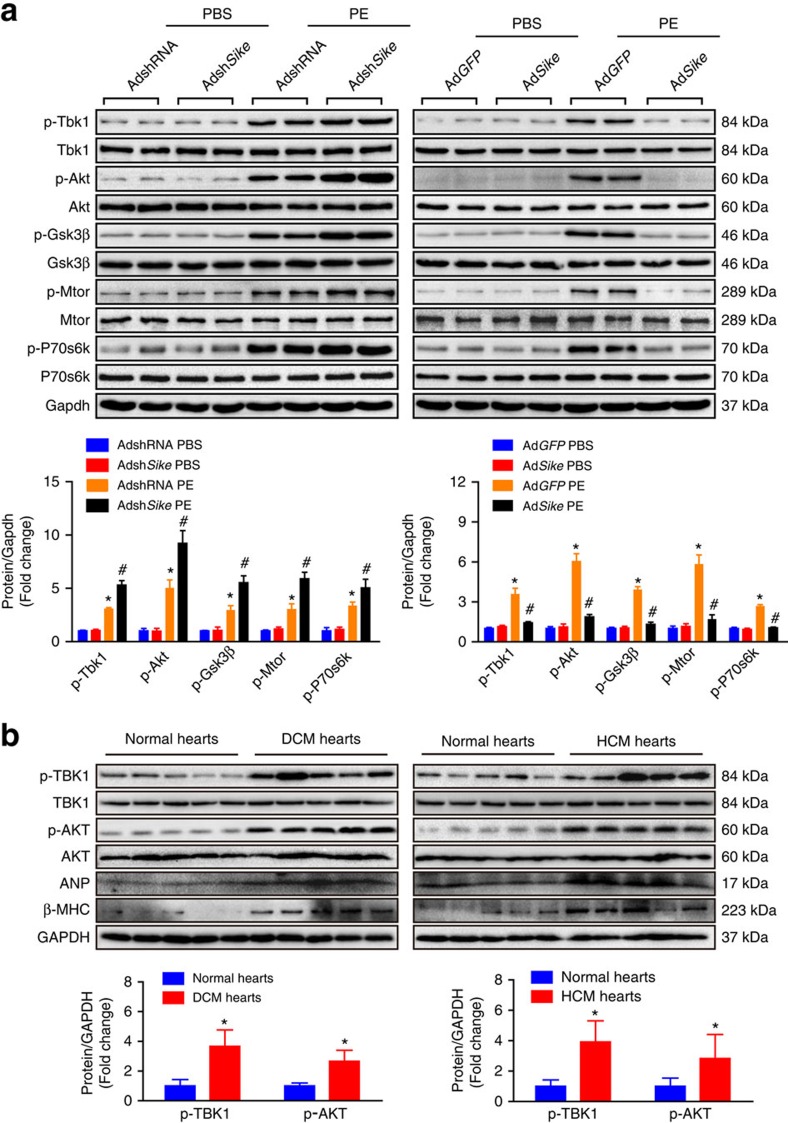
SIKE regulates cardiac remodeling via the TBK1-AKT signaling. (**a**) The active levels of Tbk1 and Akt signaling components in cultured NRCMs infected with AdshRNA or Adsh*Sike*, AdGFP or Ad*Sike* and then treated with PBS or PE. *P<0.05 versus the PBS-treated AdshRNA- or Ad*GFP*-infected group; ^#^*P*<0.05 versus the PE-treated AdshRNA- or Ad*GFP*- infected group. (**b**) The active levels of TBK1 and AKT and the expression levels of ANP and β-MHC in normal, DCM or HCM human hearts, *n*=5 samples per group; *P<0.05 versus normal hearts. Data are presented as the mean±s.d. from at least three independent experiments. For **a**, statistical analysis was carried out by one-way analysis of variance; for **b**, statistical analysis was carried out by Student's two-tailed *t*-test.

To investigate the upstream factors regulating AKT activity during SIKE-modulated cardiac remodelling, we examined the influence of SIKE on the protein expression of potential AKT upstream molecules, including TBK1, integrin-linked kinase (ILK), PI3K and focal adhesion kinase (FAK). As shown in Fig. 6a,b, all of the phosphorylated expression levels of the measured factors were significantly increased under hypertrophic stresses, whereas only Tbk1 activity was inhibited by Sike overexpression but promoted by Sike deficiency. Consistently, the inhibitory function of Sike in the activation of Tbk1-Akt-Mtor/Gsk3β signalling was further validated in PE-stimulated NRCMs (Fig. 7a), demonstrating that Sike regulated the Tbk1/Akt signalling axis in response to different hypertrophic stimulations. To illustrate the relevance of this signalling axis in human cardiac remodelling pathologies, we analysed the active levels of TBK1/AKT signalling in normal and HCM or DCM human hearts. As anticipated, the phosphorylated levels of TBK1 and AKT in the HCM and DCM hearts were significantly increased, along with dramatically elevated expression of ANP and β-MHC, compared with normal hearts (Fig. 7b). Thus, these data from mice and primary cardiomyocytes, together with the results from humans, collectively provide evidence for the involvement of the TBK1-AKT signalling cascade in SIKE-mediated pathological cardiac hypertrophy.

**Figure 8:**
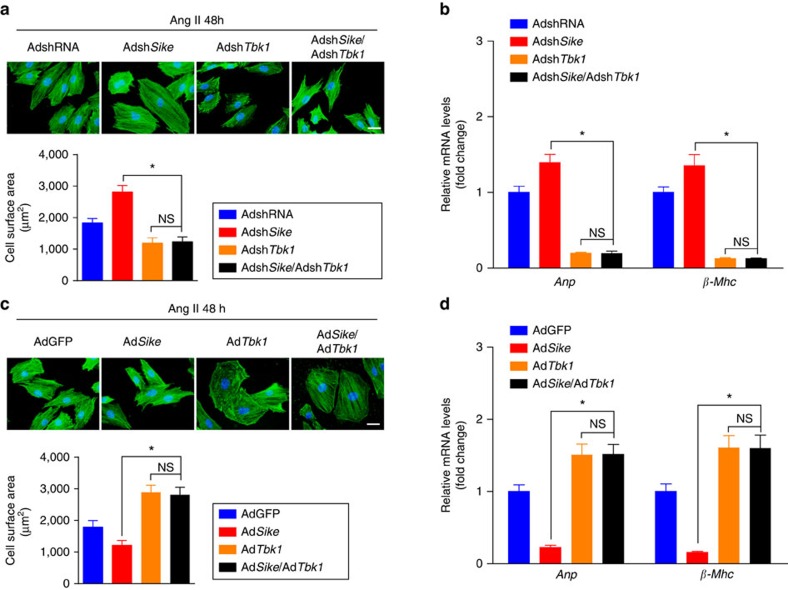
SIKE-regulated cardiac hypertrophy is dependent on TBK1. (**a**) Cultured NRCMs were infected with AdshRNA or Adsh*Sike* alone or in combination with Adsh*Tbk1*, followed by 48 h of Ang II treatment; representative images of α-actinin-stained (green) and DAPI-stained (blue) cardiomyocytes and the average cell surface area in the indicated groups; *n*≥50 cells per group, scale bar, 20 μm. (**b**) Transcript levels of hypertrophic marker genes (*Anp* and *β-Mhc*) in the groups described in **a**; *n*=4 repeats per group. (**c**) Cultured NRCMs were infected with Ad*GFP* or Ad*Sike* alone or in combination with Ad*Tbk1*, followed by 48 h of Ang II treatment; representative images of α-actinin-stained (green) and DAPI-stained (blue) cardiomyocytes and the average cell surface area in the indicated groups. *n*≥50 cells per group, scale bar, 20 μm. (**d**) mRNA levels of the hypertrophic marker genes (*Anp* and *β-Mhc*) in the groups described in **c**; *n*=4 independent experiments. *P<0.05 compared between the two indicated groups; NS indicates no significance. Data are presented as the mean±s.d. from at least three independent experiments. Statistical analysis was carried out by one-way analysis of variance.

After confirming the participation of TBK1 and downstream AKT signalling in the progression of pathological cardiac hypertrophy, we infected NRCMs with Adsh*Sike* alone or in combination with Adsh*Tbk1*, followed by the treatment of Ang II for 48 h to examine the requirement of Tbk1 in the regulatory effects of Sike on cardiac remodelling. Compared with AdshRNA-infected cells, Ang II induced a further increase in cardiomyocyte size and the expression of fetal cardiac genes in Adsh*Sike*-infected cells; in contrast, this increase was completely eliminated in either the individual Adsh*Tbk1*-infected cells or the cells infected simultaneously with Adsh*Sike* and Adsh*Tbk1* (Fig. 8a,b). Conversely, the reduced cardiomyocyte size and mRNA levels of the fetal genes in the Ad*Sike*-infected cells were fully reversed by Ad*Tbk1* infection (Fig. 8c,d). These results indicated the dependent role of TBK1 in SIKE-mediated cardioprotection.

### SIKE-mediated cardioprotection depends on TBK1 inhibition

**Figure 9:**
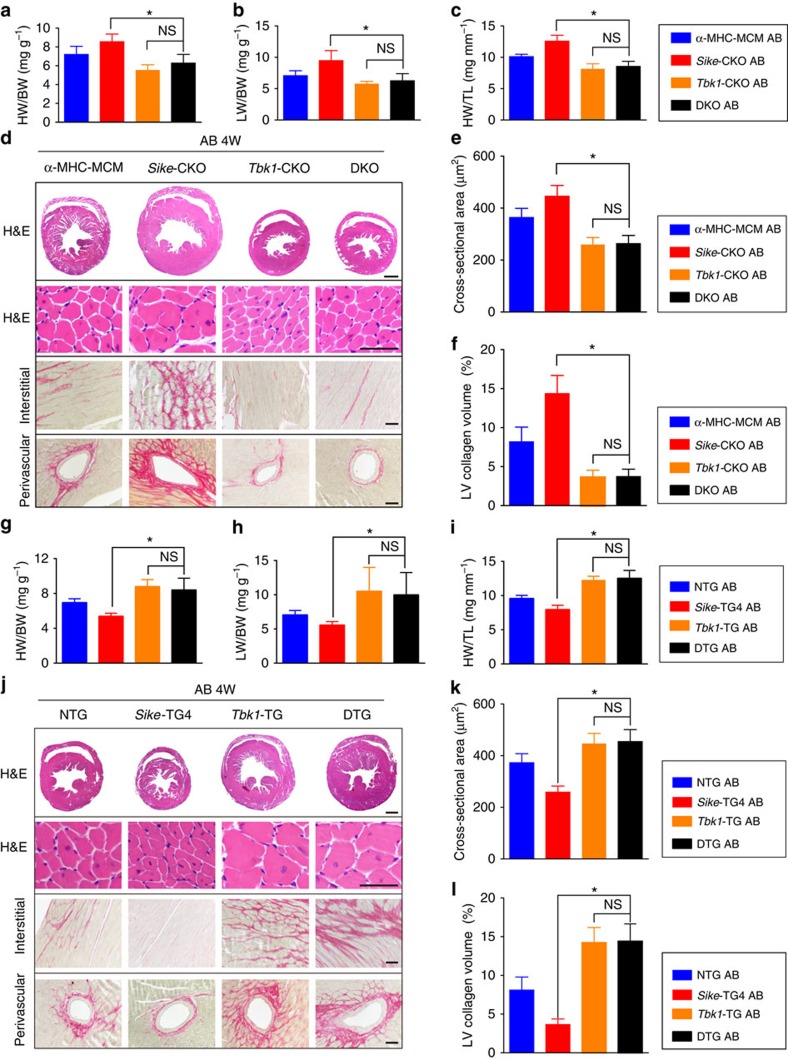
SIKE-mediated cardioprotection relies on the inactivation of TBK1 signalling. (**a**–**c**) Comparison of the HW/BW (**a**), LW/BW (**b**) and HW/TL (**c**) ratios in different genotypic mice subjected to AB surgery, *n*=10–12 mice per group. (**d**) Histological analyses of whole hearts (the first row; scale bar, 1,000 μm) and heart sections from the indicated groups stained with H&E (the second row; scale bar, 50 μm) or PSR (the third and fourth row; scale bars, 50 μm) 4 weeks after the AB surgery, *n*=6–8 mice per group. (**e**) Comparison of the cross-sectional area of cardiomyocytes in the indicated groups, *n*≥100 cells per group. (**f**) Comparison of the LV collagen volume in the indicated groups, *n*≥40 fields per group. (**g**–**i**) Comparison of the HW/BW (**g**), LW/BW (**h**) and HW/TL (**i**) ratios in the different genotypic mice that underwent AB surgery, *n*=11–13 mice per group. (**j**) Histological analyses of whole hearts (the first row; scale bar, 1,000 μm) and heart sections from the indicated groups stained with H&E (the second row; scale bar, 50 μm) or PSR (the third and fourth row; scale bars, 50 μm) 4 weeks after AB surgery, *n*=6–8 mice per group. (**k**) Comparison of the cross-sectional area of cardiomyocytes from the indicated groups, *n*≥100 cells per group. (**l**) Comparison of the LV collagen volume in the indicated groups, *n*≥40 fields per group. *P<0.05 compared between the two indicated groups; NS indicates no significance. Data are presented as the mean±s.d. from at least three independent experiments. Statistical analysis was carried out by one-way analysis of variance.

To evaluate the necessity of TBK1 in SIKE-regulated cardiac hypertrophy, we generated a conditional *Tbk1*-knockout mouse line and crossed this line with an *α-MHC* promoter-driven *Cre* TG strain to obtain a mouse line with cardiac-specific ablation of Tbk1 (*Tbk1*-CKO; Supplementary Fig. 6a). We further produce a mouse line with the loss of both Sike and Tbk1 in the heart (double knockout (DKO)). Individual or double ablation of Sike and/or Tbk1 was confirmed in hearts obtained from different genotypic mice (Supplementary Fig. 6b). Interestingly, the aggravation of pressure overload-induced hypertrophy identified in the *Sike*-CKO mice was effectively prevented by Tbk1 deficiency, evidenced by the reduced HW/BW, LW/BW and HW/TL ratios (Fig. 9a–c), improved cardiac function (Supplementary Fig. 6c), smaller cardiomyocyte size (Fig. 9d,e), mitigated fibrosis (Fig. 9d,f) and decreased mRNA levels of fetal cardiac genes (Supplementary Fig. 6d) in the AB-operated *Tbk1*-CKO and DKO mice compared with the α-MHC-MCM mice or *Sike*-CKO mice. Furthermore, no significant phenotypic differences were identified between the *Tbk1*-CKO and DKO mice, suggesting complete abolition of the exacerbating effect of Sike deficiency by Tbk1 ablation.

We also created cardiac-specific *Tbk1*-TG mice and crossed this mouse line with the *Sike*-TG4 line to produce double transgenic mice (DTG; Supplementary Fig. 6e). Increased expression of Sike and/or Tbk1 was confirmed in the hearts of different genotypic mice (Supplementary Fig. 6f). Similarly, age-matched NTG, *Sike*-TG4, *Tbk1*-TG and DTG mice were subjected to AB surgery, and extensive examinations were performed on these mice 4 weeks later. In contrast with the ameliorated phenotypes in the *Sike*-TG4 mice compared with the NTG mice, individual Tbk1 or double Sike and Tbk1 overexpression in the heart promoted AB-induced cardiac hypertrophy and fibrosis, along with increased HW/BW, LW/BW and HW/TL ratios (Fig. 9g–i), worsened cardiac function (Supplementary Fig. 6g), larger heart and cardiomyocyte sizes (Fig. 9j,k), aggravated fibrosis (Fig. 9j,l) and increased transcript levels of fetal cardiac genes (Supplementary Fig. 6h). These data indicated that hyperactivation of Tbk1 contributed to the development of cardiac hypertrophy and heart failure in Sike-overexpressing mice. Thus, we could conclude that the SIKE-regulated amelioration of cardiac hypertrophy was dependent on its inhibition of TBK1 activation.

### SIKE regulates TBK1 through direct physical interaction

**Figure 10:**
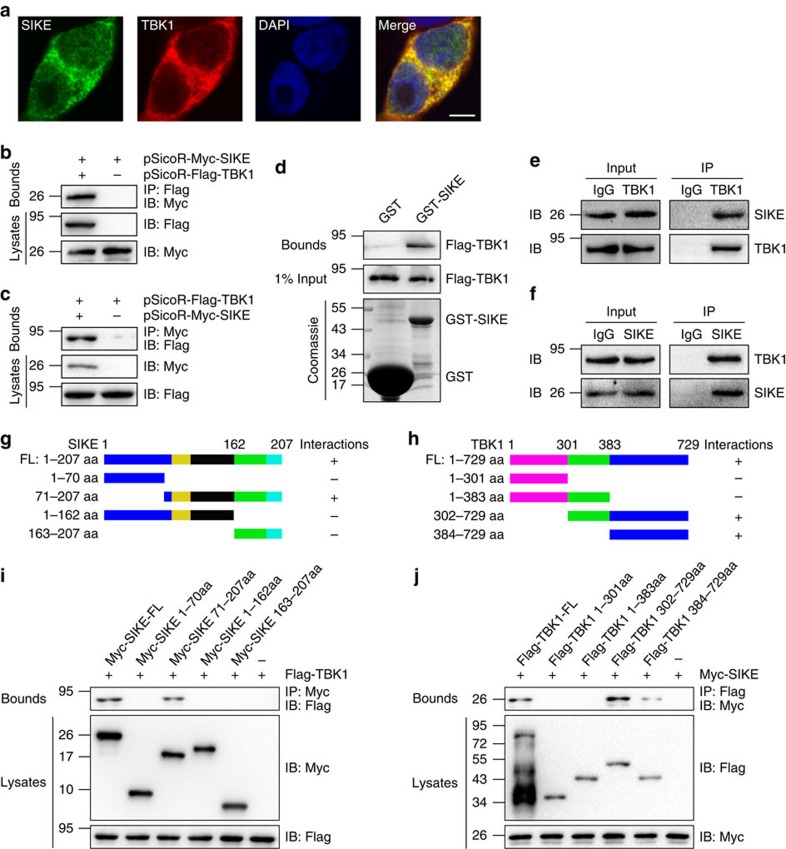
SIKE regulates TBK1 through direct physical interaction. (**a**) Representative confocal images demonstrate the co-localization of TBK1 and SIKE in the cytoplasm of HEK293T cells. (**b**,**c**) HEK293T cells were co-transfected with indicated plasmids. After 48 h, cells were harvested, and cellular lysates were subjected to IP with antibodies against Flag (**b**) or Myc (**c**). (**d**) Immunoblot of a GST pull-down assay, in which exogenous GST-SIKE fusion protein purified from *E. coli* was incubated with immunopurified Flag-TBK1 from HEK293T cells. (**e**,**f**) Immunoblotting with a SIKE or TBK1 antibody was performed on co-IP of Tbk1 from cardiac cell lysates using a TBK1 antibody (**e**) or SIKE antibody (**f**). (**g**,**h**) Schematic of the full-length and truncated mutants of SIKE (**g**) and TBK1 (**h**). (**i**,**j**) Immunoblotting was performed with indicated antibodies, following co-IP of the full-length and truncated mutants of SIKE (**i**) or TBK1 (**j**) from HEK293T whole-cell lysates using a Myc or Flag antibody, respectively.

The TBK1-dependent manner of SIKE-regulated pathological cardiac remodelling inspired us to investigate the molecular communications between SIKE and TBK1. Based on a confocal approach, we identified complete co-localization of SIKE with TBK1 in the cytoplasm of the HEK293T cells transfected with pEGFP-SIKE and pmCherry-TBK1 (Fig. 10a). Furthermore, direct interaction of SIKE with TBK1 was identified in co-immunoprecipitation (co-IP) experiments when Myc-tagged SIKE and Flag-tagged TBK1 were co-transfected into HEK293T cells (Fig. 10b,c), consistent with a previous report^13^. Moreover, this interaction between SIKE and TBK1 was confirmed by a glutathione *S*-transferase (GST) pull-down assay (Fig. 10d). Importantly, endogenous interaction between Sike and Tbk1 was identified in primary cardiomyocytes (Fig. 10e,f). Considering that SIKE and TBK1 showed a potent influence on stressed hearts, but not under basal conditions, additional endogenous co-IP experiments were performed using heart tissue collected from wild-type mice 4 weeks after treatment with sham or AB surgery. As shown in Supplementary Fig. 7a, Sike and Tbk1 could directly bind to each other under basal conditions; however, in the hypertrophic hearts induced by pressure overload, the binding between Sike and Tbk1 was dramatically reduced compared with that in sham controls. To further identify the binding domain responsible for the SIKE–TBK1 interaction, we created a series of SIKE and TBK1 mutants and performed domain-mapping experiments (Fig. 10g,h). The results demonstrated that the C-terminal region (the amino acids from 71 to 207, including the coiled–coil domain) of SIKE and the C-terminal region (the amino acids from at least 384 to 729, including the coiled–coil domain) of TBK1 were required for this interaction (Fig. 10i,j). Thus, these findings suggest the presence of an authentic interaction between SIKE and TBK1 that facilitates the negative regulation of TBK1 by SIKE.

**Figure 11:**
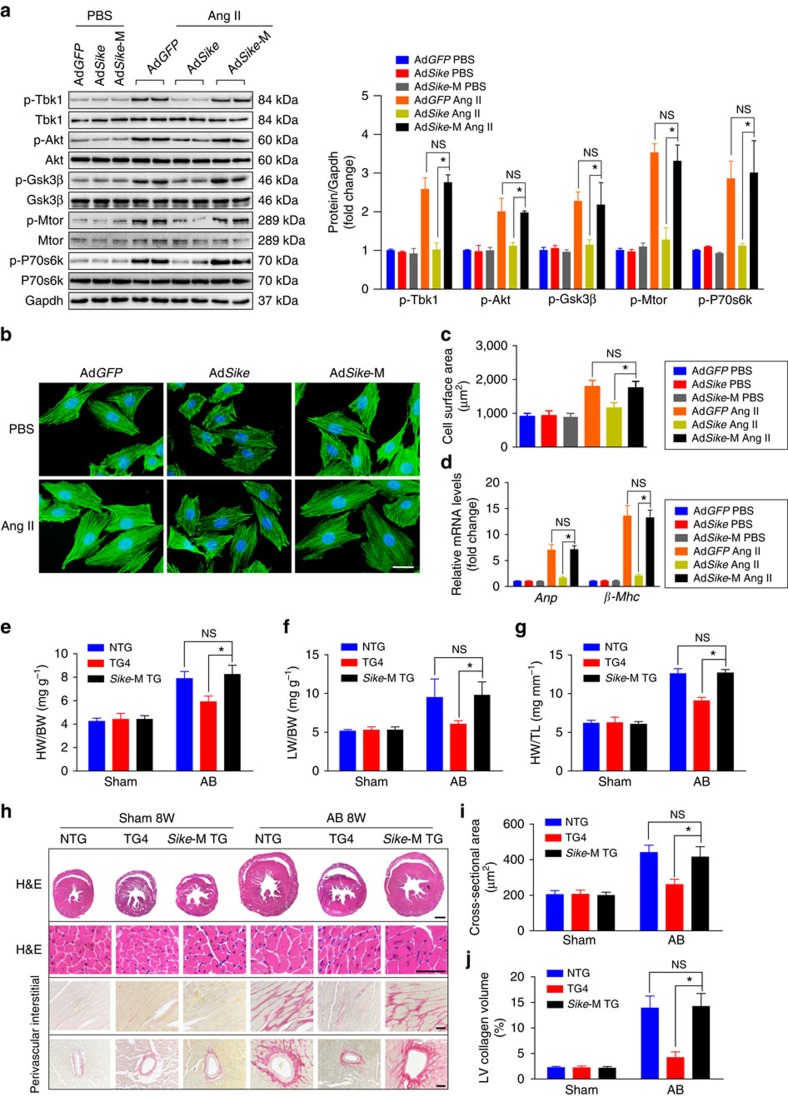
SIKE ameliorate cardiac remodelling dependent on SIKE-TBK1 interaction. (**a**–**d**) NRCMs were infected with the indicated adenovirus, followed by PBS or Ang II treatment. Immunoblotting of the active levels of Tbk1 and Akt signalling components was performed (**a**); NRCMs were stained with an α-actinin antibody and DAPI to measure the average cell surface area (**b**,**c**; *n*≥50 cells per group, scale bar, 20 μm); the mRNA levels of the hypertrophic marker genes were compared in the indicated groups (**d**). (**e**–**g**) Comparison of the HW/BW (**e**), LW/BW (**f**) and HW/TL (**g**) ratios in different genotypic mice subjected to sham or AB surgery, *n*=10–13 mice per group. (**h**) Histological analyses of whole hearts (the first row; scale bar, 1,000 μm) and heart sections from the indicated groups stained with H&E (the second row; scale bar, 50 μm) or PSR (the third and fourth row; scale bars, 50 μm) 8 weeks after sham or AB surgery, *n*=6–8 mice per group. (**i**) Comparison of the cross-sectional area of cardiomyocytes, *n*≥100 cells per group. (**j**) Comparison of the LV collagen volume in the indicated groups, *n*≥40 fields per group. *P<0.05 compared between the two indicated groups; NS indicates no significance. Data are presented as the mean±s.d. from at least three independent experiments. Statistical analysis was carried out by one-way analysis of variance.

To illustrate the functional significance of SIKE–TBK1 interaction in hypertrophic pathologies, an adenovirus expressing the Sike mutant form and lacking the binding region for Tbk1 (Ad*Sike*-M) was generated and used to infect NRCMs. After Ang II treatment, adenovirus-mediated Sike overexpression strongly inhibited the increased expression levels of phosphorylated Tbk1, Akt, Gsk3β, P70s6k and Mtor, whereas the mutant Sike lost this inhibitory effect on the activation of Tbk1-Akt signalling (Fig. 11a). Unsurprisingly, the inhibitory effects of Sike on the enlargement of cell size and the mRNA expression of *Anp* and *β-Mhc* were not observed in the Ad*Sike*-M-infected cardiomyocytes (Fig. 11b–d).

Next, we investigated whether this phenomenon observed *in vitro* also occurred *in vivo*. Thus, we generated cardiac-specific *Sike*-M TG mice (Supplementary Fig. 7b,c), which were then subjected to AB surgery. Extensive examinations indicated that, unlike the ameliorated AB-induced hypertrophic pathologies in the *Sike*-TG mice, the extent of cardiac hypertrophy and fibrosis in the *Sike*-M TG mice was similar to that in the NTG mice, as evidenced by the increased heart and cardiomyocyte sizes, aggravated cardiac dysfunction, exacerbated extent of fibrosis and elevated expression of fetal cardiac genes compared with the *Sike*-TG mice 8 weeks after AB surgery (Fig. 11e–j and Supplementary Fig. 7d,e).

Previous studies have reported that SIKE could function as a substrate of TBK1 in *in vitro* kinetic analyses^18^. Thus, we further examined the potential influence of SIKE phosphorylation status on TBK1. However, inconsistent with the aforementioned study, phosphorylated Sike expression was not significantly influenced by pro-hypertrophic stresses, and it failed to be altered significantly by the overexpression of Tbk1 *in vitro* and *in vivo* (Supplementary Fig. 8a,b). In addition, to explore other potential mechanisms underlying SIKE-regulated cardiac hypertrophy, we also measured the regulation of SIKE in the interaction of TBK1 with TRAF3, a factor with binding capacity to TBK1 and a positive functional role during cardiac hypertrophy^19^. Our co-IP results showed that upregulation of Sike did not significantly mediate the interaction between Tbk1 and Traf3 with or without Ang II challenge in cardiomyocytes (Supplementary Fig. 9). Taken together, these *in vitro* and *in vivo* data demonstrated that SIKE could directly bind to TBK1 under basal conditions but dissociate from TBK1 in pathologically hypertrophic hearts. The SIKE–TBK1 interaction was indispensable for SIKE-mediated inhibition of cardiac hypertrophy.

### SIKE alleviates cardiac remodelling in larger animals

**Figure 12:**
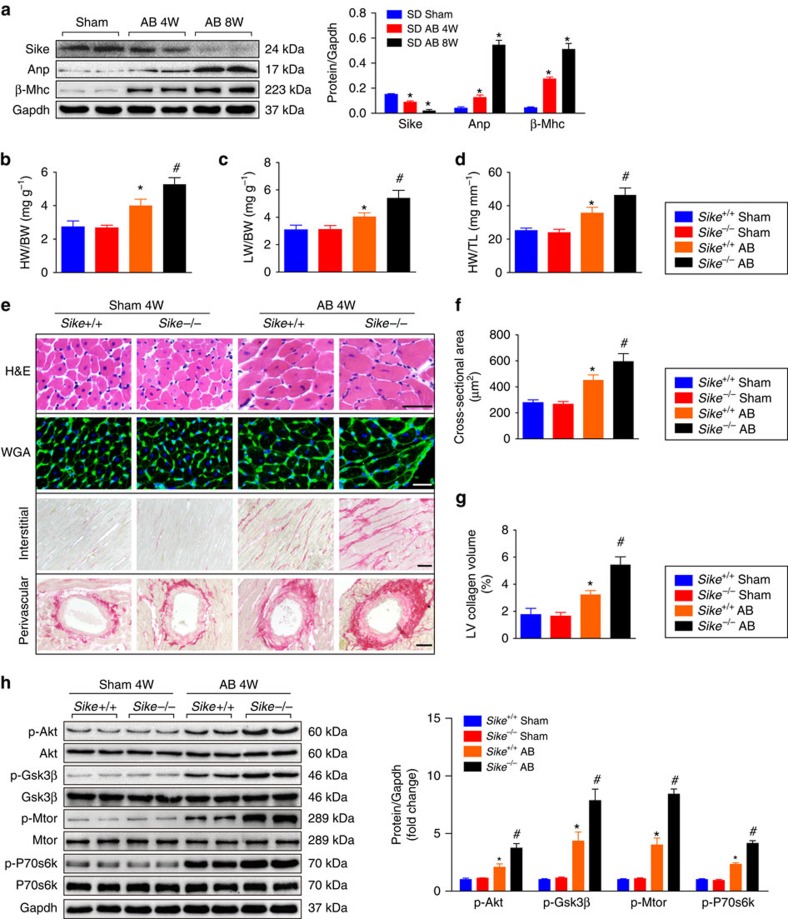
*Sike*-null rats exacerbate pressure overload-induced cardiac hypertrophy. (**a**) Immunoblot and quantification of Sike, Anp and β-Mhc protein levels in the hearts of s.d. rats 4 or 8 weeks after sham or AB surgery, *P<0.05 versus the sham-operated group. *n*=4 rats per group. (**b**–**d**) Comparison of the HW/BW (**b**), LW/BW (**c**) and HW/TL (**d**) ratios in different genotypic rats (*Sike*^*+/+*^ and *Sike*^*−/−*^) subjected to sham or AB surgery, *n*=10–12 rats per group. (**e**) Histological analyses of heart sections stained with H&E (the first row; scale bar, 50 μm), WGA (the second row; scale bar, 20 μm) or PSR (the third and fourth row; scale bars, 50 μm) in the indicated groups 4 weeks after sham or AB surgery, *n*=6 or 7 rats per group. (**f**) Comparison of the cross-sectional area of cardiomyocytes in the indicated groups, *n*≥100 cells per group. (**g**) Comparison of the LV collagen volume in the indicated groups, *n*≥40 fields per group. (**h**) Immunoblotting and quantification of the active levels of Akt signalling components, *n*=4 independent experiments. *P<0.05 versus the sham-operated *Sike*^*+/+*^ rat group; ^#^*P*<0.05 versus the AB-operated *Sike*^*+/+*^ rat group. Data are presented as the mean±s.d. from at least three independent experiments. Statistical analysis was carried out by one-way analysis of variance.

After fundamentally confirming the important role of Sike in protecting against cardiac hypertrophy in mice, we further evaluated the potential utilization of SIKE as a key therapeutic target by investigating the role of SIKE in larger animals, that is, rats and monkeys. Pressure overload-induced cardiac hypertrophy was established in Sprague–Dawley (SD) rats through AB surgery. Consistent with that observed in the mouse cardiac hypertrophy model, a time-dependent decrease in the Sike level was identified in the AB-operated SD rats compared with the sham-operated rats at 4 or 8 weeks after AB surgery (Fig. 12a). To observe the functional role of Sike in the development of rat cardiac hypertrophy, we subsequently adopted a transcription activator-like effector nuclease (TALEN)-based knockout strategy to generate *Sike*-null rats^20^. A TALEN pair was designed to target E1 of Sike, as described in Supplementary Fig. 10a,b, and the *in vitro* transcribed *Sike*-TALEN mRNAs were subsequently injected into fertilized rat oocytes to disrupt *Sike*. The sequencing and PCR results demonstrated the deletion of one or both *Sike* alleles in several founder rats (Supplementary Fig. 10c,d), and the loss of the Sike protein was confirmed by western blot (Supplementary Fig. 10e). *Sike*^*−/−*^ rats showed no phenotypic abnormalities under basal conditions. However, at 4 weeks after AB surgery, the heart and lung masses in the *Sike*^*−/−*^ rats were markedly increased, accompanied by significantly compromised cardiac function, increased cardiomyocyte size and accelerated fibrosis, compared with *Sike*^*+/+*^ controls (Fig. 12b–g and Supplementary Fig. 10f). Furthermore, Sike deficiency promoted AB-triggered activation of Tbk1-Akt signalling (Fig. 12h). Taken together, Sike deficiency in rats duplicated the exacerbated hypertrophic pathologies induced by the pressure overload present in *Sike*-CKO mice.

**Figure 13:**
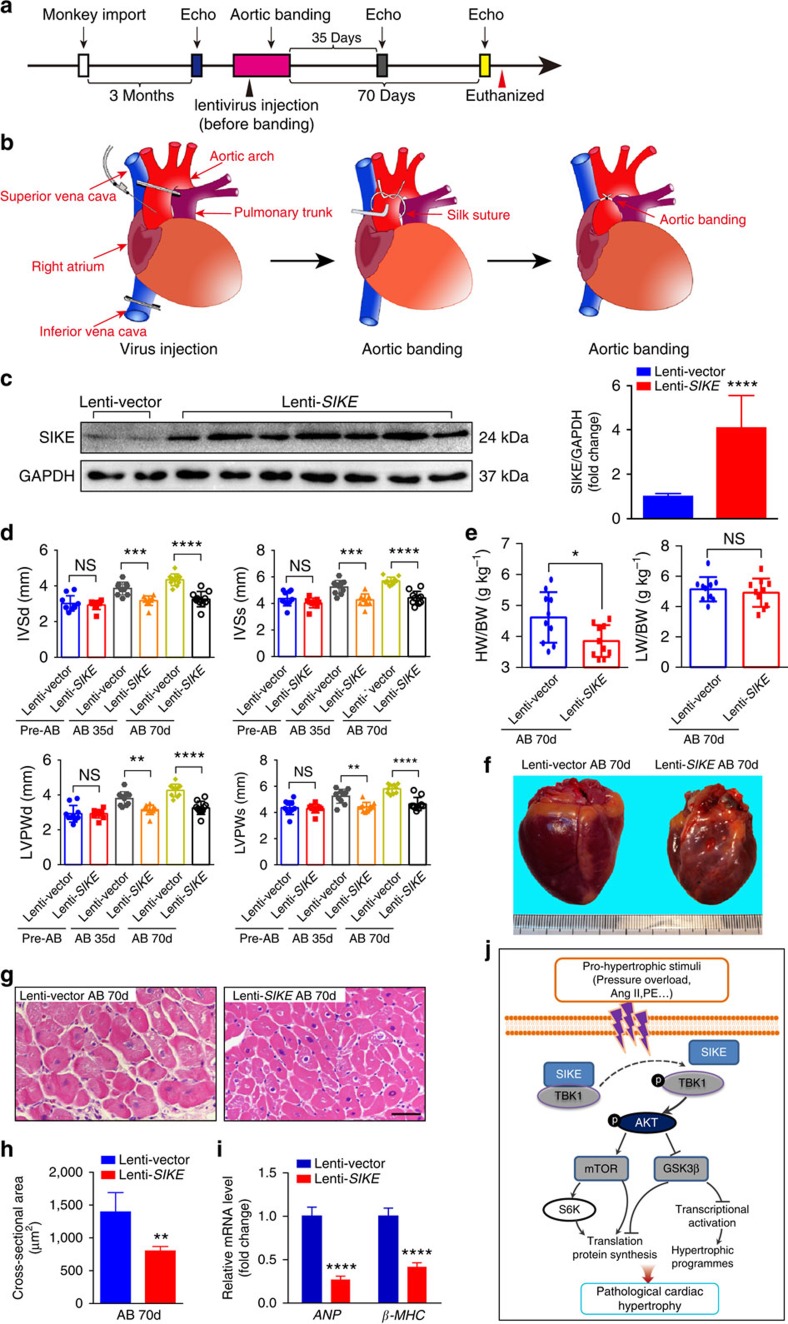
Cardiac SIKE upregulation ameliorates AB-induced hypertrophy in monkeys. (**a**) Time course of the operations (lentivirus injection and AB binding) and echocardiographic analysis. (**b**) Schematic of virus injection and AB surgery on the heart. (**c**) Immunoblotting and quantification of SIKE expression in the Lenti-Vector- or Lenti-*SIKE*-injected group. (**d**) Comparison of the echocardiographic parameters (interventricular septal thickness at end diastole (IVSd), interventricular septal thickness at end systole (IVSs), left ventricular posterior wall thickness at end diastole (LVPWd) and left ventricular posterior wall thickness at end systole (LVPWs)) at different time points in the indicated monkey groups (Lenti-Vector- or Lenti-*SIKE*-injected) that received AB surgery. (**e**) Comparison of the HW/BW and LW/BW ratios in the indicated groups. (**f**–**h**) Representative appearance of the whole hearts (**f**), H&E-stained heart sections (**g**, scale bar, 50 μm) and the cross-sectional area of cardiomyocytes (**h**, *n*≥100 cells per group) for the indicated groups 70 days after AB surgery. (**i**) mRNA levels of the hypertrophic marker genes (*ANP* and *β-MHC*) in the indicated groups. For **c**–**i**, *n*=10 or 11 monkeys per group. *P<0.05, **P<0.01, ***P<0.001 and ****P<0.0001 versus the AB-operated Lenti-Vector-injected group; NS indicates no significance. Data are presented as the mean±s.d. from at least three independent experiments. For **c**, **e**, **h** and **i**, statistical analysis was carried out by Student's two-tailed *t*-test; for **d**, statistical analysis was carried out by one-way analysis of variance. (**j**) Schematic diagram of the molecular mechanisms underlying SIKE-regulated cardiac hypertrophy.

Finally, we investigated the cardioprotective role of SIKE in monkeys as a large animal model. As illustrated in the experimental paradigm (Fig. 13a), monkeys were randomly assigned to two groups after an initial echocardiographic examination. After injection with a lentivirus that expressed SIKE (Lenti-*SIKE*) or a control virus (Lenti-Vector), the monkeys were subjected to AB surgery for 70 days (Fig. 13a,b), which was confirmed by the measured aortic diameters and calculated stenosis percentages (Supplementary Fig. 11 and Supplementary Table 1). The success of SIKE overexpression by Lenti-*SIKE* injection was confirmed by measuring SIKE protein expression by western blot analysis (Fig. 13c). After completion of the second and third echocardiographic examinations on days 35 and 70, respectively, the monkeys in the indicated groups were killed for histological analyses. As shown in Fig. 13d, echocardiographic measurements of ventricle wall thickness were gradually increased after aortic constriction in control Lenti-Vector-infected monkeys, indicating progressive development of cardiac hypertrophy. Importantly, SIKE overexpression delayed the progress of pressure overload-triggered ventricle wall hypertrophy compared with the controls. Moreover, compared with the control virus-injected group, the monkeys in the lentivirus SIKE-injected group consistently exhibited decreased heart size and cardiac cross-setional area and significantly reduced transcript levels of *ANP* and *β-MHC* (Fig. 13e–i). These findings were in agreement with the effect of SIKE overexpression in a pathological cardiac hypertrophy mouse model after pressure overload challenge. Together, our results show that SIKE protects against hypertrophic pathologies in larger animal models, such as rats and monkeys.

## Discussion

The increased incidence of heart failure and the resultant economic impact necessitate the exploration of efficient molecular targets to improve preventive and therapeutic strategies. Our present study provides evidence that SIKE functions as a negative regulator of pathological cardiac hypertrophy and fibrosis based on substantial *in vitro* cellular and *in vivo* animal models. Our results show that SIKE interacts with TBK1 in unstressed hearts; however, the binding of SIKE to TBK1 is dramatically reduced upon pro-hypertrophic stimulus. The SIKE–TBK1 interaction and the subsequent restriction of TBK1-AKT signalling demonstrate the cardioprotective role of SIKE in the development of cardiac hypertrophy (Fig. 13j). Importantly, the regulatory effect of SIKE on pathological cardiac hypertrophy and the underlying mechanisms are further validated in rat and monkey models, as well as in human heart samples, suggesting the promising application of SIKE as a therapeutic target for pathological cardiac hypertrophy and heart failure.

SIKE was originally identified as an IKKɛ-associated protein via yeast two-hybrid screening, and it is ubiquitously expressed in most tissues, including the heart^13^. The profound involvement of IKKɛ in inflammation and immunity suggests a potential role for SIKE in immune diseases^21^,^22^,^23^. However, the functional relevance of SIKE expression in cardiac hypertrophy was unknown. In the present study, decreased SIKE expression was consistently identified in agonist-stimulated cardiomyocytes, in rodent hearts with pressure overload- or Ang II-induced hypertrophy and, in particular, in human heart samples with DCM or HCM, suggesting the functional relevance of SIKE to pathological cardiac hypertrophy. The negative regulation by SIKE of pathological cardiac remodelling was further validated by our gain- and loss-of-function approaches, based on multiple experimental animal models. Deficiency of Sike dramatically promoted pathological cardiomyocyte enlargement, cardiac dysfunction and fibrosis induced by various pro-hypertrophic stimuli, whereas Sike overexpression exhibited the opposite phenotype *in vitro* and *in vivo*.

During our investigations into the signalling pathways responsible for SIKE-regulated pathological cardiac hypertrophy, we demonstrated that the activation of AKT signalling was dramatically suppressed by SIKE upon hypertrophic stress, and the necessity of AKT signalling for the functional role of SIKE in cardiac remodelling was evidenced by the ability of artificial overexpression of Akt to reverse the beneficial effect of Sike on cardiomyocyte enlargement. Furthermore, our screening of potential upstream factors identified TBK1 as the critical link connecting SIKE to AKT signalling under pathological cardiac hypertrophic conditions. The essential function of TBK1 in the suppressive effect of SIKE on cardiac hypertrophy was verified by ‘rescue' experiments using *Sike/Tbk1*-DKO or DTG mice. The inhibitory capacity of Sike on Tbk1 led to the very interesting observation that overexpressed Sike did not counteract the pro-hypertrophic effects of overexpressed Tbk1. Notably, TBK1 is a kinase, and it has a potent capacity for controlling the activation of signalling cascades. Once TBK1 is activated, even to a slight degree, in response to extracellular stimuli, the corresponding downstream molecular cascades can be dramatically activated. Thus, overexpression of Tbk1 leads to striking upregulation of Tbk1-Akt-Mtor/Gsk3β signalling activity upon pro-hypertrophic challenges, which is sufficiently powerful to abolish protective effects of SIKE on pathological cardiac remodelling.

TBK1 activity is modulated in various manners, among which prevention of the formation of functional TBK1-containing complexes is an important regulatory mechanism^24^,^25^,^26^,^27^. Consistent with a previous report^13^, we demonstrated that TBK1 activity was largely mediated by direct SIKE and TBK1 interaction. Under basal conditions, SIKE could directly bind to TBK1 in cultured cardiomyocytes and hearts. However, the binding of SIKE to TBK1 was obviously disrupted upon pro-hypertrophic stimulus. The requirement of the SIKE-TBK1 interaction for SIKE-regulated cardiac hypertrophy was confirmed by the negligible influence of SIKE on cardiac remodelling when SIKE lost its binding capacity to TBK1. Considering the established SIKE–TBK1 interaction, the direct engagement of TBK1 in AKT signalling^19^,^28^,^29^, and the essential role of AKT in SIKE-regulated cardioprotection, SIKE most likely responds to pro-hypertrophic stimuli and interacts with TBK1 to inhibit the downstream AKT-GSK3β/mTOR pathway, thereby affecting gene transcription and protein translation and suppressing the development of cardiac hypertrophic pathologies.

Marion *et al*. recently reported that SIKE is a substrate of TBK1 and is a mixed-type inhibitor of TBK1-regulated IRF3 phosphorylation, based on *in vitro* kinetic analyses. The SIKE–TBK1 interaction is modulated by SIKE phosphorylation at Ser-185 (ref. 18). However, the levels of phosphorylated Sike were not significantly altered during cardiac remodelling, nor were they significantly affected by Tbk1. The discrepancy regarding the effect of Tbk1 on Sike between the present study and the previous studies by Marion *et al*. might be attributed to the different reaction systems. Marion *et al*. used an *in vitro* Michaelis–Menten kinetic assay reaction system and HEK293 cells, whereas our present study employed primary cardiomyocytes *in vitro* and an *in vivo* mouse cardiac hypertrophic model. In addition, previous studies have also reported that TBK1 serves as a key convergence point in multiple immune signalling pathways, including the activation of TRAF3 cascades, which have been shown to be involved in the initiation and progression of pathological cardiac hypertrophy^18^,^19^. However, upregulation of Sike showed a negligible influence on the binding capacity of Tbk1 to Traf3, either with or without the challenge of Ang II. Although we cannot exclude the possibility that SIKE could regulate the activation of TBK1 through other hypertrophic regulators, we clearly demonstrate that SIKE mediates the progression of cardiac remodelling through its direct interaction with TBK1.

Intriguingly, although SIKE showed a significant influence on pathological cardiac hypertrophy induced pharmacologically or by pressure overload, artificial overexpression of or deficiency in Sike did not result in abnormalities under basal conditions in mice or rats. In addition, the physiological cardiac hypertrophic response was not affected by Sike deficiency, despite Sike's role in the regulation of Akt activation under pathological conditions, indicating that Sike might serve as a promising target for the prevention of cardiac hypertrophy under pathological cardiac conditions. In this regard, previous gene-based therapies in mice have attracted substantial attention^11^,^12^,^30^. Here, we further evaluated the role of SIKE in large animal models because of species variation, and we validated the potential gene therapeutic value of SIKE in protecting against cardiac remodelling in rats and monkeys. Importantly, simultaneously decreased SIKE expression was identified in the hearts of human HCM and DCM patients, accompanied by increased activation of the TBK1/AKT axis, compared with normal controls. These findings positively suggest the potential clinical translation of SIKE into a therapeutic target for pathological cardiac remodelling.

In summary, this study identifies SIKE as a critical negative regulator of pathological cardiac hypertrophy in rodents and primates, via its direct inhibition of the TBK1/AKT signalling axis. Although further investigation is warranted, the application of SIKE-induced cardioprotection was validated in rodents and in a large-animal monkey cardiac disease model, thereby emphasizing the translational potential of SIKE as a gene-based therapeutic target for pathological cardiac hypertrophy and heart failure.

## Methods

### Human heart tissues

All of the studies that involved human samples conformed with the Declaration of Helsinki and were approved by the Human Research Ethics Committees of Renmin Hospital of Wuhan University in Wuhan, China. Written informed consent was obtained from the families of the prospective heart donors before heart sample collection. Explanted failing hearts were obtained from patients undergoing cardiac transplantation for end-stage cardiac heart failure secondary to idiopathic DCM and HCM^10^,^31^. Normal LV tissues were obtained from prospective multi-organ donors who had died of head trauma or intracranial bleeding; these hearts were unsuitable for transplantation for technical reasons. All of the tissues were stored at −80 °C or were fixed in 10% formalin, followed by embedding in paraffin for further analyses.

### Mouse studies

All of the experiments that involved animals were approved by the Animal Care and Use Committee of Renmin Hospital of Wuhan University and were conducted in accordance with the National Institutes of Health Guide for the Care and Use of Laboratory Animals.

Generation of *Sike*-CKO mice. The targeting construct for the generation of *Sike*-CKO mice was prepared using a 129/Sv genomic BAC clone (clone no. 363F24) that harboured the complete *Sike* locus (2005, 86:753-758), and it was subcloned into a **PL451** vector (2003, 13:476-484). The construct comprised a 3.1-kb 5′ homology arm, a **PGK-EM7-neo** selection cassette for positive selection by G418 and a 1.4-kb 3′ homology arm, as depicted in Supplementary Fig. 1a. Two LoxP sites flanked a sequence that contained the knockout exons (E1 and E2) and the **PGK-EM7-neo** cassette, whereas the **PGK-EM7-neo** cassette alone was additionally flanked by 2 flippase recognition target (FRT) sites. Linearized targeting vectors were electroporated into the V6.5 ES cell line, which was derived from C57BL/6 and 129S6SvEv F1 hybrid mice. Colonies resistant to G418 were selected and expanded for screening. Genomic DNA from resistant ES cell clones was prepared and screened for positive ES clones via long-range PCR using Platinum Taq DNA polymerase (Invitrogen), using the primers *Sike*-F1 and *Sike*-R1. The forward primer is located within the neo cassette, and the reverse primer is located outside the 3′ homology arm. In addition, the primer pair *Sike*-F3 and *Sike*-R3 was used to confirm the existence of the left LoxP site in the positive clones. The FRT sites that flanked the neo cassette were subsequently removed through Flp-mediated recombination. Two heterozygous ES clones that had undergone homologous recombination were then injected into C57BL/6J blastocysts, and the resulting chimeric male mice were mated with C57BL/6J female mice to obtain homozygous *Sike*^*flox/flox*^ (*Sike*-flox) mice. Transmission of the targeted loci was confirmed via PCR of mouse tail DNA using the primers *Sike*-F2 and *Sike*-R2. The tamoxifen-inducible TG mouse Tg (Myh6-cre/Esr1*)1Jmk/J that expressed MerCreMer, driven by the cardiomyocyte-specific *α-MHC* promoter, was obtained from the Jackson Laboratory (α-MHC-MCM, stock No. 005650). Progeny that expressed the *Sike* floxed allele were bred with α-MHC-MCM transgenic mice to generate *Sike*^*flox/flox*^/α-MHC-MCM mice. For the cardiac-specific knockout of Sike, 6-week-old *Sike*^*flox/flox*^/α-MHC-MCM mice were subsequently injected with tamoxifen (25 mg kg^−1^ per day, Sigma, T-5648) for 5 consecutive days to induce Cre recombinase expression. The primers used to genotype the conditional cardiac-specific *Sike* knockout mice are provided in Supplementary Table 2.

Production of tissue-specific *Sike*-TG and Tbk1 binding-defective mutant Sike transgenic (*Sike*-M-TG) mice. Transgenic mice (C57BL/6 background) with cardiac-specific Sike or mutant Sike expression were generated by subcloning the full-length mouse *Sike* cDNA (3593790; Open Biosystems) or mutant *Sike* (Δ71 amino acids (aa)–207 aa) downstream of the cardiac *α-MHC* promoter. The linearized α-MHC-Sike or α-MHC-Sike-M plasmid was microinjected into mouse oocytes that were subsequently introduced into pseudo-pregnant females to obtain the desired transgenic mice. The transgenic mice were identified by PCR analyses of tail genomic DNA using the forward PCR primer 5′-ATCTCCCCCATAAGAGTTTGAGTC-3′ and the reverse PCR primer 5′-GAACAGCTCTCCGCATCACT-3′.

Generation of cardiac-specific Tbk1 conditional knockout (*Tbk1*-CKO) mice and Sike/Tbk1 DKO mice: To create the Tbk1 conditional targeting constructs for homologous recombination, three successive subclonings were conducted. The 5′ homology arm, consisting of a 2.0-kb fragment immediately 5′ of the knockout exon, a DNA fragment containing the knockout exon 1 and a 5′ LoxP site and the 3′ homology arm, consisting of a 1.1-kb fragment immediately 3′ of the knockout exon, were amplified from a 129/Sv genomic BAC clone (clone no. 366K7) harbouring the complete *Tbk1* locus (2005,86:753-758) and were subcloned into the PL451 vector (2003,13:476-484). In the Tbk1 conditional targeting construct, the knockout exon (E1) and **PGK-EM7-neo** cassette were flanked by two LoxP sites, whereas the **PGK-EM7-neo** cassette alone was additionally flanked by two FRT sites, as depicted in Supplementary Fig. 6a. Linearized targeting vectors were electroporated into an ES cell line derived from C57BL/6 and 129S6SvEv F1 hybrid mice. Colonies resistant to G418 were selected and expanded for screening. Genomic DNA from resistant ES cell clones was prepared and screened for positive ES clones via long-range PCR using Platinum Taq DNA polymerase (Invitrogen), using the primers *Tbk1*-F1 and *Tbk1*-R1. The forward primer is located within the Neo cassette and the reverse primer is located 3′ outside the 3′ homology arm. In addition, the primer pair *Tbk1*-F3 and *Tbk1*-R3 was used to confirmed the existence of the left LoxP site in the positive clones. The FRT sites that flanked the neo cassette were subsequently removed through Flp-mediated recombination. Next, two heterozygous ES clones that had undergone homologous recombination were injected into C57BL/6J blastocysts, and the resulting chimeric male mice were mated with C57BL/6J female mice to obtain homozygous *Tbk1*^*flox/flox*^ mice. Transmission of the targeted loci was confirmed via PCR of mouse tail DNA using the primers *Tbk1*-F2 and *Tbk1*-R2. Progeny with the *Tbk1* floxed allele were bred with α-MHC-MCM transgenic mice to generate *Tbk1*^*flox/flox*^/α-MHC-MCM mice. Six-week-old *Tbk1*^*flox/flox*^/α-MHC-MCM mice were then injected with tamoxifen (25 mg kg^−1^ per day, Sigma, T-5648) for 5 consecutive days for the cardiac-specific knockout of Tbk1. The *Tbk1*^*flox/flox*^ mice were crossed with the *Sike*^*flox/flox*^ mice to produce *Sike*^*flox/flox*^/*Tbk1*^*flox/flox*^ mice, which were then bred with α-MHC-MCM mice to generate *Sike*^*flox/flox*^/*Tbk1*^*flox/flox*^/α-MHC-MCM mice. Six-week-old *Sike*^*flox/flox*^/*Tbk1*^*flox/flox*^/α-MHC-MCM mice were then injected with tamoxifen (25 mg kg^−1^ per day, Sigma, T-5648) for 5 consecutive days for cardiac-specific double knockout of Sike and Tbk1 (DKO). The primers used to genotype the conditional cardiac-specific *Tbk1* knockout mice are provided in Supplementary Table 3.

Production of tissue-specific *Tbk1*-TG mice and Sike/Tbk1 DTG mice. To generate *Tbk1*-TG mice, full-length mouse *Tbk1* cDNA (40111752; Open Biosystems) was cloned downstream of the cardiac *α-MHC* promoter. The linearized cardiac-specific plasmid was microinjected into fertilized mouse embryos to produce cardiac-specific *Tbk1*-TG mice that were identified via PCR analysis of tail genomic DNA. The following PCR primers were used: forward 5′-ATCTCCCCCATAAGAGTTTGAGTC-3′and reverse 5′-TGGCTGCATGGTAGAATGTC-3′. *Sike*-TG mice were crossed with *Tbk1*-TG mice to breed DTG mice.

Male mice and their wild-type littermates, aged 8–10 weeks (24–27 g), were used for all of the subsequent experiments.

### Generation of *Sike*-knockout rats

All experiments that involved rats adhered to the experimental animal ethical approval granted by the Institutional Animal Care and Use Committee of Animal Experiment Center/Animal Biosafety Level-III Laboratory of Wuhan University and were conducted in accordance with the National Institutes of Health Guide for the Care and Use of Laboratory Animals.

A TALEN targeting exon 1 (E1) of the *Sike* gene was designed using an online target designer (https://tale-nt.cac.cornell.edu/node/add/talen-old). Repeat variable di-residue arrays that contained HD, NG, NI and NN monomers were assembled following the ‘Unit Assembly' method as previously described^32^, and they were subsequently used to construct a modified TALEN expression vector with a T7 promoter for *in vitro* RNA production. The TALEN expression plasmids were linearized with *Pme*I (NEB, R0560 L) and were purified with phenol/chloroform to generate an RNase-free DNA template for *in vitro* transcription. Mature mRNA was subsequently transcribed and tailed using the mMessage mMachine T7 Ultra Kit (Ambion, AM1345) and was purified using the RNeasy Mini Kit (Qiagen, 74104) following the manufacturer's instructions. These purified mRNAs were mixed with injection buffer (10 mM Tris-HCl, 0.1 mM EDTA, pH 7.4) to a final concentration of 10 ng μl^−1^ mRNA per TALEN arm. A 2-pl aliquot of the mixture was injected into the cytoplasm of SD rat one cell-stage embryos through the FemtoJet 5247 microinjection system under standard conditions. The injected zygotes were transferred into a pseudo-pregnant female rat, and viable pups were obtained. A fragment of the *Sike* gene that spanned the TALEN target site was amplified via PCR using the following primers: forward 5′-CCACCCTAGAAAGGCAGTGG-3′ and reverse 5′-GACCTGCTCCGGCAACAC-3′. The PCR products were purified and then used as templates for direct sequencing to enable identification of editing events. In addition, the PCR products of the founders were cloned into the plasmid **pMD-19T**, and the individual plasmids were sequenced, which enabled the heterozygous and mosaic editing events to be independently analysed. For F1 and F2, the offspring were genotyped using the following primers: *Sike*-172-forward 5′-TTCGGCCACTATGAGCTGTA-3′ and *Sike*-172-reverse 5′-GACCTGCTCCGGCAACAC-3. The PCR products were then resolved by 3.0% agarose gel electrophoresis. Male rats homozygous for Sike deficiency that weighed 200–250 g at 40 days of age were used for the subsequent experiments.

### Monkey husbandry and lentivirus transfection

The cynomolgus monkeys (*Macaca fascicularis*) were purchased from the Hainan Primate Laboratory Animal Developing Company. The research protocols abided by the legal and regulatory requirements of the People's Republic of China and were approved by the Forestry Department of Hubei Province in China. All monkey experiments complied with the experimental animal ethical review granted by the Institutional Animal Care and Use Committee of Animal Experiment Center/Animal Biosafety Level-III Laboratory of Wuhan University. All of the cynomolgus monkeys were male, weighed 3–5 kg and were 4–5 years old. The health of each monkey was determined by the local veterinary department. The monkeys were housed in individual cages with sufficient space for 3 months to acclimate them to the environment following transportation to the animal facility. All of the monkeys were fed special chow designed for non-human primates, as well as seasonal fruits and water *ad libitum*. The room light cycle was 12 h dark/12 h light, and the lights were set on an automatic timer. The room temperature was 22–26 °C. The monkeys were randomly assigned to the Lenti-*SIKE*-treated experimental group (*n*=10) or the Lenti-Vector-treated control group (*n*=11).

To achieve SIKE overexpression, the monkeys were subjected to lentiviral intracoronary transfection. First, the inferior vena cava was cross-clamped to reduce the venous drainage and intracardiac pressure. The ascending aorta was subsequently cross-clamped, and lentivirus (10^9^ transfection units) in 1 ml of medium was injected into the aortic root proximal to the aortic clamp through a 25-G needle. The aorta was clamped for additional 30 s to enable virus transfection through the coronary circulation; all of the clamps were then released. Before chest closure, another 1 ml of lentivirus was sprayed pericardially. The monkeys in the experimental group were transfected with lentivirus that carried the *SIKE*-expressing vector, whereas the control group received only the lentiviral vector. Rational of optimal lentivirus dose and transfection route for *in vivo* cardiac transfection were obtained from preliminary experiments performed on miniature pigs.

### Lentivirus construction

A lentivirus carrying the *SIKE*-coding gene (Lenti-*SIKE*) and *GFP*-coding vector (Lenti-Vector) was constructed. Briefly, full-length *SIKE* cDNA was inserted into the lentiviral vector construct and driven by the cytomegalovirus promoter. Lentiviral particles were produced by co-transfection of the lentiviral vector construct, packaging construct and envelope plasmid into 293T cells. At 48 h after transfection, supernatants were harvested and viral particles were concentrated by ultracentrifugation. Virus stocks were resuspended in serum-free culture medium and stored at −80 °C until use. Virus titres of transfection units were assayed by transfecting 293T cells for 48 h with serial dilutions of concentrated lentivirus and counting green fluorescent protein (GFP)-positive cells under fluorescence microscopy.

### Aortic banding

AB was performed to establish a mouse model of pressure overload-induced cardiac hypertrophy, in accordance with previously described methods^33^,^34^. Briefly, following anaesthetization with pentobarbital sodium (50 mg kg^−1^, Sigma) through an intraperitoneal injection, the left chest of each mouse was opened to identify the thoracic aorta via blunting dissection at the second intercostal space. We subsequently performed AB using a 7-0 silk suture to band the thoracic aorta against a 27-G (for BWs of 24–25 g) or 26-G (for BWs of 26–27 g) needle, followed by needle removal and closure of the thoracic cavity. In addition, Doppler analysis was used to determine whether adequate constriction of the aorta had been produced. A sham-operated group underwent a similar procedure without aortic constriction.

The pressure overload-induced cardiac hypertrophy model in rats was conducted as previously described^35^,^36^. In brief, rats (40 days old and 200–250 g in weight) were anaesthetized with chloral hydrate (300 mg kg^−1^, Sigma) and then were subjected to a midline abdominal incision to expose the abdominal aorta. The abdominal aorta was banded against a 22-G needle with a 7-0 silk suture. The needle was subsequently removed before abdominal closure. In addition, Doppler analysis was used to confirm adequate constriction of the aortas. Similar procedures without aortic constriction were conducted in the sham group rats.

For AB in monkeys, the monkeys were fasted and deprived of water for 12 and 4 h before surgery, respectively. Anaesthesia was induced with 50 mg of ketamine hydrochloride through intramuscular injection, and the monkeys were then positioned on an operating table in the supine position. Atropine (0.05 mg kg^−1^) was injected intramuscularly to prevent excessive airway secretions. Blood pressure and electrocardiograms were monitored with a Dash 2000 Patient Monitor (GE Healthcare). General anaesthesia suitable for a median thoracotomy was administered via a bolus intravenous injection of ketamine hydrochloride (15 mg kg^−1^) and midazolam (1.5 mg kg^−1^). The monkeys were subsequently intubated and ventilated with a tidal volume of 10 ml kg^−1^ at 40 breaths per minute. A median thoracotomy was performed, and the epicardium was suspended to expose the heart of each monkey. The ascending aorta was bluntly mobilized from the surrounding connective tissues, and lentivirus intracoronary transfection was performed as previously described. When haemodynamic stability was achieved following virus transfection, aortic constriction surgery was performed and 2-0 silk was used to ligate the ascending aorta against a bent iron stick (3.2 mm in diameter for 3–4 kg monkeys or 4 mm for 4–5 kg monkeys). After knotting, the stick was promptly removed, and proper constriction was confirmed by Doppler analysis. Aortic constriction performed with our method produced ∼80% narrowing of the ascending aorta. The sternum, muscle and skin were subsequently closed in three layers. The total operation time from intubation to skin closure was less than 40 min. The ventilator was removed when each monkey was able to breathe spontaneously. The monkeys were intramuscularly injected with sufentanil (5 μg kg^−1^) to alleviate pain associated with the operation and then were returned to the cages when fully resuscitated. To prevent infection, 1.2 million IU benzathine benzylpenicillin was injected intramuscularly. All of the monkeys in the experimental group survived the surgery until they were killed for further experiments; however, one of the monkeys in the control group died after AB surgery, thus rendering the mortality of the control group at ∼10%. Seventy days following surgery, the monkeys were deeply sedated and killed via 10% KCl intravenous injection. The heart tissue of each monkey was collected for further analysis.

### Exercise protocols

To induce physiological cardiac hypertrophy and remodelling, the mice were subjected to swimming training in accordance to the protocol described^37^. In brief, during the first 8 days, forced swimming was performed in 8- to 10-week-old mice for 10 min twice per day, with an increment of 10 min each day until two sessions of 90 min were achieved on the 9 day. Thereafter, all training mice swam for 14 additional days (22 days total) by two daily swimming sessions of 90 min. During swimming, the mice were continuously monitored to avoid submerging under the water surface and to ensure equal exertion. On the 23 day, mice were killed for further analyses.

### Ang II infusion

Male mice and their wild-type littermates, aged 8–10 weeks (24–27 g) were subjected to Ang II infusion to establish a mouse model of cardiac hypertrophy. Ang II (1.4 mg kg^−1^ per day and dissolved in 0.9% NaCl) was subcutaneously infused for 4 weeks using an osmotic minipump (Alzet model 2004; Alza Corp) implanted in each mouse (*n*=10–12 mice for each group). Saline-infused mice that were subjected to the same procedures as the experimental group but without Ang II infusion, served as infusion controls. The protocol for Ang II infusion-induced cardiac hypertrophy was modified from ref. 38.

### Echocardiography measurements

Echocardiography measurements were performed at the indicated times to evaluate the cardiac function of the mice^10^. First, the surviving mice were anaesthetized using 1.5–2% inhaled isoflurane. For echocardiography measurements, M-mode tracings derived from the short axis at the papillary muscle level and the parasternal long axis of the LV were recorded using a Mylab30CV ultrasound system (Biosound Esaote Inc.) equipped with a 15-MHz probe. LV end-diastolic dimension (LVEDd) and LV end-systolic dimension (LVESd) were measured at the time of the largest and smallest LV areas, respectively. Echocardiographic measurements were performed in triplicate and then were averaged. LV fractional shortening (LVFS) was calculated using the following formula: LVFS (%)=(LVEDd-LVESd)/LVEDd × 100%.

Echocardiography measurements in monkeys were performed by a sonographer at the indicated times on a Vivi7 Ultrasonic Doppler System (GE Healthcare) equipped with an 8-MHz 10 S probe (GE Healthcare). M-mode pictures captured from the left parasternal long axis view were recorded to measure the interventricular septal thickness at end diastole, interventricular septal thickness at end systole, left ventricular posterior wall thickness at end diastole and left ventricular posterior wall thickness at end systole.

### Histological analysis

Four weeks after the AB or sham surgery, the mice and rats were killed to determine the parameters of hypertrophic growth and cardiac fibrosis. The hearts of the mice and rats were excised, arrested in diastole with a 10% potassium chloride solution and then fixed in 10% formalin, followed by embedding in paraffin according to standard histological protocols. The hearts were then sectioned transversely at 5 μm, in the region close to the apex to visualize the left and right ventricles. The sections were stained with H&E and PSR to evaluate histopathology and collagen deposition, respectively. Myocyte cross-sectional areas were detected via fluorescein isothiocyanate-conjugated WGA (Invitrogen) staining. 4,6-Diamidino-2-phenylindole (DAPI) was used to label the nuclei. At least 100 circular to oval-shaped LV myocytes from more than six different mice or rats per group were traced. Fibrosis was measured as a positively stained area with PSR and was expressed as a percentage of the total area. More than 40 fields per group were calculated. The myocyte cross-sectional and fibrotic areas were measured using Image-Pro Plus software (version 6.0) with captured images.

### Immunohistochemical analysis

For immunohistochemistry staining, paraffin-embedded hearts were cut transversely into 5-μm sections. Following a 5-min high-pressure antigen retrieval process in citrate buffer with a pH of 6.0, the heart sections were blocked with 10% bovine serum albumin for 60 min and were subsequently incubated overnight at 4 °C with the primary antibodies. Binding was visualized with the appropriate peroxidase-conjugated secondary antibodies (Horseradish Peroxidase AffiniPure Goat Anti-Rabbit IgG (H+L), A21020, Abbkine, 1:200) for 60 min at 37 °C.

### Cardiomyocyte culture and recombinant adenoviral vector infection

Primary NRCMs were prepared from the hearts of 1- to 2-day-old SD rats, as previously described. Briefly, PBS containing 0.03% trypsin and 0.04% collagenase type II was used to isolate cardiomyocytes, followed by fibroblast removal using a differential attachment technique. The NRCMs were seeded at a density of 2 × 10^5^ cells per well onto six-well culture plates coated with gelatin in plating medium, which consisted of DMEM/F12 supplemented with 20% fetal calf serum, 5-bromodeoxyuridine (0.1 mM, to inhibit fibroblast proliferation) and penicillin/streptomycin. For the assessment of cardiomyocyte hypertrophy, the medium was exchanged with serum-free DMEM/F12, and the NRCMs were subsequently stimulated with Ang II (1 μM) and PE (100 μM) 48 h later. We constructed adenoviruses that carried sequences encoding rat Sike (Ad*Sike*), Tbk1-binding-defective mutant Sike lacking the Tbk1-binding domain (depleted of the Δ71-207 amino acid; Ad*Sike*-M), Tbk1 (Ad*Tbk1*), constitutively active Akt (Ad*caAkt*), dominant negative Akt (Addn*Akt*), short hairpin RNA targeting *Sike* (Adsh*Sike*) and short hairpin RNA targeting *Tbk1* (Adsh*Tbk1*). Similar adenoviral vectors that encoded the GFP gene (Ad*GFP*) and short hairpin RNA (AdshRNA) served as controls. The NRCMs were infected with the corresponding adenoviruses at a multiplicity of infection of 100 particles per cell for 24 h and were used for subsequent experiments.

### Immunofluorescence analysis

The cell surface area of the NRCMs was assessed via immunofluorescence staining. Briefly, the cardiomyocytes were treated with Ang II or PE for 48 h after infection with the corresponding adenoviruses for 24 h. The cells were subsequently fixed with 3.7% formaldehyde, permeabilized with 0.1% Triton X-100 in PBS for 5 min and stained with α-actinin (05-384, Merck Millipore, 1:100 dilution), followed by staining with a fluorescent secondary antibody (Donkey anti-Mouse IgG (H+L) Secondary Antibody, A21202, Invitrogen, 1:200). The surface areas were measured using Image-Pro Plus software, version 6.0.

### Western blot analysis

Total protein was extracted from heart tissues and primary cells in lysis buffer (720 μl of RIPA, 20 μl of phenylmethyl sulphonyl fluoride, 100 μl of complete protease inhibitor cocktail, 100 μl of Phos-stop, 50 μl of NaF and 10 μl of Na_3_VO_4_). Protein concentrations were determined using a Pierce BCA Protein Assay kit. Fifty micrograms of protein were subjected to SDS–polyacrylamide gel electrophoresis (SDS–PAGE; Invitrogen) and were transferred to a polyvinylidene fluoride membrane (Millipore), followed by incubation with the corresponding primary antibodies on a rocking platform at 4 °C overnight. Following incubation with peroxidase-conjugated secondary antibodies (Jackson ImmunoResearch Laboratories, 1:10,000 dilution), the bands were visualized using Bio-Rad ChemiDocTM XRS+ (Bio-Rad). The protein expression levels were normalized to the corresponding GAPDH levels. Antibodies for western blot used in this study are provided in Supplementary Table 4. Full-scan results of western blots are shown in Supplementary Fig. 12.

### Quantitative real-time PCR

Total mRNA was extracted using TRIzol reagent (15596-026, Invitrogen) according to the manufacturer's instructions. mRNA was converted to cDNA using oligo(dT) primers with a Transcriptor First Strand cDNA Synthesis Kit (04896866001, Roche). Quantitative real-time PCR amplification of the indicated genes was performed using SYBR Green (04887352001, Roche). PCR thermal cycling involved a denaturing step at 95 °C for 10 min, followed by 45 cycles of annealing at 95 °C for 10 s, 60 °C for 10 s and 72 °C for 20 s. The target gene expression was normalized to GAPDH gene expression. The primer pairs for real-time PCR used in this study are listed in Supplementary Table 5.

### Plasmid constructs

GST-SIKE and pSicoR-Myc-SIKE were generated by cloning the human SIKE gene into **pGEX-4T-1** and **pSicoR-Myc-C1**, respectively. pSicoR-Flag-TBK1 was constructed by cloning the entire coding region of the human TBK1 gene into the *Eco*RI and *Xho*I sites of the **pSicoR-Flag-C1** plasmid. DNA fragments that encoded the various SIKE (1–70 aa, 71–207 aa, 1-162 aa and 163–207 aa) and TBK1 (1–301 aa, 1–383 aa, 302–729 aa and 384–729 aa) truncates were prepared via PCR and were cloned into the **pSicoR-Myc-C1** and **pSicoR-Flag-C1** expression vectors, respectively. All of the plasmids were verified by sequencing. The primer pairs used in this study are listed in Supplementary Table 6.

### Immunoprecipitation (IP) assay

An IP assay was performed as previously described to determine protein–protein interactions^10^. Cultured HEK293T cells co-transfected with the appropriate plasmids were washed with cold PBS (HyClone) and were lysed with lysis buffer (20 mM Tris-HCl, pH 7.4, 150 mM NaCl, 1 mM EDTA, 1% Triton X-100) supplemented with protease inhibitor cocktail tablets (04693132001, Roche). Following incubation for 20 min at 4 °C and centrifugation at 13,000*g* for 15 min, the cell lysates were pre-cleared with normal mouse or rabbit immunoglobulin G and protein A/G-agarose beads (11719394001 and 11719386001, Roche). The pre-cleared lysates were subsequently incubated with the indicated primary antibodies and protein G-agarose (11243233001, Roche) on a rocking platform at 4 °C overnight. The immunoprecipitated proteins were collected, washed five to six times with lysis buffer, boiled with 2 × SDS loading buffer, separated using SDS–PAGE and then electrophoretically transferred to polyvinylidene difluoride membranes (Millipore). The membranes were blocked with 5% bovine serum albumin in Tris-buffered saline containing 0.1% Tween-20 and were immunoreacted with the indicated primary and secondary antibodies conjugated to horseradish peroxidase. Co-IP for endogenous proteins was performed in the cytoplasmic portion of the NRCMs isolated using an NE-PER nuclear and cytoplasmic extraction kit (Pierce, catalogue number: 78835).

### GST pull-down assay

For the GST pull-down assay, immunopurified Flag-TBK1 was prepared according the IP assay, and then A/G-agarose beads were incubated in elution buffer (50 mM HEPES, pH 7.4, 500 mM NaCl, 1% Triton X-100, 3 μg μl^−1^ FLAG peptide (F4799, Sigma)) for 2 h at 4 °C. The Rosetta (DE3) *Escherichia coli* was transformed with vector pGEX-4T-1-GST-SIKE, and then it was induced with 1 mM isopropyl-β-D-thiogalactopyranoside at an optical density at 600 nm (OD600) of 0.6. *E. coli* extracts were prepared in PBS containing protease inhibitor cocktail tablets (04693132001, Roche), and they were incubated with glutathione-sepharose 4B beads (17075601, GE Healthcare Biosciences AB) for 1 h at 4 °C. Subsequently, the beads loaded with the proteins were washed five times with 1 ml of PBS and were incubated for an additional 4 h at 4 °C with immunopurified Flag-TBK1. The glutathione-sepharose 4B beads then were washed three times with 1 ml of the IP lysis buffer in the absence of cocktail, and the eluted proteins, in buffer containing 20 mM reduced glutathione, were resolved via SDS–PAGE and analysed via western blotting using anti-FLAG antibodies. A GST tag was used as the negative control under the same conditions.

### Confocal microscopy

After co-transfection with pEGFP-SIKE and pmCherry-TBK1, the HEK293T cells were cultured on gelatin-coated coverslips in 24-well plates for 48 h. The cells were fixed with 4% fresh paraformaldehyde for 15 min, followed by three washes with PBS, 5 min of permeabilization with 0.2% Triton X-100 in PBS and subsequent incubation in an Image-IT FX signal enhancer (I36933, Invitrogen) for 30 min. The cells were then washed three times with TBST and stained with DAPI (1 g ml^−1^, 15 min). The slides were mounted with mounting solution (D2522, Sigma), and images were acquired using a confocal laser-scanning microscope (Fluoview 1000; Olympus).

### Statistical analysis

The data are expressed as the mean±standard deviation (s.d.). Differences between two groups were analysed using Student's two-tailed *t*-test, and a one-way analysis of variance was applied for the comparison of multiple groups, followed by the least significant difference (equal variances assumed) or Tamhane's T2 (equal variances not assumed) test. All of the statistical analyses were performed using SPSS (Statistical Package for the Social Sciences) software, version 13.0. Differences were considered significant at *P* values less than 0.05.

## Additional information

**How to cite this article**: Deng, K.-Q. *et al*. Suppressor of IKKɛ is an essential negative regulator of pathological cardiac hypertrophy. *Nat. Commun.* 7:11432 doi: 10.1038/ncomms11432 (2016).

## Supplementary Material

Supplementary Information Supplementary Figures 1-12 and Supplementary Tables 1-6
