# Author Correction: Suppressor of IKKɛ is an essential negative regulator of pathological cardiac hypertrophy

**DOI:** 10.1038/s41467-023-38331-w

**Published:** 2023-05-25

**Authors:** Ke-Qiong Deng, Aibing Wang, Yan-Xiao Ji, Xiao-Jing Zhang, Jing Fang, Yan Zhang, Peng Zhang, Xi Jiang, Lu Gao, Xue-Yong Zhu, Yichao Zhao, Lingchen Gao, Qinglin Yang, Xue-Hai Zhu, Xiang Wei, Jun Pu, Hongliang Li

**Affiliations:** 1 Department of Cardiology, Renmin Hospital of Wuhan University, Wuhan, 430060 China; 2 Animal Experiment Center/Animal Biosafety Level-III Laboratory, Wuhan University, Wuhan, 430060 China; 3 Medical Research Institute, School of Medicine, Wuhan University, Wuhan, 430071 China; 4 College of Veterinary Medicine, Hunan Agricultural University, Changsha, 410128 China; 5 Division of Cardiothoracic and Vascular Surgery, Heart-Lung Transplantation Center, Sino-Swiss Heart-Lung Transplantation Institute, Tongji Hospital, Tongji Medical College, Huazhong University of Science and Technology, Wuhan, 430000 China; 6 Department of Cardiology, Institute of Cardiovascular Disease, Union Hospital, Tongji Medical College, Huazhong University of Science and Technology, Wuhan, 430000 China; 7 Department of Cardiology, Shanghai Renji Hospital, School of Medicine, Shanghai Jiaotong University, Shanghai, 200127 China; 8 Department of Nutrition Sciences, University of Alabama at Birmingham, Birmingham, AL 35294-3360 USA

Correction to: *Nature Communications* 10.1038/ncomms11432, published online 01 June 2016

The original version of this article contained labeling errors in the uncropped western blot scans in Supplementary Fig. 12*,* where the labels of Figures 6c, 6d, 8b–8j, 8k, 9a, 9h and 10c should be 7a, 7b, 10b-10j, 11a, 12a, 12h and 13c, respectively.

Also, In Fig. 11h, the PSR staining in the AB/NTG group and AB/Sike-M TG groups were swapped with each other.

The correct version of Fig. 11 is:


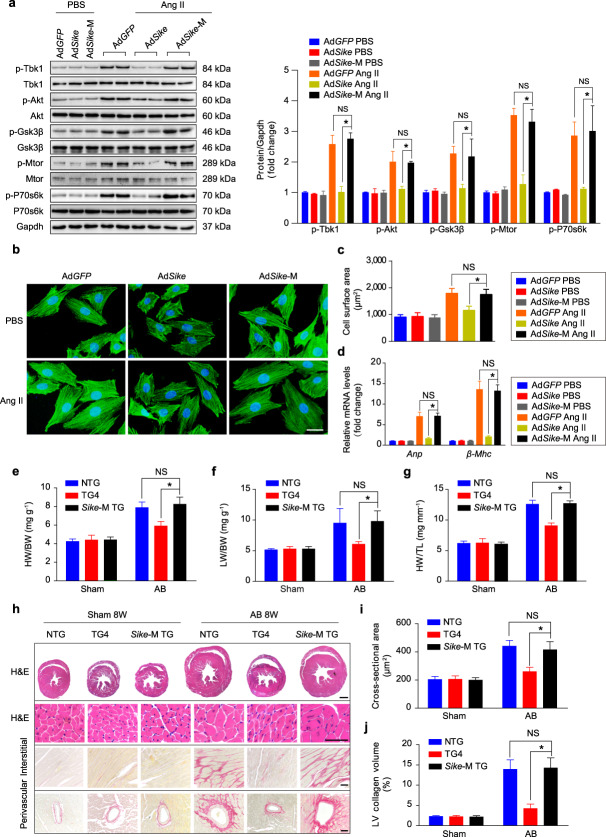


which replaces the incorrect version of Fig. 11:


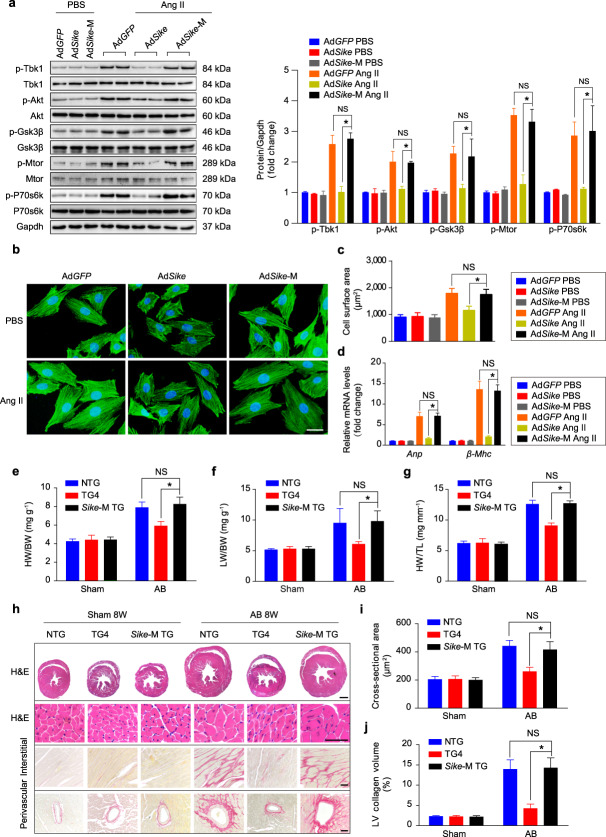


These errors occurred during final proof processing and do not affect any results or conclusions of the article. The HTML has been updated to include a corrected version of the Supplementary Information.

## Supplementary information

Updated Supplementary Information

