# Supplementary material for: Author Correction: Suppressor of IKKɛ is an essential negative regulator of pathological cardiac hypertrophy
Source: Nat Commun. 2023 May 25;14:3018. doi: 10.1038/s41467-023-38331-w (PMC10212913; doi:10.1038/s41467-023-38331-w)
Supplement: Supplementary file 1 — Updated Supplementary Information [file 41467_2023_38331_MOESM1_ESM.pdf]

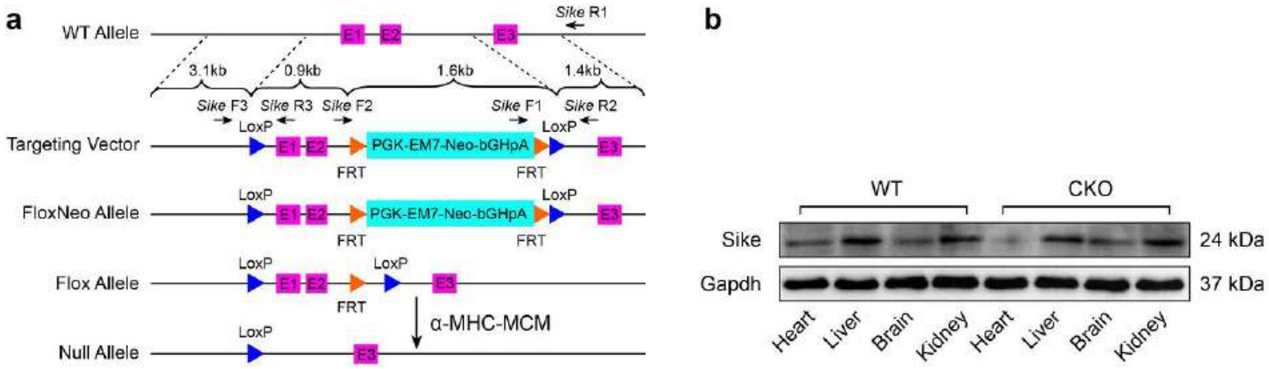

Supplementary Figure. 1 Generation of cardiac-specific Sike knockout (*Sike*-CKO) mice. (a) Schematic demonstrating the strategy for the generation of *Sike*-CKO mice. **(b)** Immunoblotting indicated Sike ablation only in the hearts of the *Sike*-CKO mice.

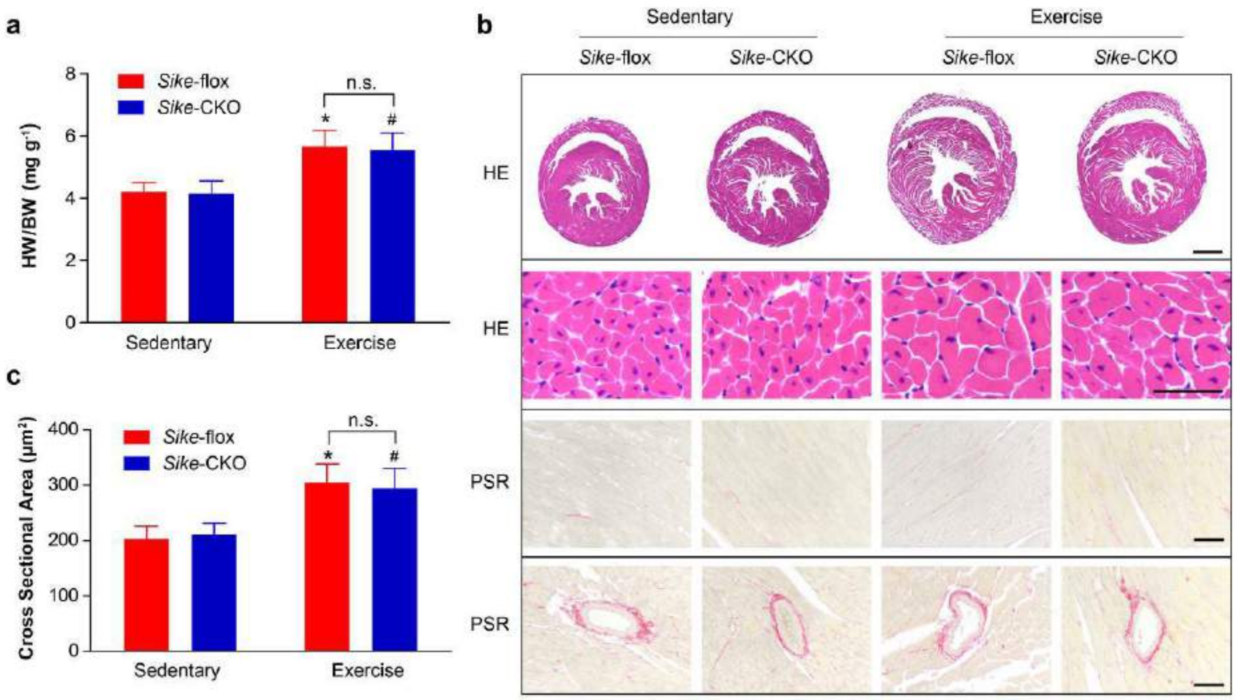

Supplementary Figure. 2 Sike deficiency fails to significantly regulate physiological cardiac hypertrophy. (a) The ratios of HW/BW in the indicated groups (*n*=11-13 mice per experimental group). **(b)** Histological analyses of whole hearts (the first row; scale bar, 1000 μm) and heart sections stained with H&E (the second row; scale bar, 50 μm) or PSR (the third and fourth row; scale bars, 50 μm) from the indicated groups (*n*=6-8 mice per experimental group). **(c)** Statistical results for the cell cross-sectional areas in the indicated groups (*n*≥100 cells per experimental group). **P*<0.05 vs. *Sike*-flox sedentary; #*P*<0.05 vs. *Sike*-CKO sedentary; n.s. indicates no significant difference. Data are presented as the mean±s.d.. Statistical analysis was carried out by one-way ANOVA.

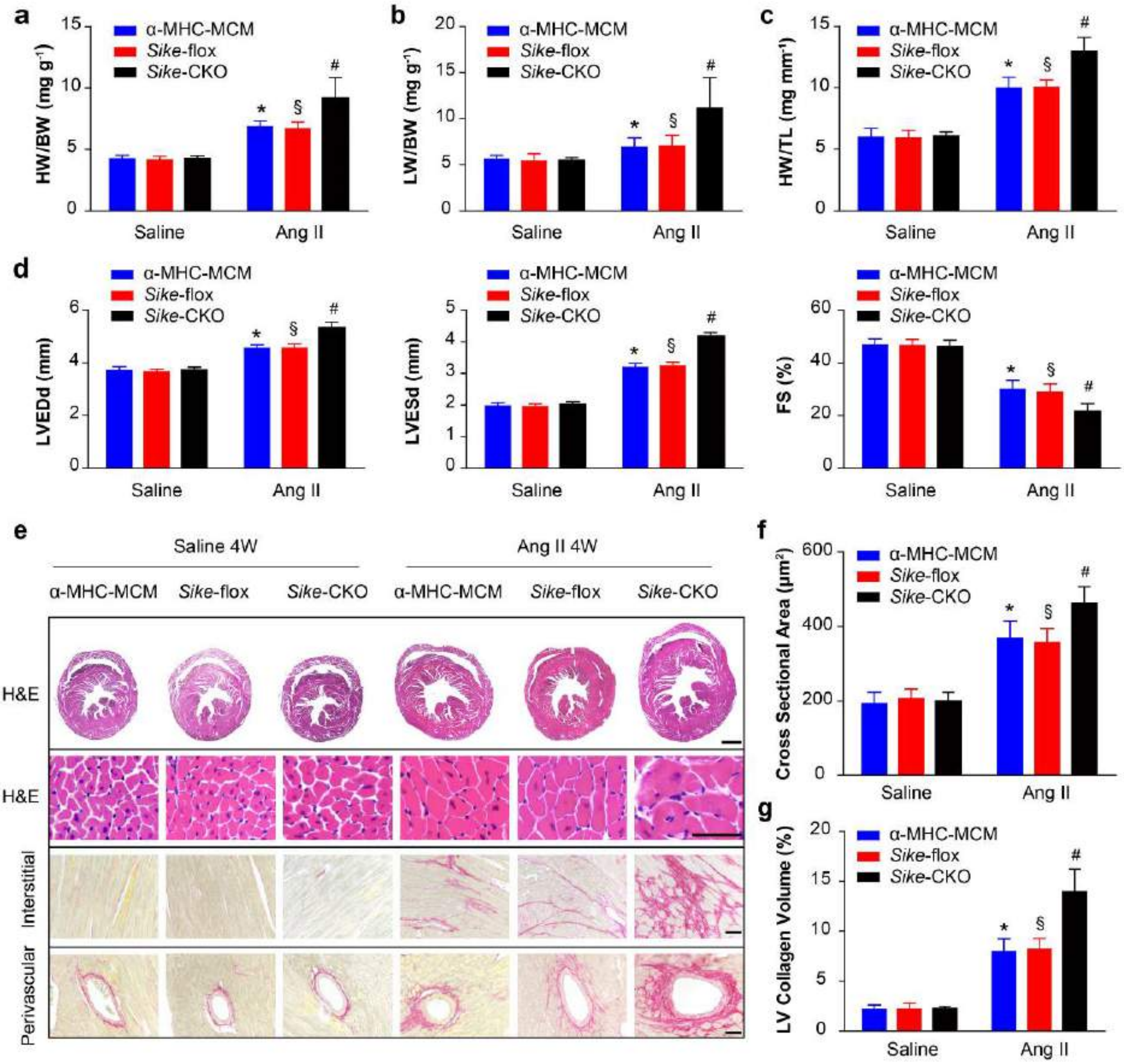

a
b
c
d
e
f
g
α-MHC-MCM
Sike-flox
Sike-CKO
HW/BW (mg g⁻¹)
LW/BW (mg g⁻¹)
HW/TL (mg mm⁻¹)
LVEDd (mm)
LVESd (mm)
FS (%)
Saline
Ang II
Saline 4W
Ang II 4W
H&E
Interstitial
Perivascular
Cross Sectional Area (μm²)
LV Collagen Volume (%)

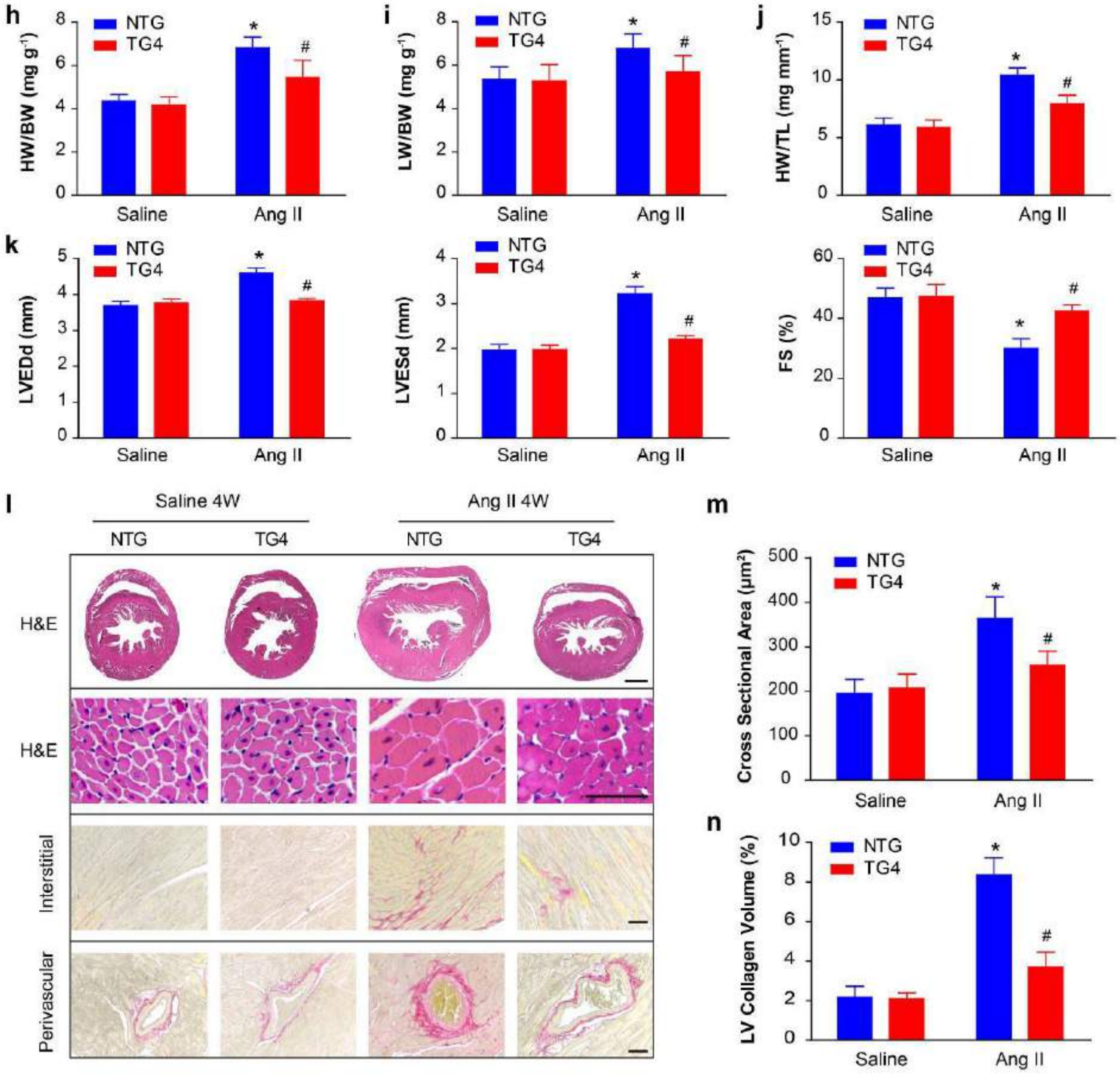

Supplementary Figure. 3 Effects of Sike on agonist-induced cardiac hypertrophy *in vivo*. (a-c) Comparison of the HW/BW **(a)**, LW/BW **(b)** and HW/TL **(c)** ratios in different genotypic mice (α-MHC-MCM, *Sike*-flox and *Sike*-CKO) that received saline or Ang II treatment, *n*=10-12 mice/group. **(d)** Comparison of the echocardiographic parameters in the indicated groups, *n*=9-12 mice/group. **(e)** Histological analyses of whole hearts (the first row; scale bar, 1000 μm) and heart sections from the indicated groups stained with H&E (the second row; scale bar, 50 μm) or PSR (the third and fourth row; scale bars, 50 μm) 4 weeks after the saline or Ang II treatment, *n*=6-8 mice/group. **(f)** Comparison of the cross sectional area of cardiomyocytes from the indicated groups, *n*≥100 cells/group. **(g)** Comparison of the LV collagen volume in the indicated groups, *n*≥40

fields/group. *p<0.05 vs. the saline-treated α-MHC-MCM group; §p<0.05 vs. the saline-treated *Sike*-flox group; #p<0.05 vs. the Ang II-treated α-MHC-MCM or *Sike*-flox group in **a-d** and **f-g**. **(h-j)** Statistical results for the HW/BW **(h)**, LW/BW **(i)** and HW/TL **(j)** ratios in different genotypic mice (NTG and *Sike*-TG4) that received saline or Ang II treatment, *n*=11-12 mice/group. **(k)** Statistical data for the echocardiographic parameters in the indicated groups, *n*=10-12 mice/group. **(l)** Histological analyses of whole hearts (the first row; scale bar, 1000 μm) and heart sections from the indicated groups stained with H&E (the second row; scale bar, 50 μm) or PSR (the third and fourth row; scale bars, 50 μm) 4 weeks after the saline or Ang II treatment, *n*=6-8 mice/group. **(m)** Statistical results for the cross sectional area of cardiomyocytes from the indicated groups, *n*≥100 cells/group. **(n)** Statistical values for the LV collagen volume in the indicated groups, *n*≥40 fields/group.*p<0.05 vs. the saline-treated NTG group; #p<0.05 vs. the Ang II-treated NTG group in **h-k** and **m-n**. Data are presented as the mean±s.d.. Statistical analysis was carried out by one-way ANOVA.

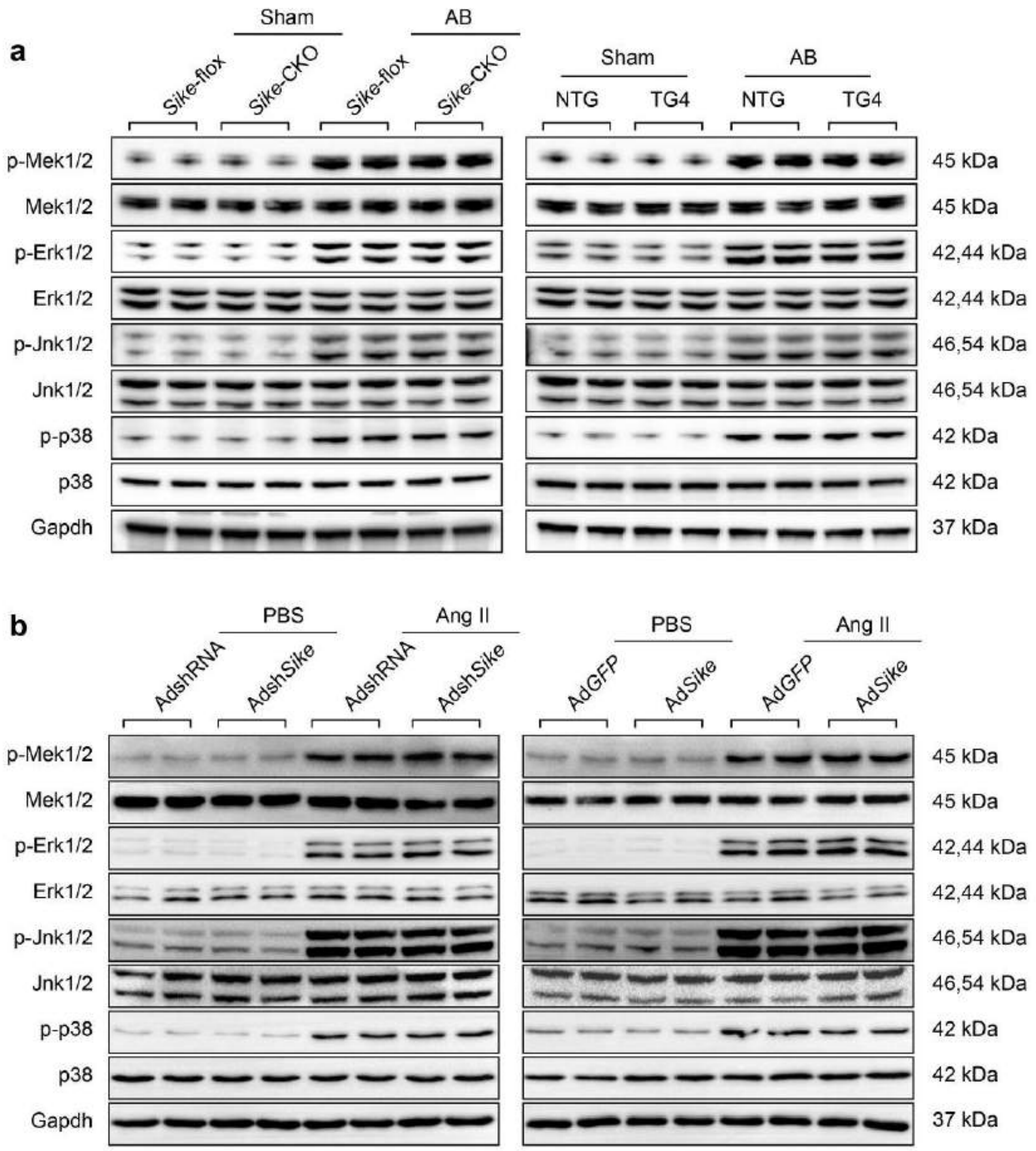

Supplementary Figure. 4 MAPK signaling is not involved in Sike-regulated cardiac hypertrophy. **(a)** Immunoblot of the activities of MAPK signaling components (*e.g.*, Mek1/2, Erk1/2, Jnk1/2 and p38) in the hearts from different *Sike* genotypic mice (*Sike*-flox and *Sike*-CKO, NTG and TG4) subjected to sham or AB surgery (*n*=4 mice per experimental group). **(b)** The activities of MAPK signaling components(*e.g.*, Mek1/2, Erk1/2, Jnk1/2 and p38) in PBS or Ang II-treated NRCMs infected with AdshRNA and Adsh*Sike* **(left)** or Ad*GFP* and Ad*Sike* **(right)** (*n*=4 samples per experimental group).

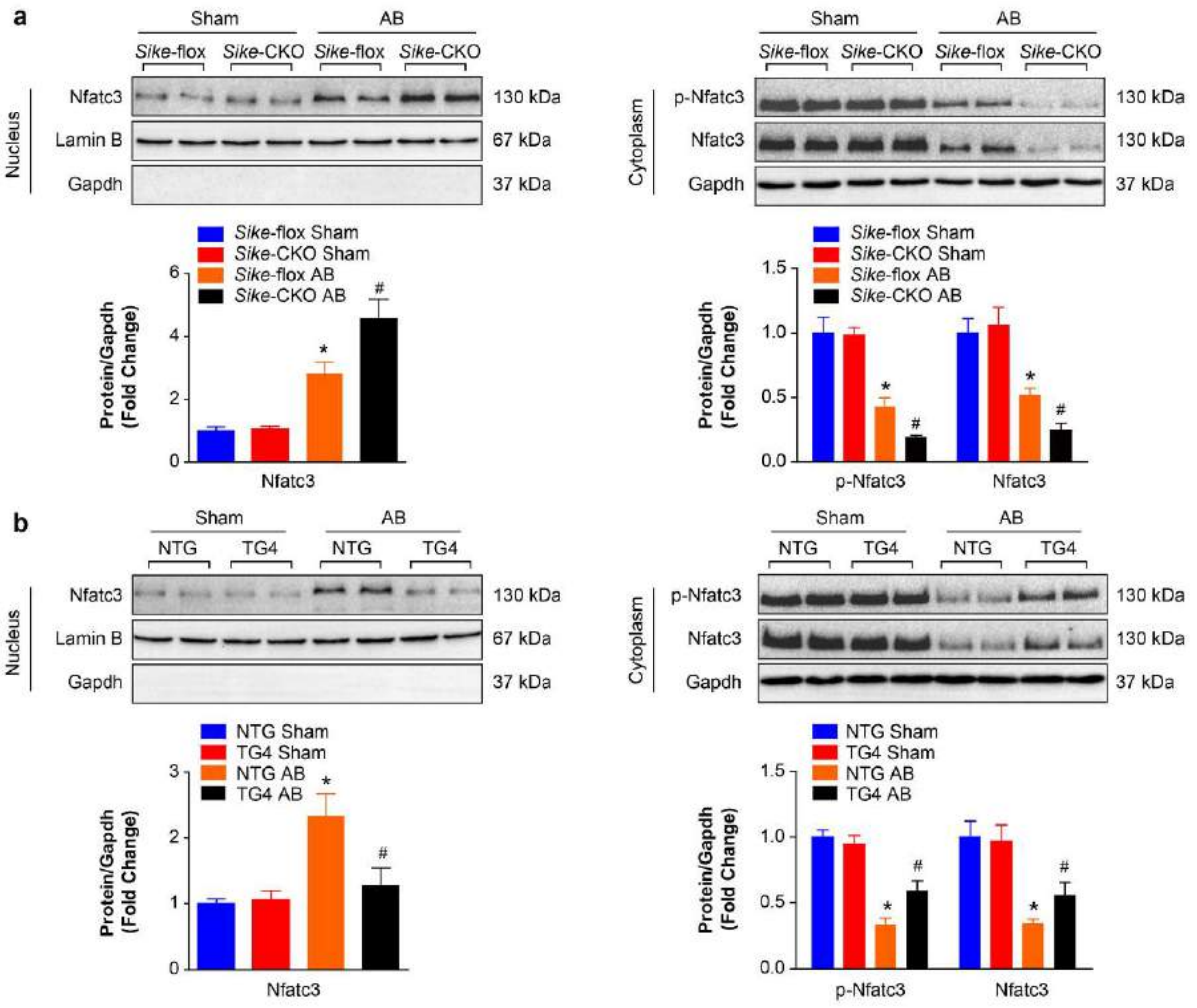

Supplementary Figure. 5 Sike regulates Nfatc3 activity during cardiac hypertrophy. Representative western blots and quantitative results of the phosphorylated and/or total nuclear factor of activated T-cells c3 (Nfatc3) protein levels in the nucleus **(left)** and in the cytoplasm **(right)** of heart samples from *Sike*-CKO (**a**), *Sike*-TG4 (**b**), and their corresponding control mice after sham or AB surgery (n=4 mice per experimental group). *P<0.05 vs. *Sike*-flox sham or NTG sham; #P<0.05 vs. *Sike*-flox AB or NTG AB. Data are presented as the mean±s.d. from at least three independent experiments. Statistical analysis was carried out by one-way ANOVA.

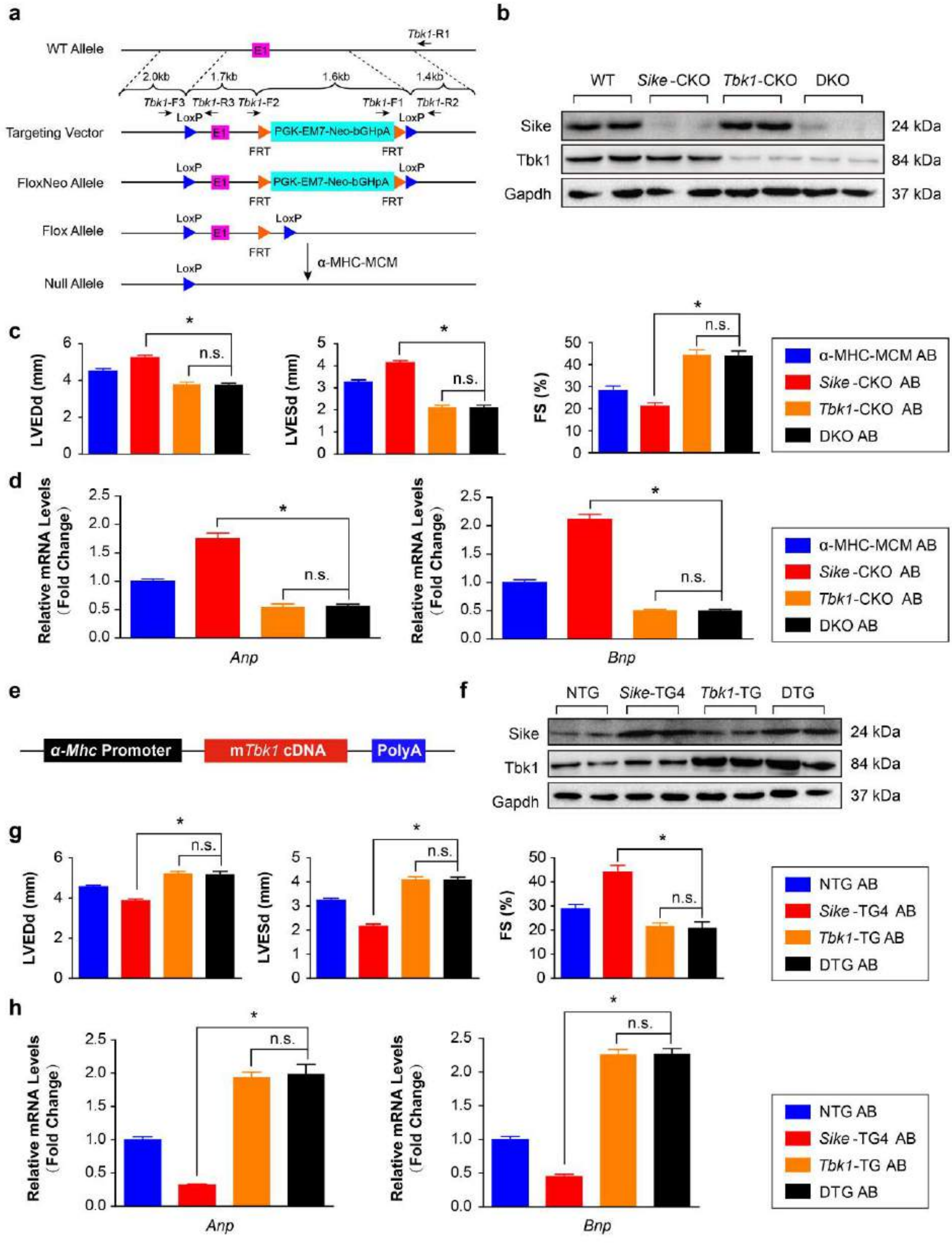

Supplementary Figure. 6 Tbk1 inhibition is required for the negative regulation of Sike on cardiac remodeling. (a) Schematic of the strategy for generating cardiac-specific Tbk1 knockout

(*Tbk1*-CKO) mice. **(b)** The loss of Tbk1 and Sike proteins alone or in combination was confirmed in the hearts of *Sike*-CKO, *Tbk1*-CKO and double knockout (DKO) mice. **(c)** Comparison of the echocardiographic parameters in the indicated groups, *n*=10-12 mice/group. **(d)** mRNA levels of the hypertrophic marker genes (*Anp* and *Bnp*) in the hearts of the indicated groups (*n*=4 mice per experimental group). **(e)** Schematic of *α-Mhc* promoter-driven mouse cDNA of the *Tbk1* transgenic construct. **(f)** Increased expression of the Sike and Tbk1 proteins in the hearts of *Sike*-TG4, *Tbk1*-TG and double transgenic (DTG) mice. **(g)** Comparison of the echocardiographic parameters in the indicated groups, *n*=9-11 mice/group. **(h)** Transcript levels of the hypertrophic marker genes (*Anp* and *Bnp*) in the indicated groups (*n*=4 mice per experimental group). *$p<0.05$ compared between the two indicated groups; n.s. indicates no significance.Data are presented as the mean±s.d. from at least three independent experiments. Statistical analysis was carried out by one-way ANOVA.

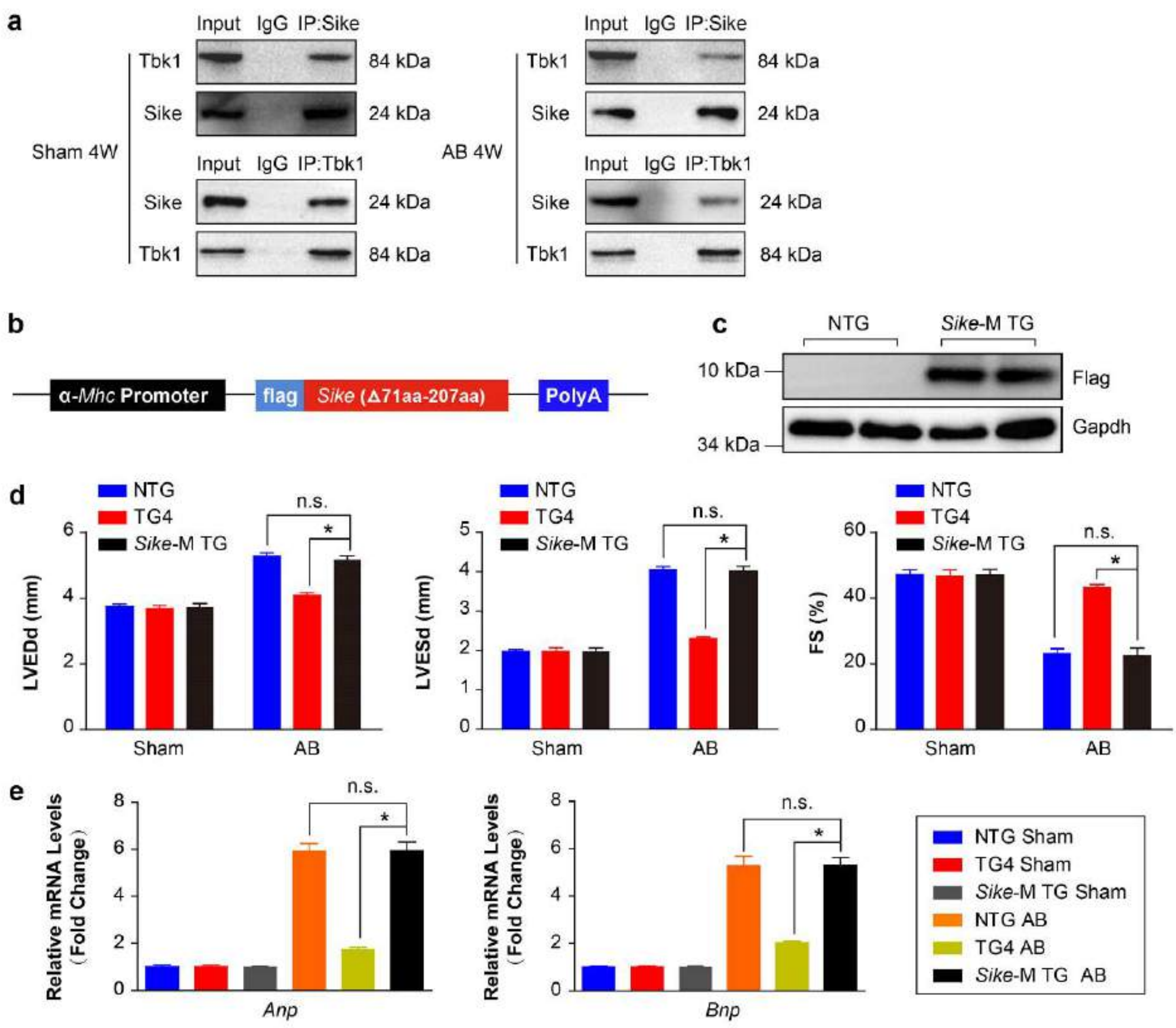

Supplementary Figure. 7 Sike ameliorates pathological cardiac remodeling dependent on Sike-Tbk1 interaction. **(a)** Co-IP assay was performed using SIKE or TBK1 antibody followed by examining the expression levels of Tbk1 or Sike using Western blot under basal condition (**left**) or after 4 weeks of AB treatment (**right**). **(b)** Schematic diagram illustrates the construct used to generate Tbk1 binding-defective mutant Sike transgenic (*Sike*-M TG) mice. **(c)** Cardiac mutant Sike expression was confirmed by Western blot. (**d**) Comparison of the echocardiographic parameters in the indicated groups, *n*=9-11 mice/group. **(e)** mRNA levels of the hypertrophic marker genes (*Anp* and *Bnp*) in the indicated groups (*n*=4 mice per experimental group). Data are presented as the mean±s.d. from at least three independent experiments. Statistical analysis was carried out by one-way ANOVA.

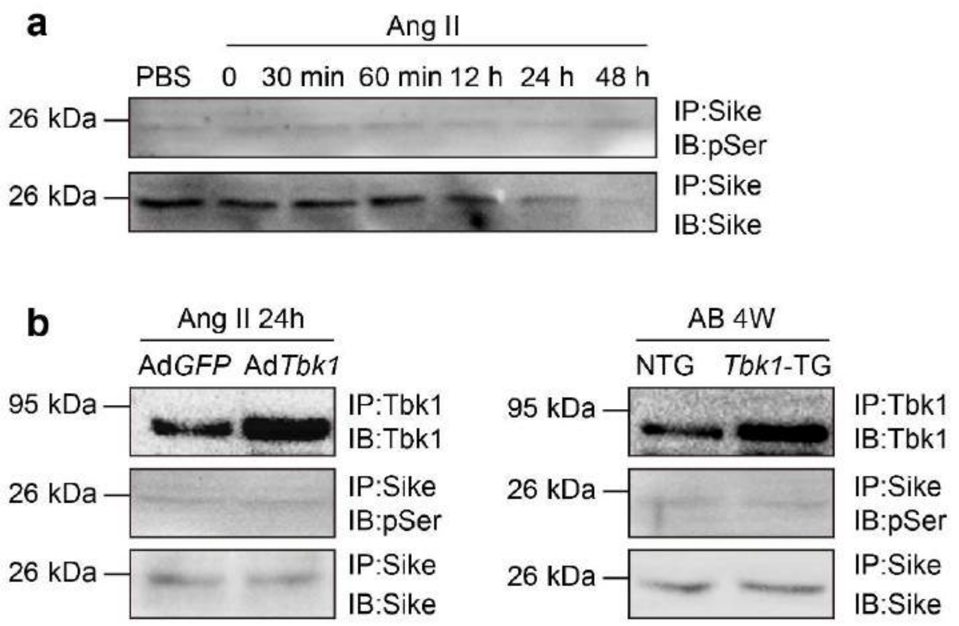

Supplementary Figure. 8 Expression of phosphorylated Sike is not significantly mediated by Tbk1. **(a)** After NRCMs were treated with PBS or Ang II for 0, 30 min, 60 min, 12h, 24h or 48h, Sike was immunoprecipitated (IP) from lysates and the total and phosphorylated Sike was assessed via Western blot using anti-SIKE and anti-phospho-Ser antibody, respectively. **(b)** The protein expression of total and phosphorylated Sike in Ang II-treated NRCMs infected with Ad*GFP* or Ad*Tbk1* and in heart samples from NTG or *Tbk1*-TG mice subjected to AB surgery. Three independent experiments were performed respectively.

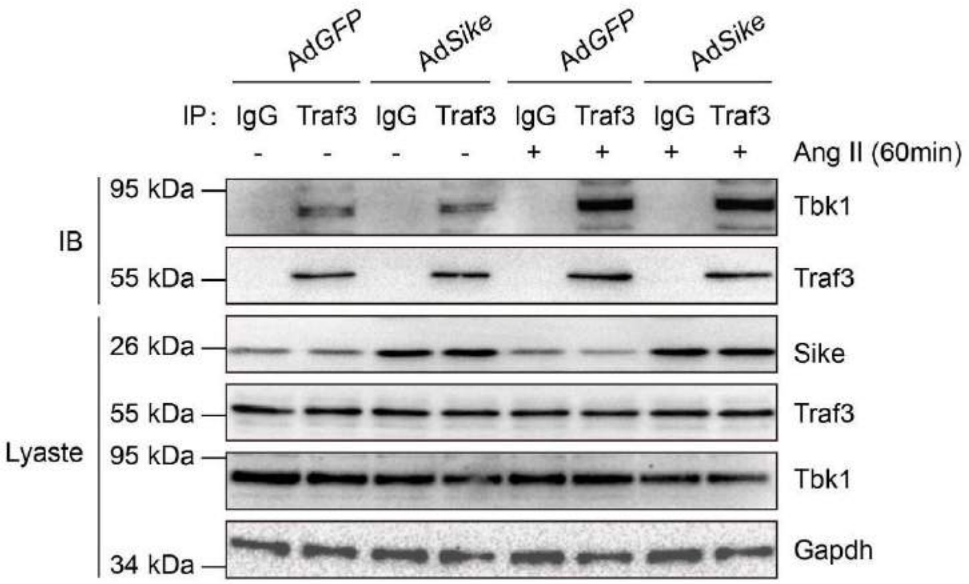

Supplementary Figure. 9 Sike failed to significantly influence Tbk1-Traf3 binding during cardiomyocytes enlargement. The interaction of Tbk1 with Traf3 was examined by Co-IP experiments, which were conducted in primary NRCMs with anti-TRAF3 antibody followed by measuring the Tbk1 expression using Western blotting under basal status or after Ang II stimulation for 60 min. The influence of Sike in Tbk1-Traf3 interaction was examined by transfecting NRCMs with Ad*Sike*. Ad*GFP* was used as a negative control.

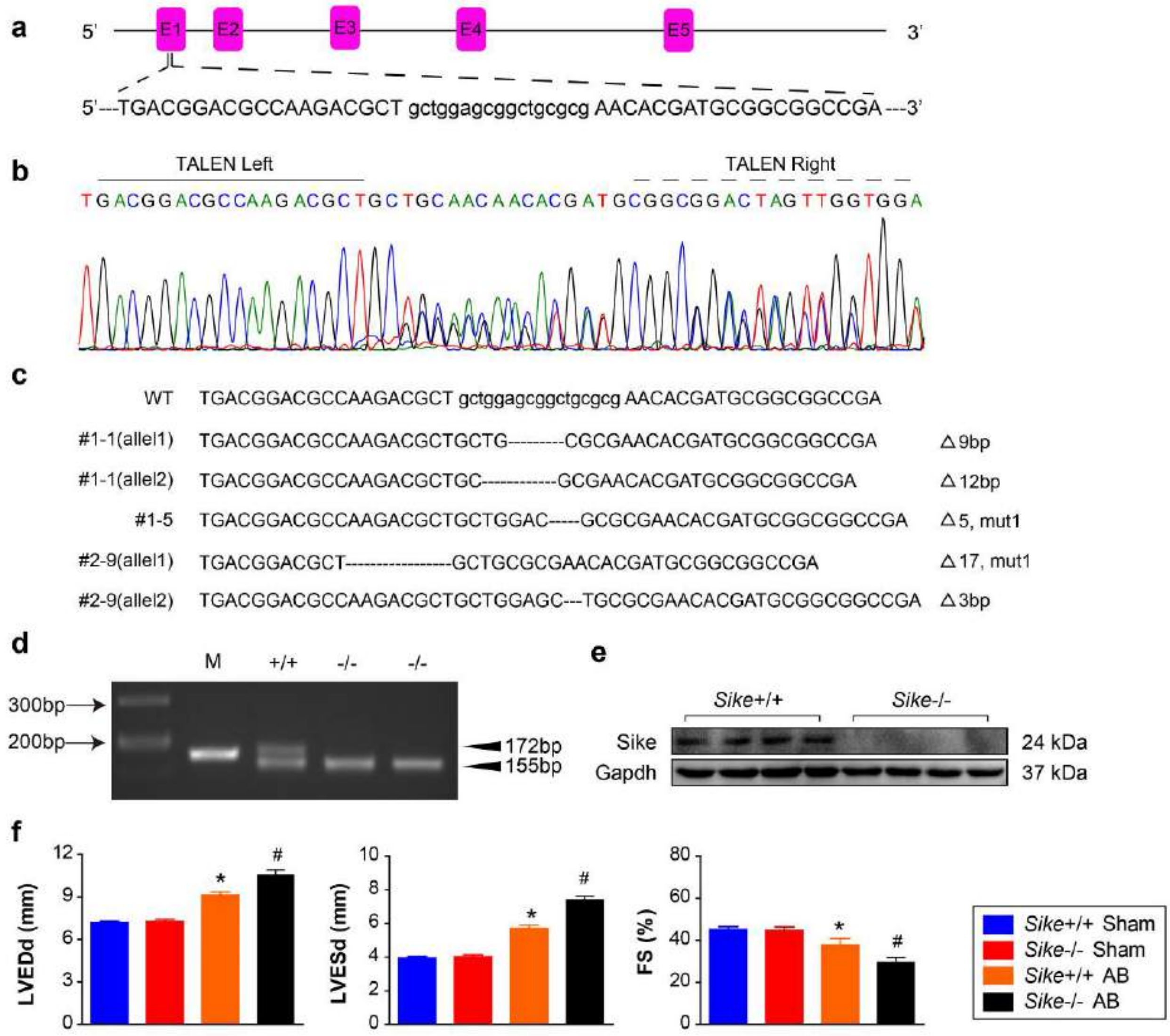

Supplementary Figure. 10 Sike deficiency exacerbates cardiac hypertrophy in rats after AB surgery. (a-c) The schematic diagram for the TALEN-mediated Sike knockout in SD rats displays *Sike* genomic locus and TALEN target site with the target DNA sequence **(a)**, the DNA-binding sequences and the spacer region for *Sike*-TALEN **(b)**, and the DNA sequences of the *Sike* locus from the F0 founder rats; '-' denotes deleted nucleotides (**c**). **(d-e)** Identification of Sike knockout rats via PCR **(d)**, in which a 172-bp band indicated the WT allele and a 155-bp band indicated the mutated *Sike* allele, and immunoblotting **(e)**, in which the loss of the Sike protein was confirmed in the hearts of *Sike*$^{-/-}$ rats. **(f)** Comparison of the echocardiographic parameters in the indicated groups, *n*=9-10 rats/group. Data are presented as the mean±s.d.. Statistical analysis was carried out by one-way ANOVA.

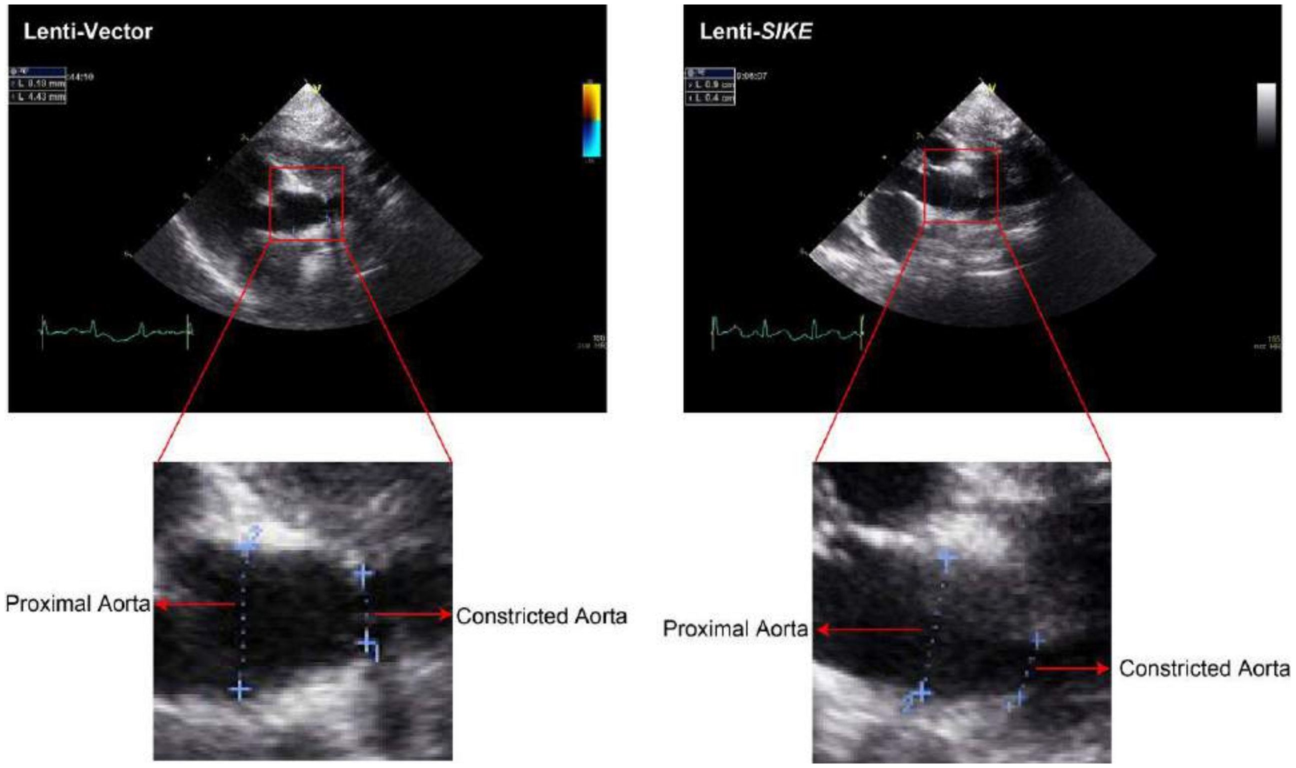

Supplementary Figure. 11 Measurements of aortic diameters in cynomolgus monkeys. Echocardiography were performed in cynomolgus monkeys from the indicated groups after AB surgery. The ascending aorta was zoomed in and the diameter of the constricted aorta and proximal aorta were measured at end-systole.

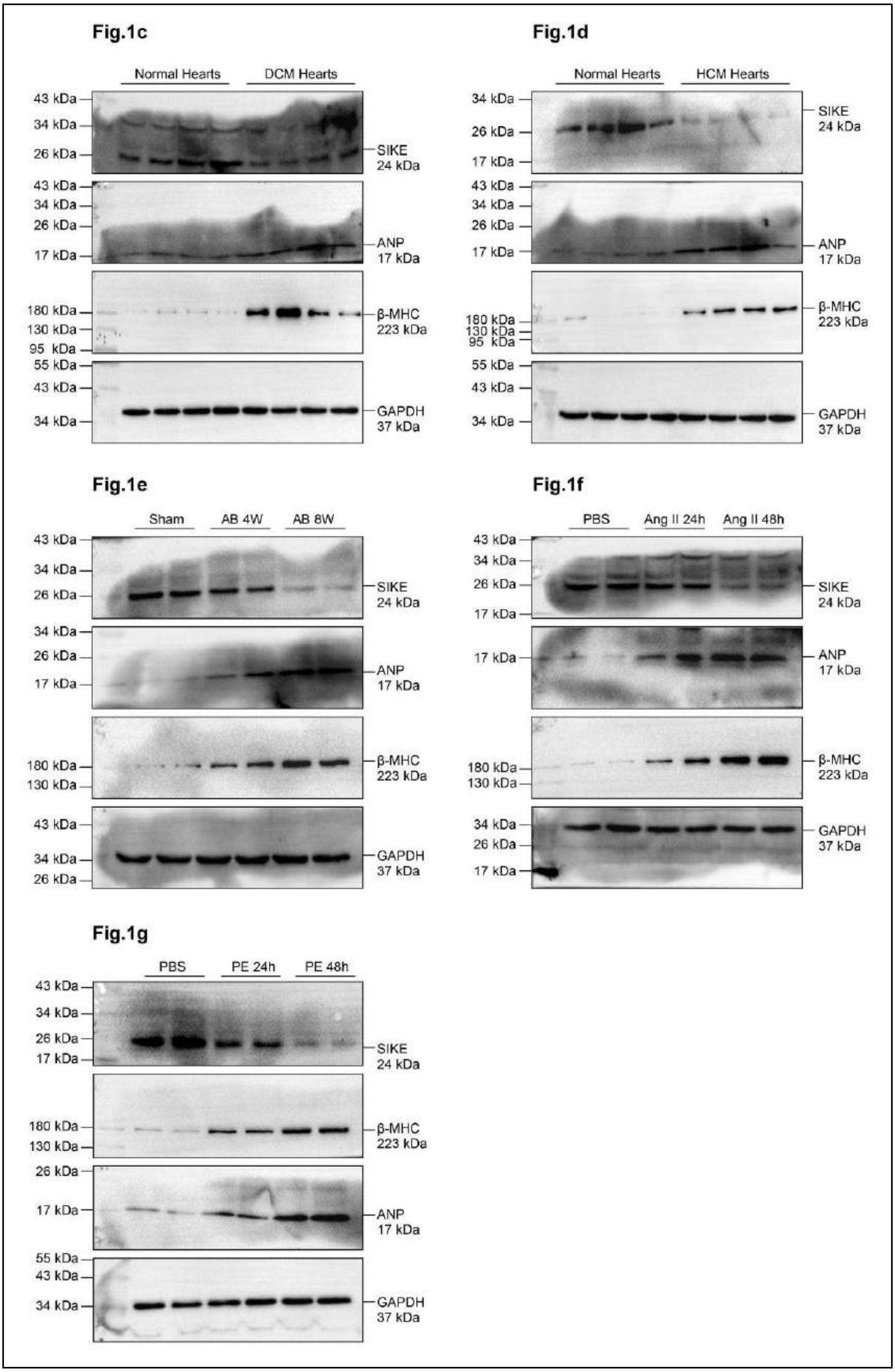

Supplementary Figure. 12 Full gel scans relating to indicated figures.

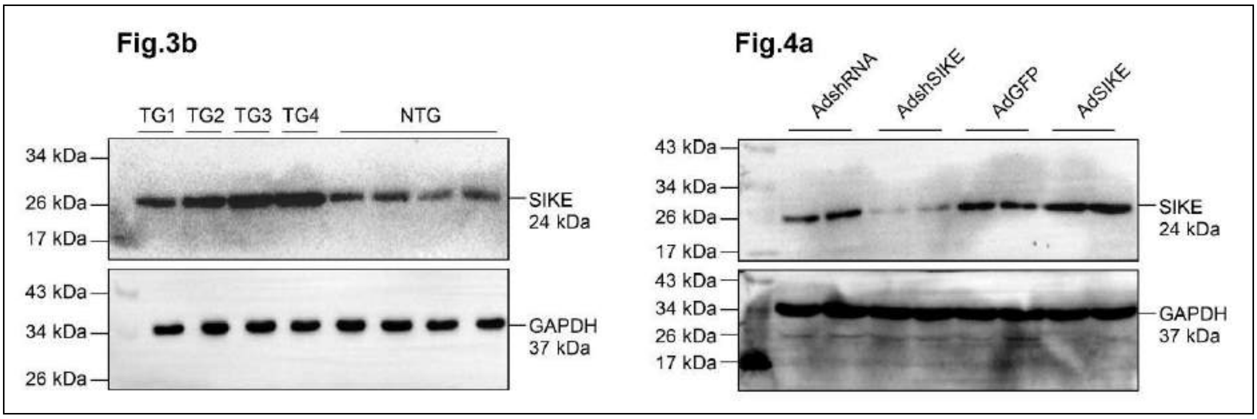

Supplementary Figure. 12 Full gel scans relating to indicated figures (continued).

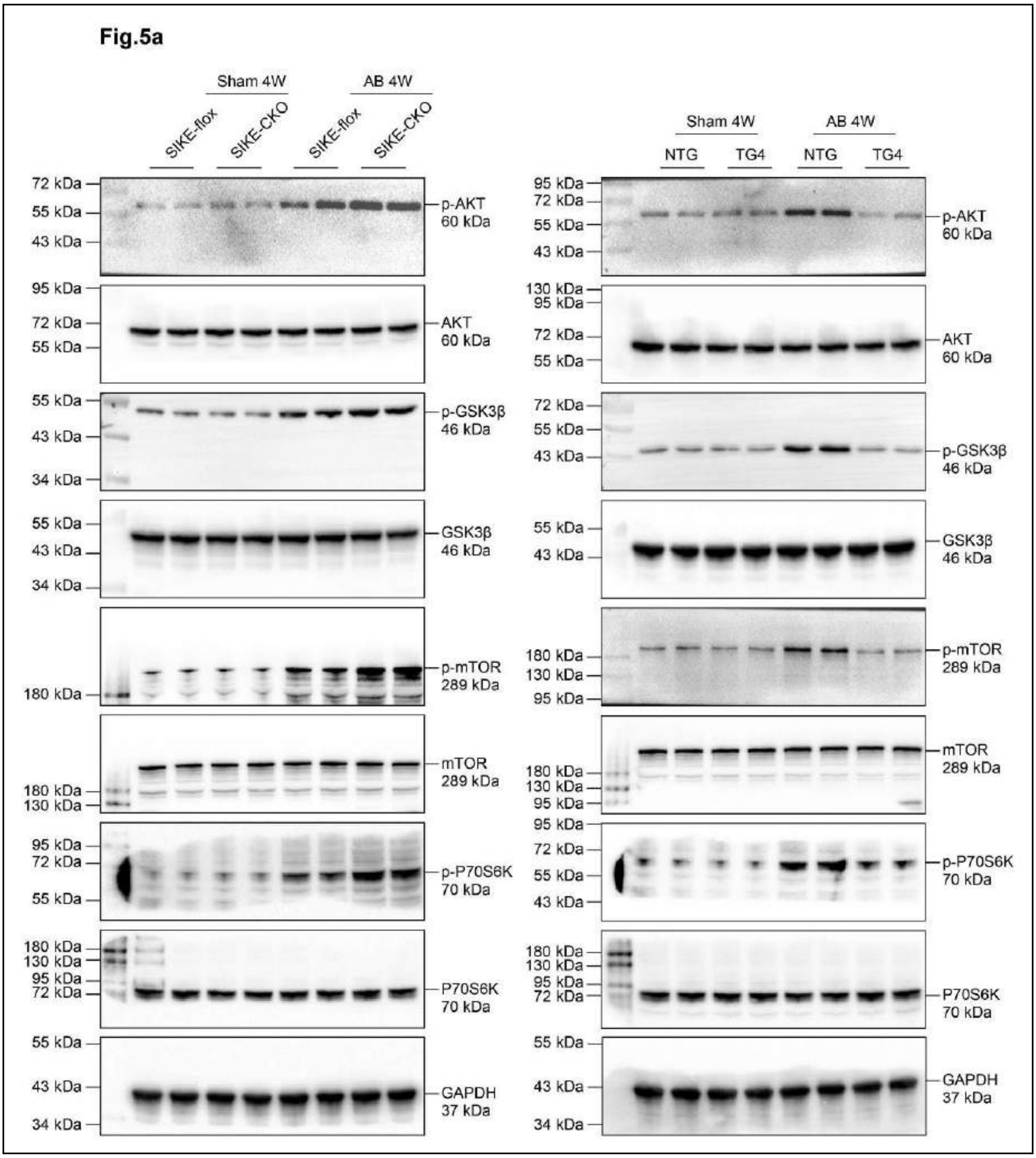

Supplementary Figure. 12 Full gel scans relating to indicated figures (continued).

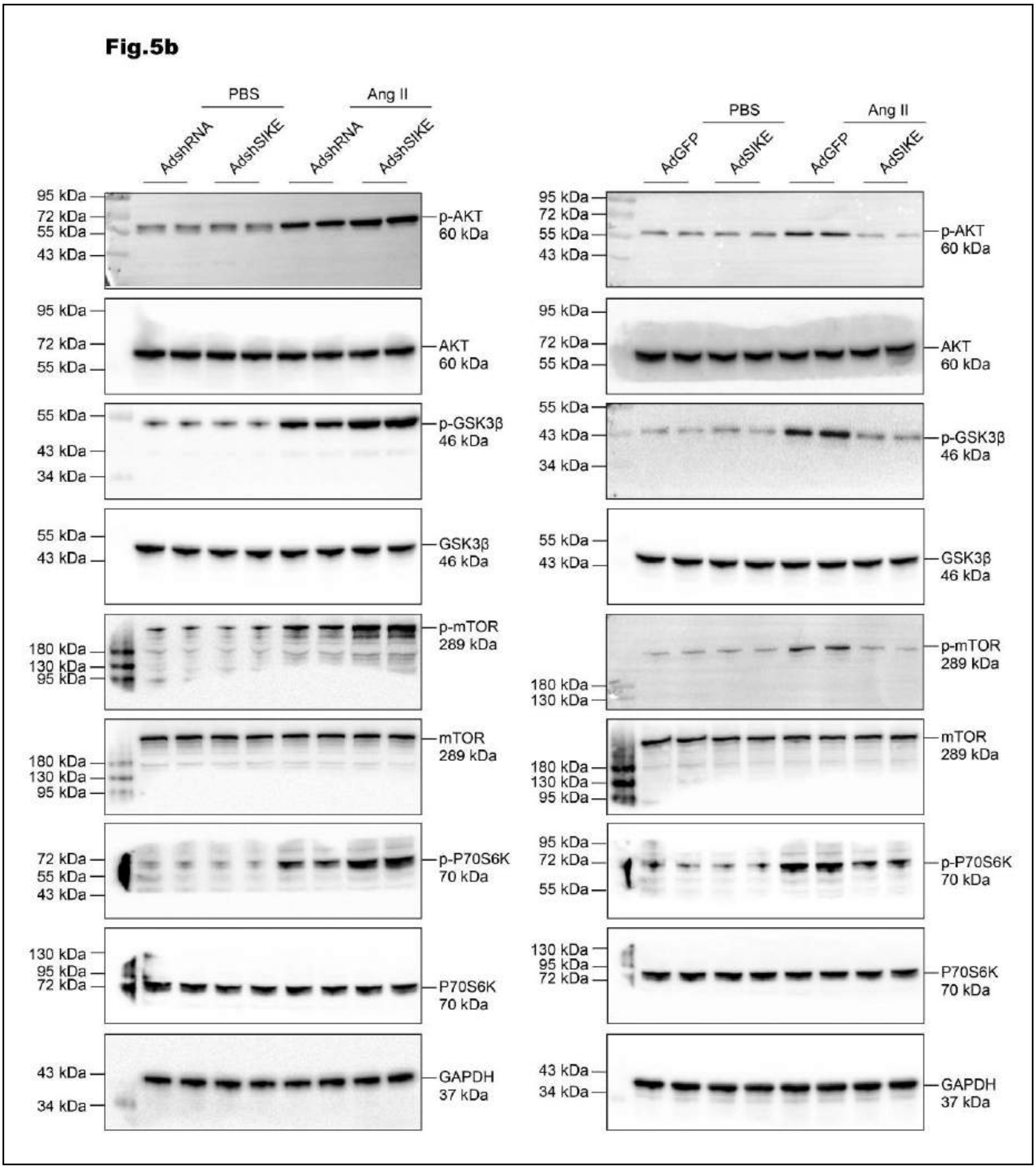

Supplementary Figure. 12 Full gel scans relating to indicated figures (continued).

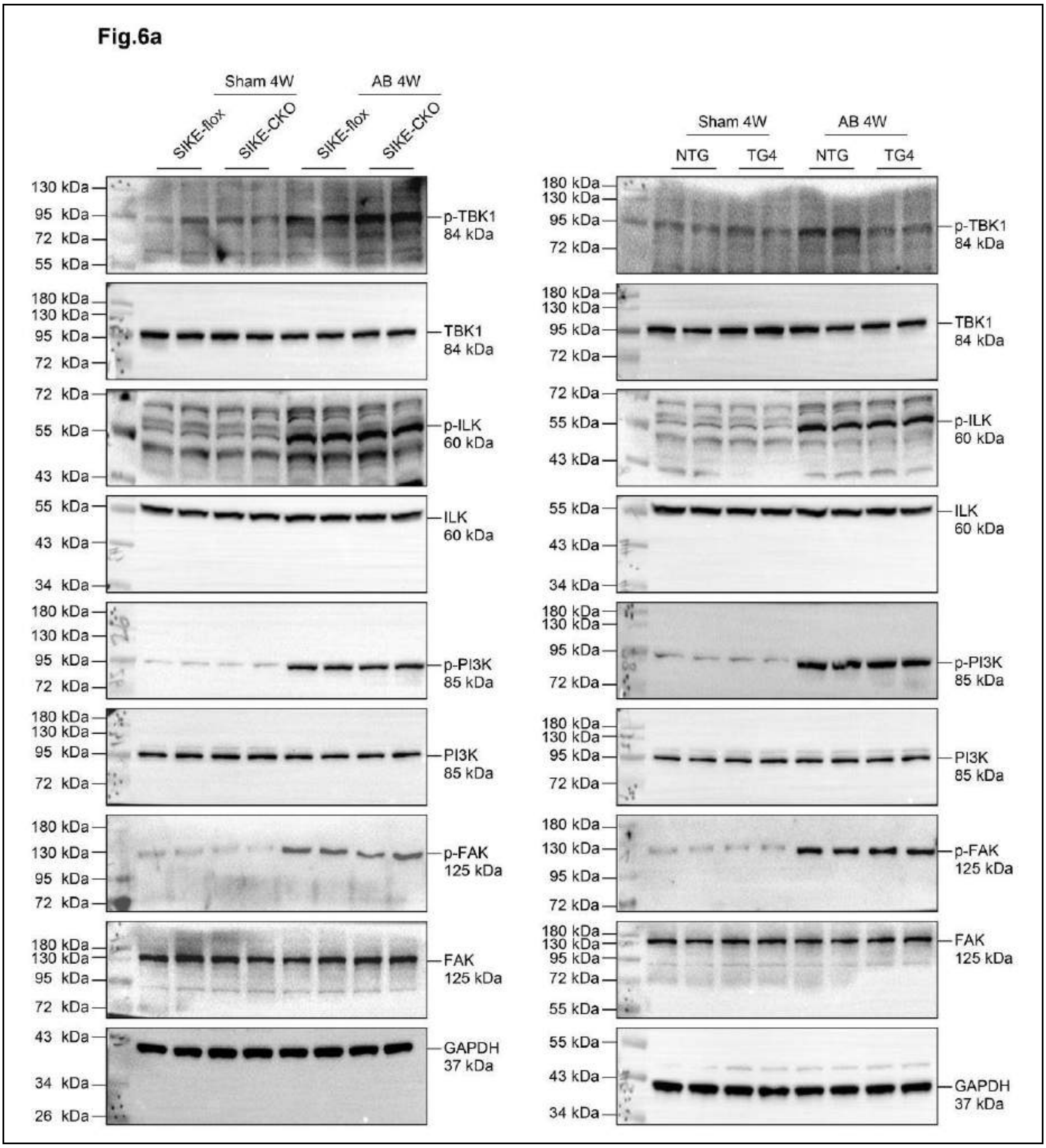

Supplementary Figure. 12 Full gel scans relating to indicated figures (continued).

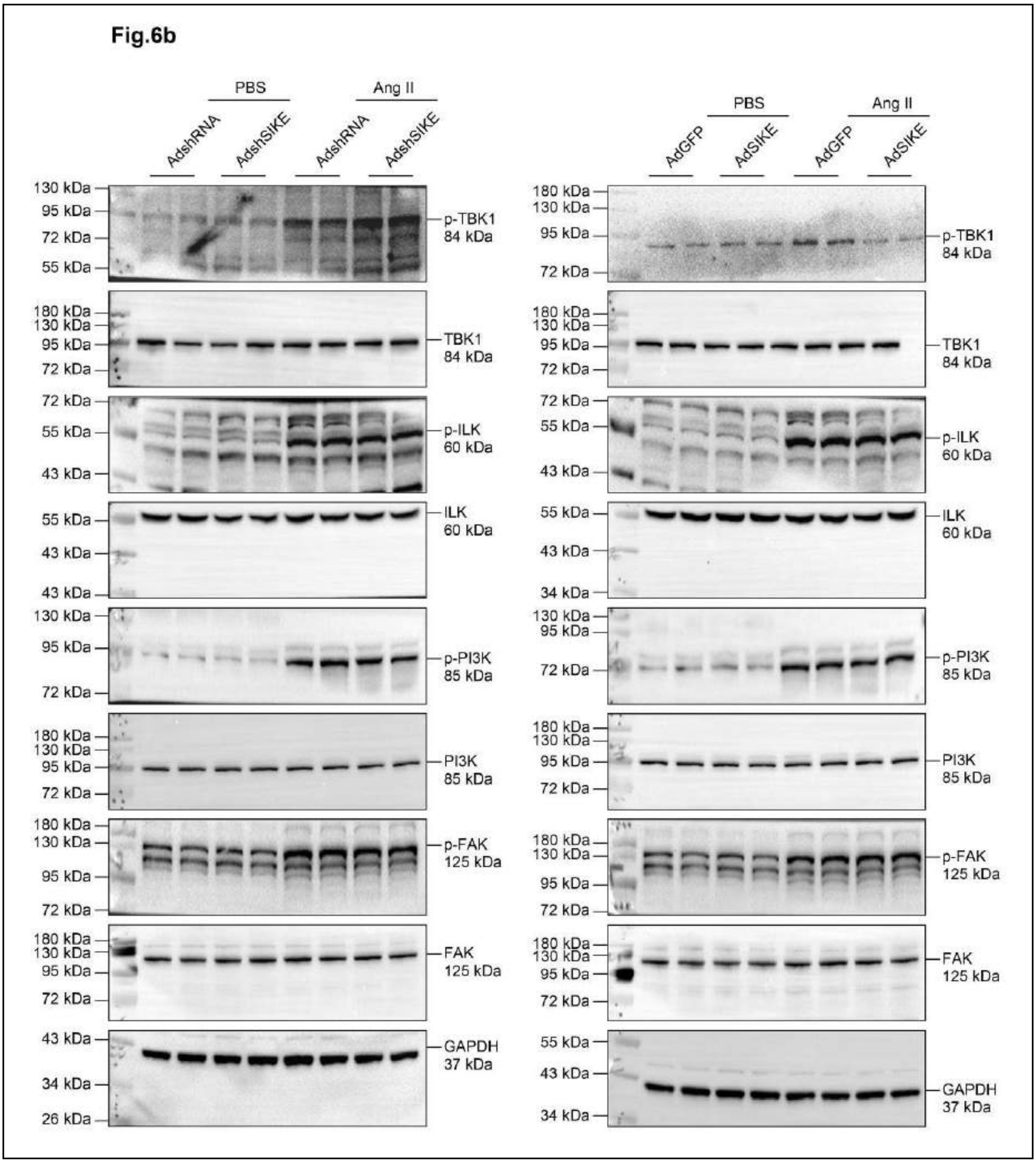

Supplementary Figure. 12 Full gel scans relating to indicated figures (continued).

Fig.7a

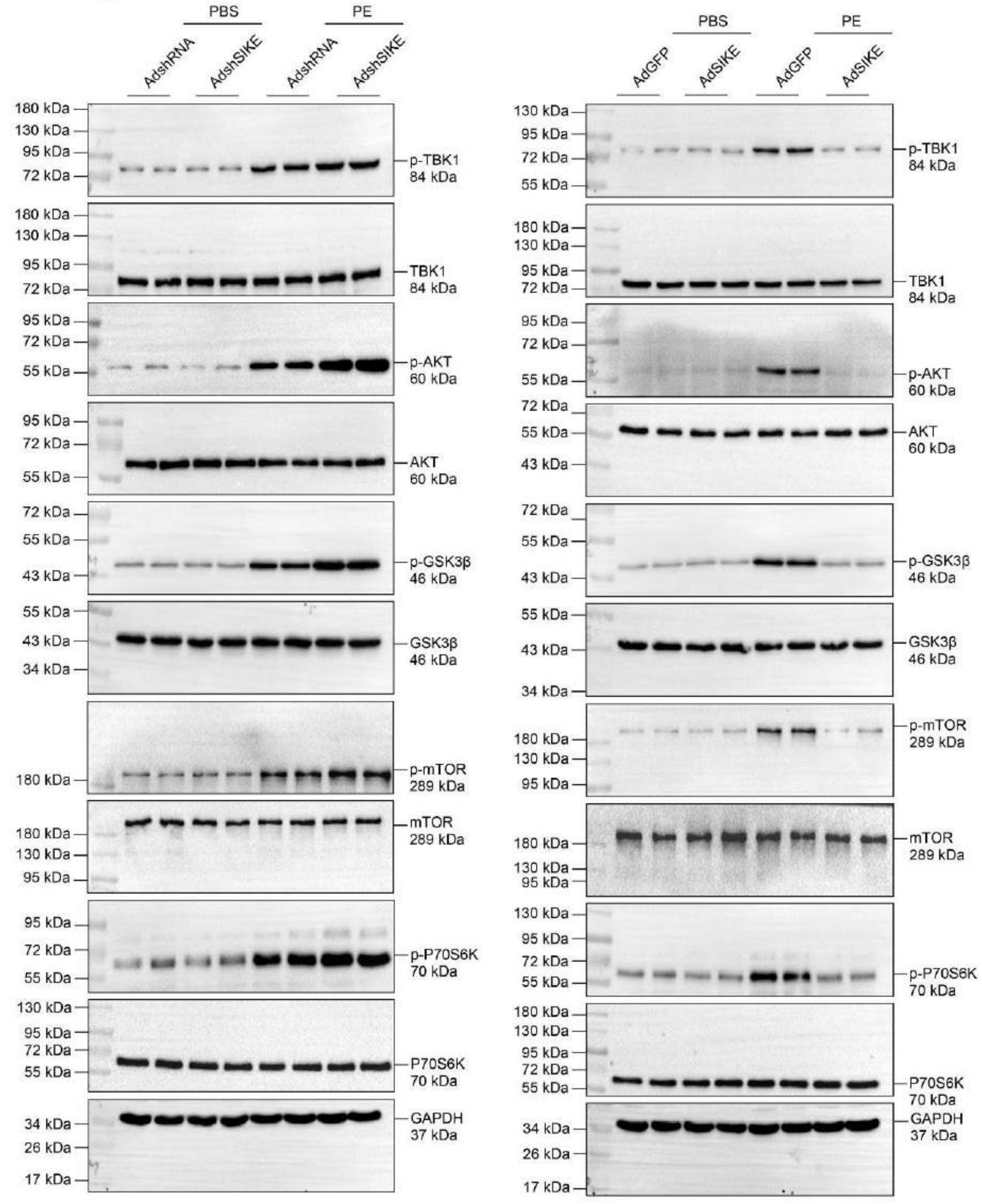

Supplementary Figure. 12 Full gel scans relating to indicated figures (continued).

Fig.7b

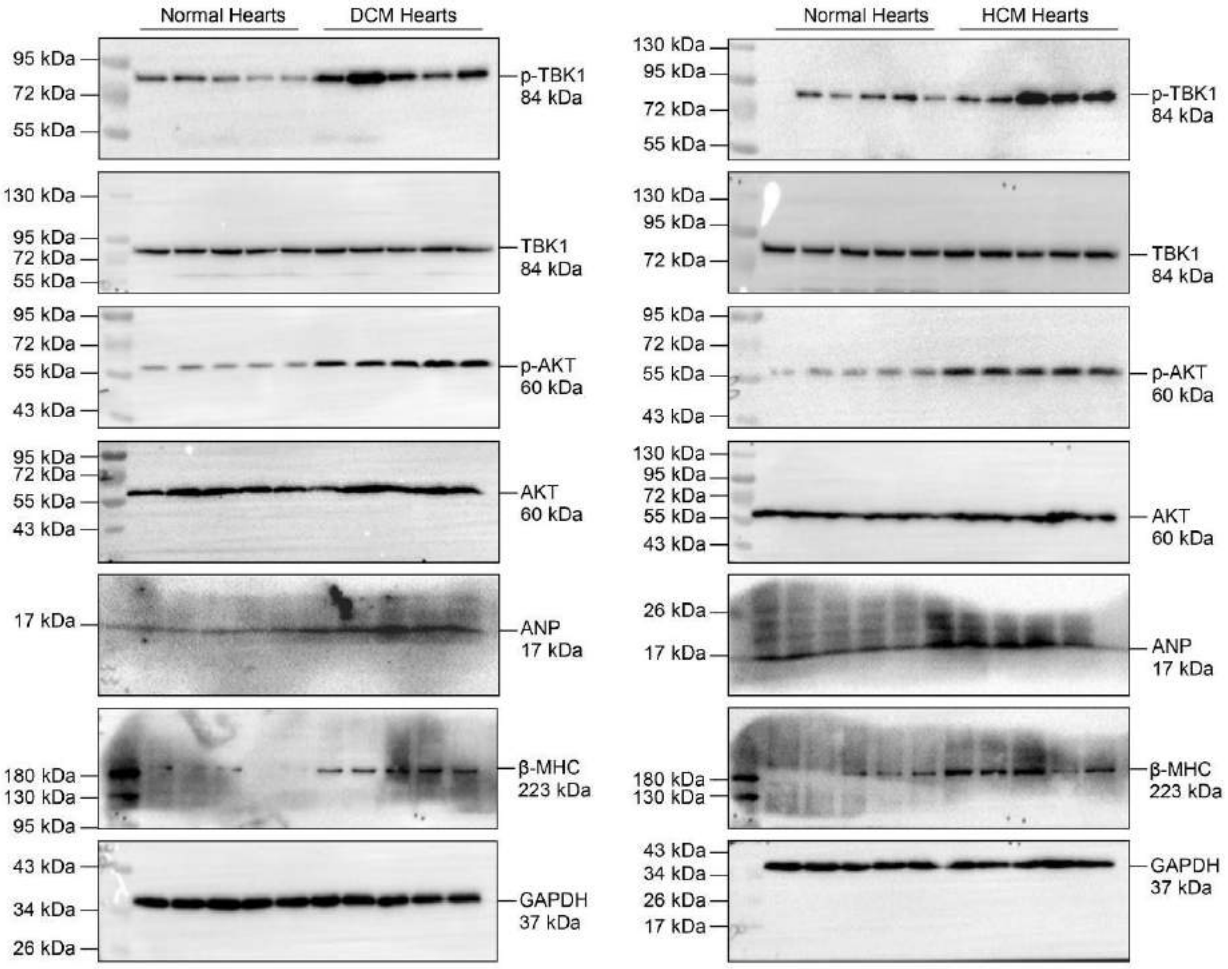

Supplementary Figure. 12 Full gel scans relating to indicated figures (continued).

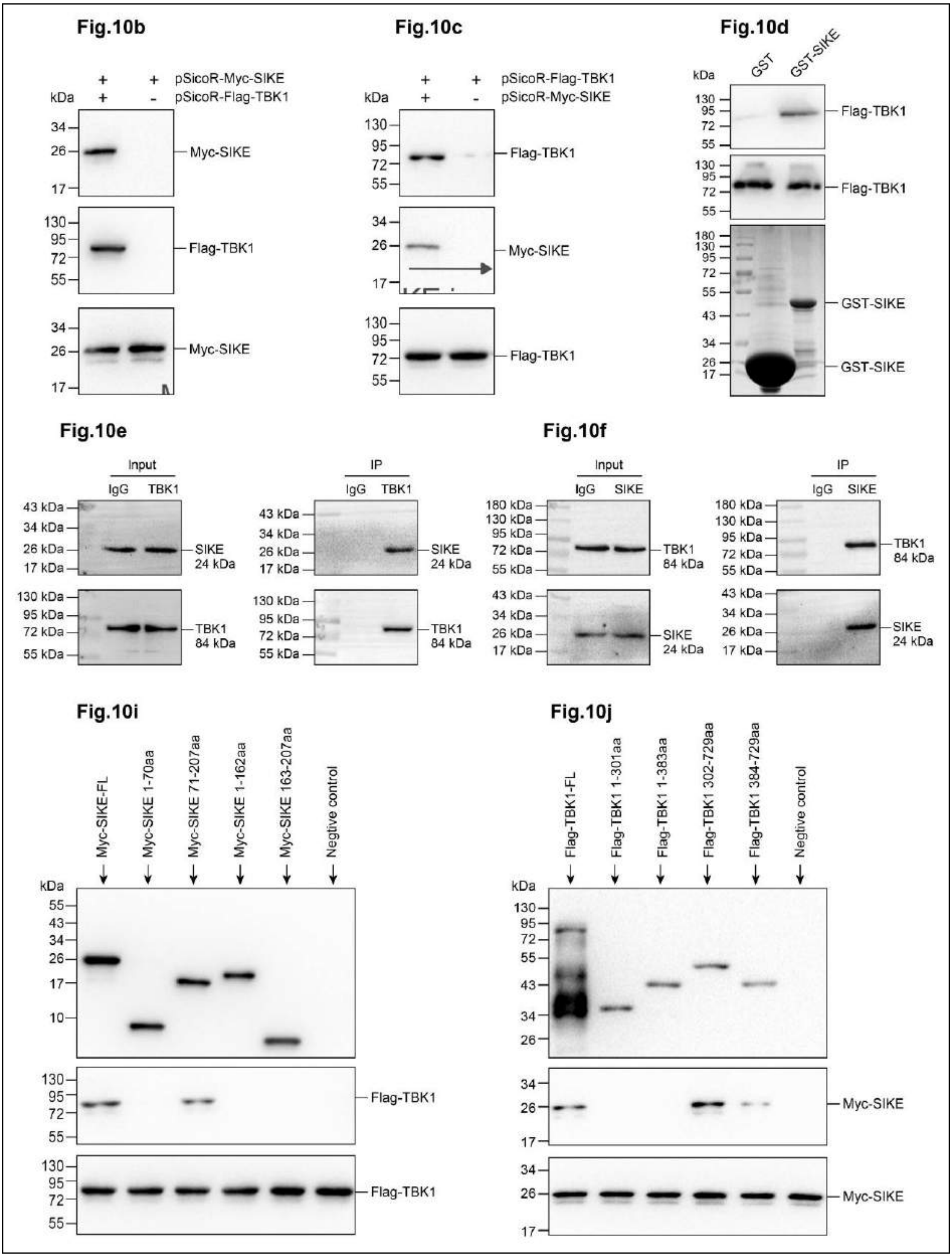

Supplementary Figure. 12 Full gel scans relating to indicated figures (continued).

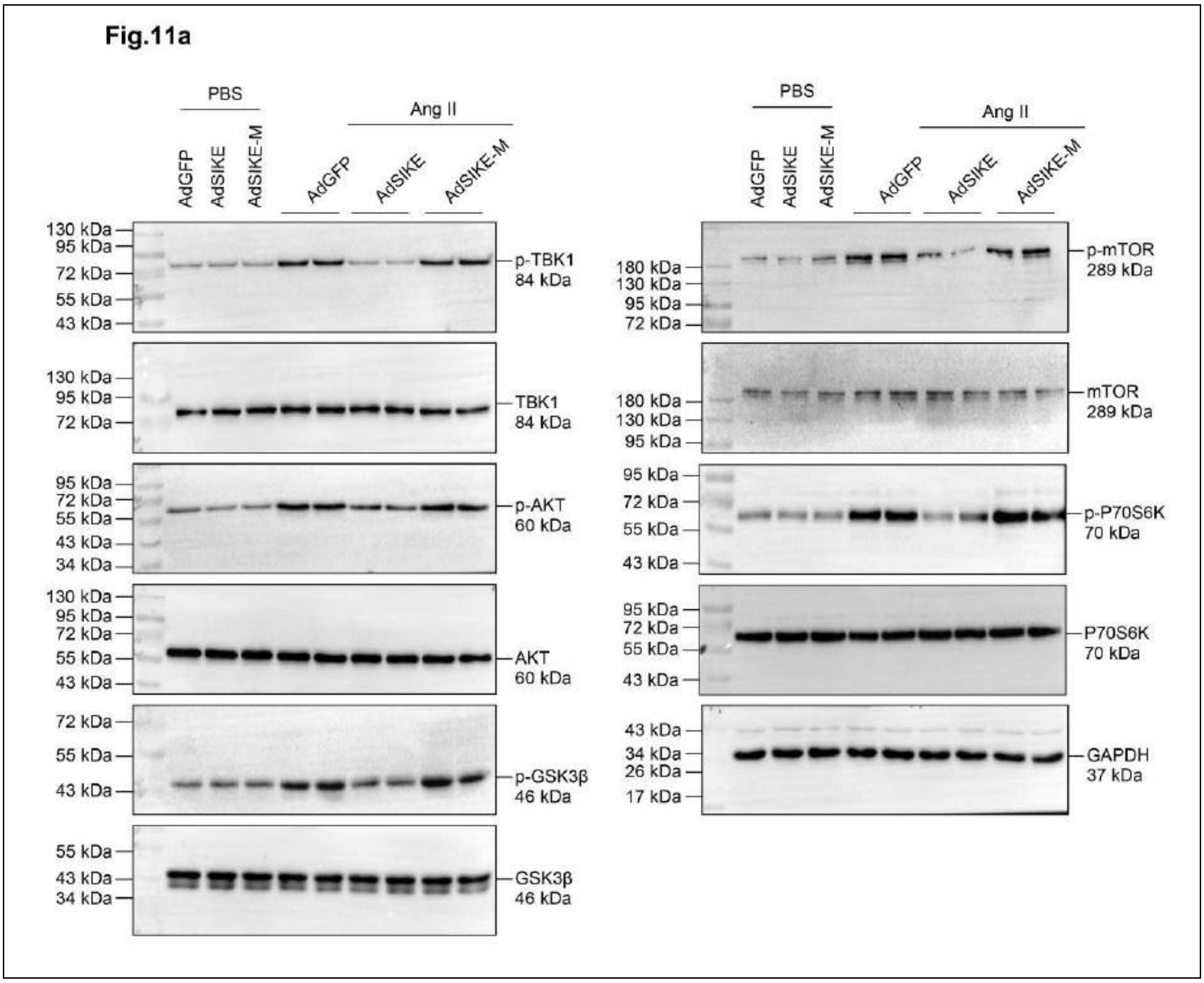

Supplementary Figure. 12 Full gel scans relating to indicated figures (continued).

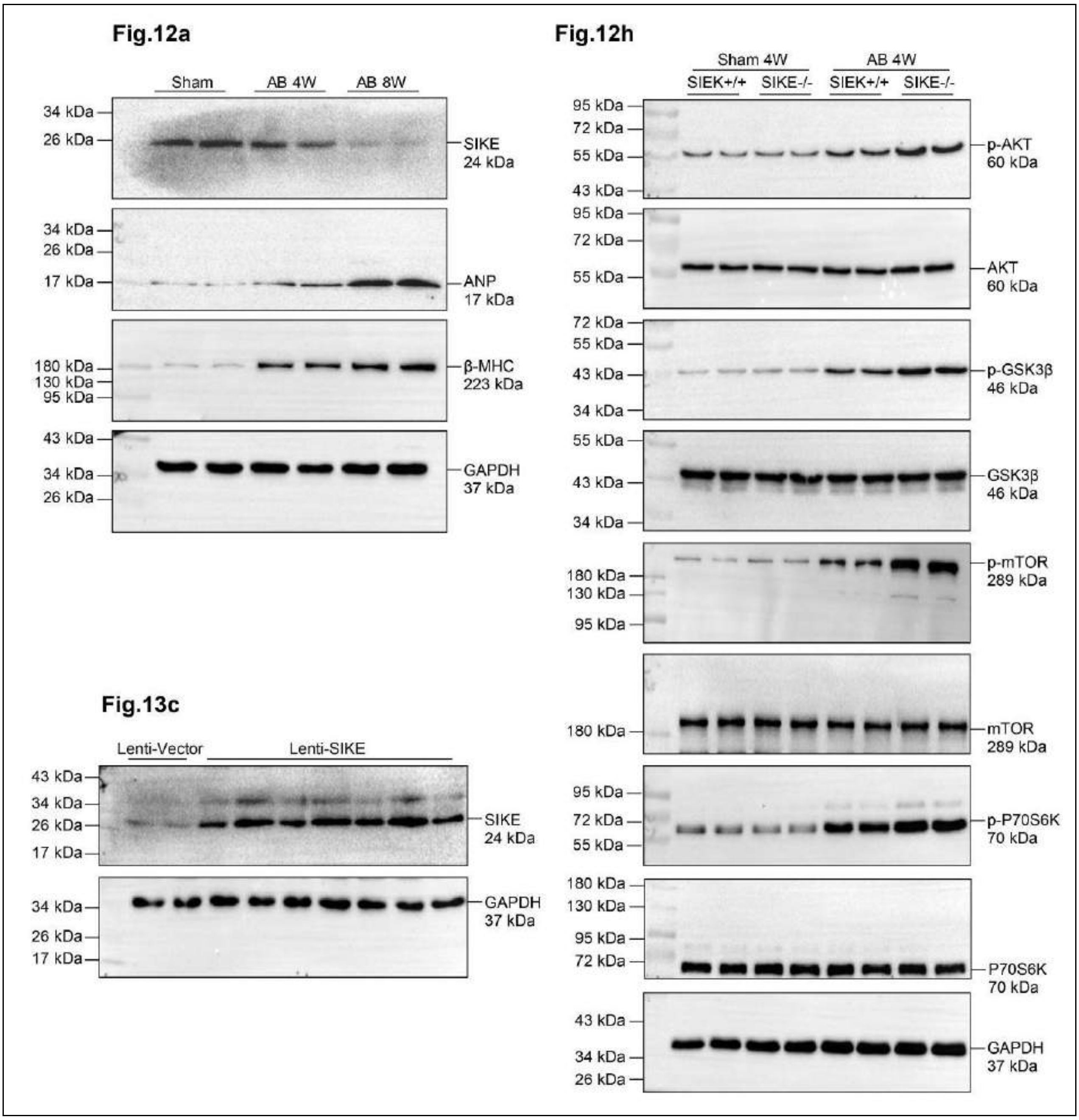

Supplementary Figure. 12 Full gel scans relating to indicated figures (continued).

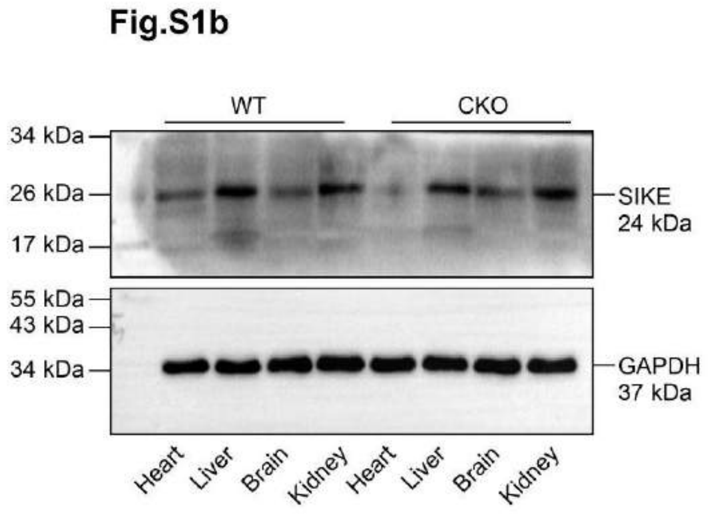

Supplementary Figure. 12 Full gel scans relating to indicated figures (continued).

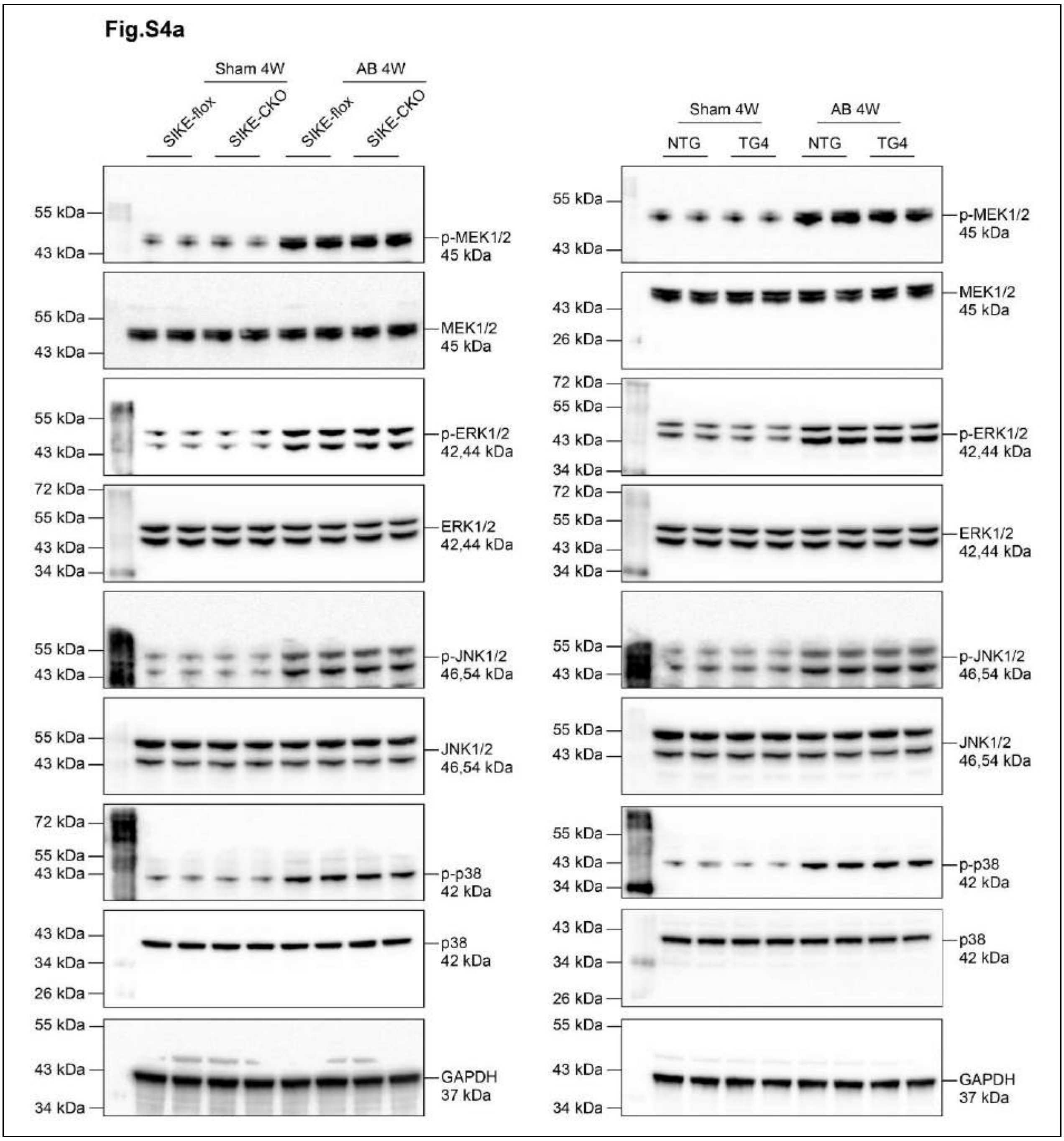

Supplementary Figure. 12 Full gel scans relating to indicated figures (continued).

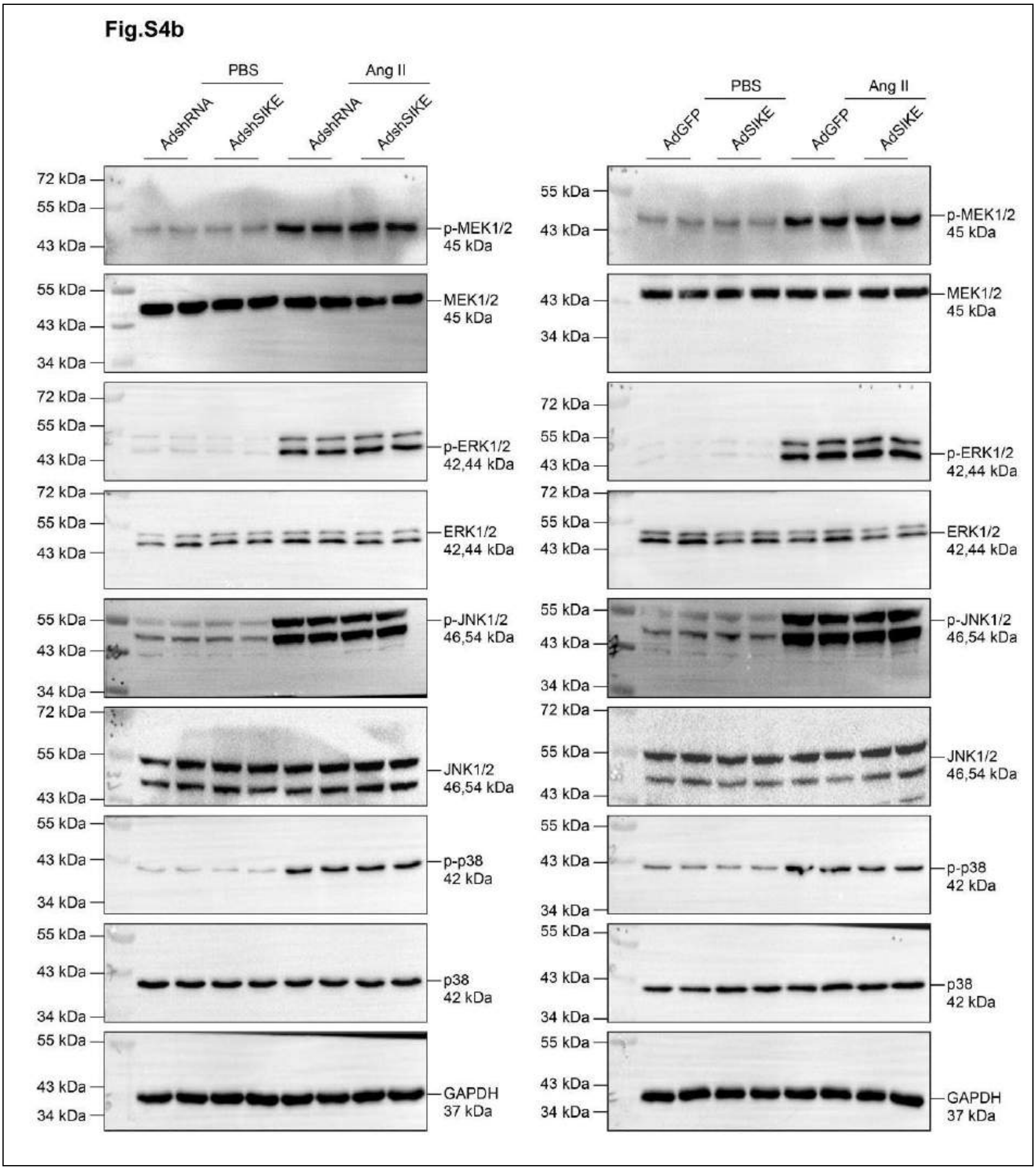

Supplementary Figure. 12 Full gel scans relating to indicated figures (continued).

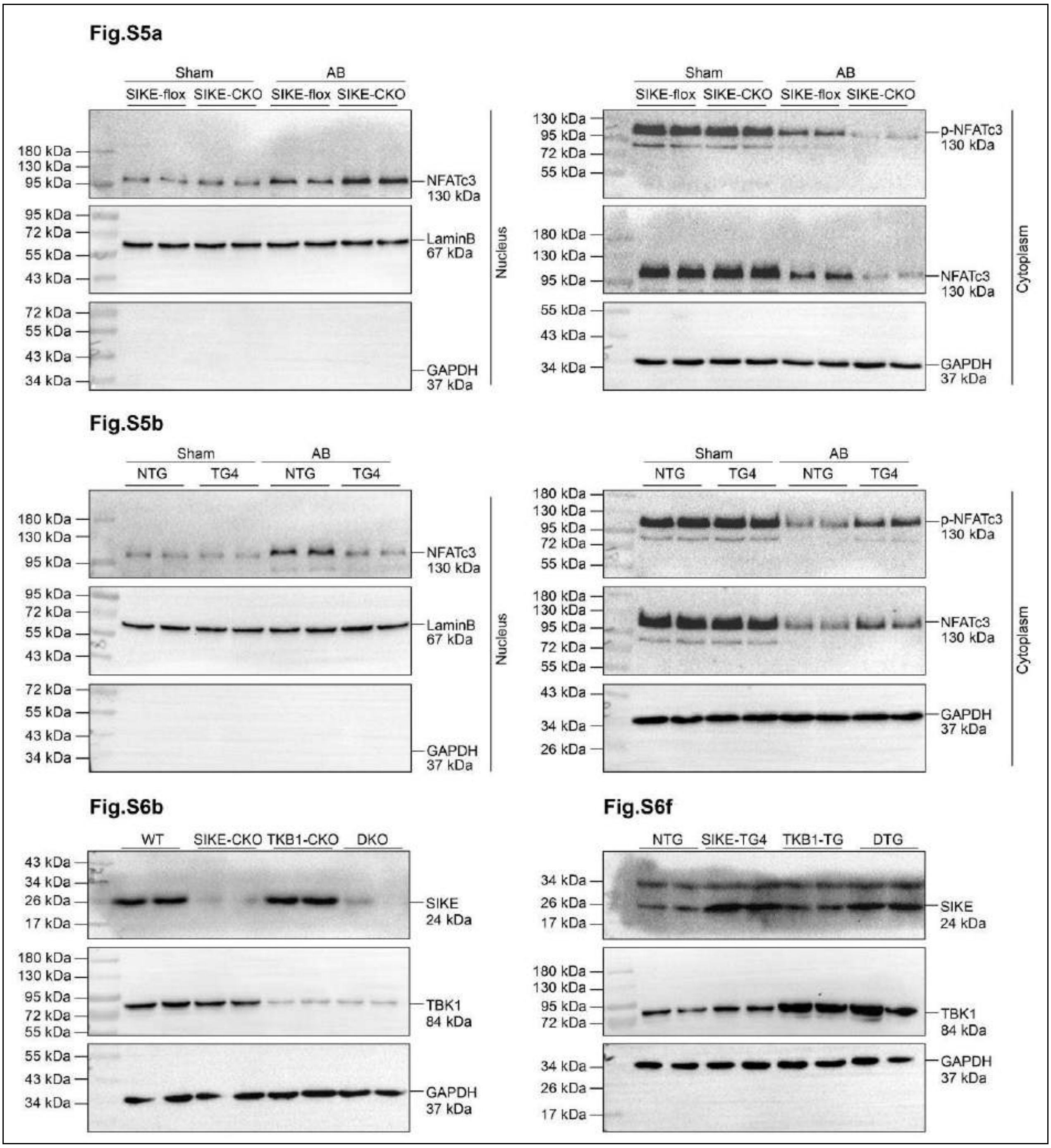

Supplementary Figure. 12 Full gel scans relating to indicated figures (continued).

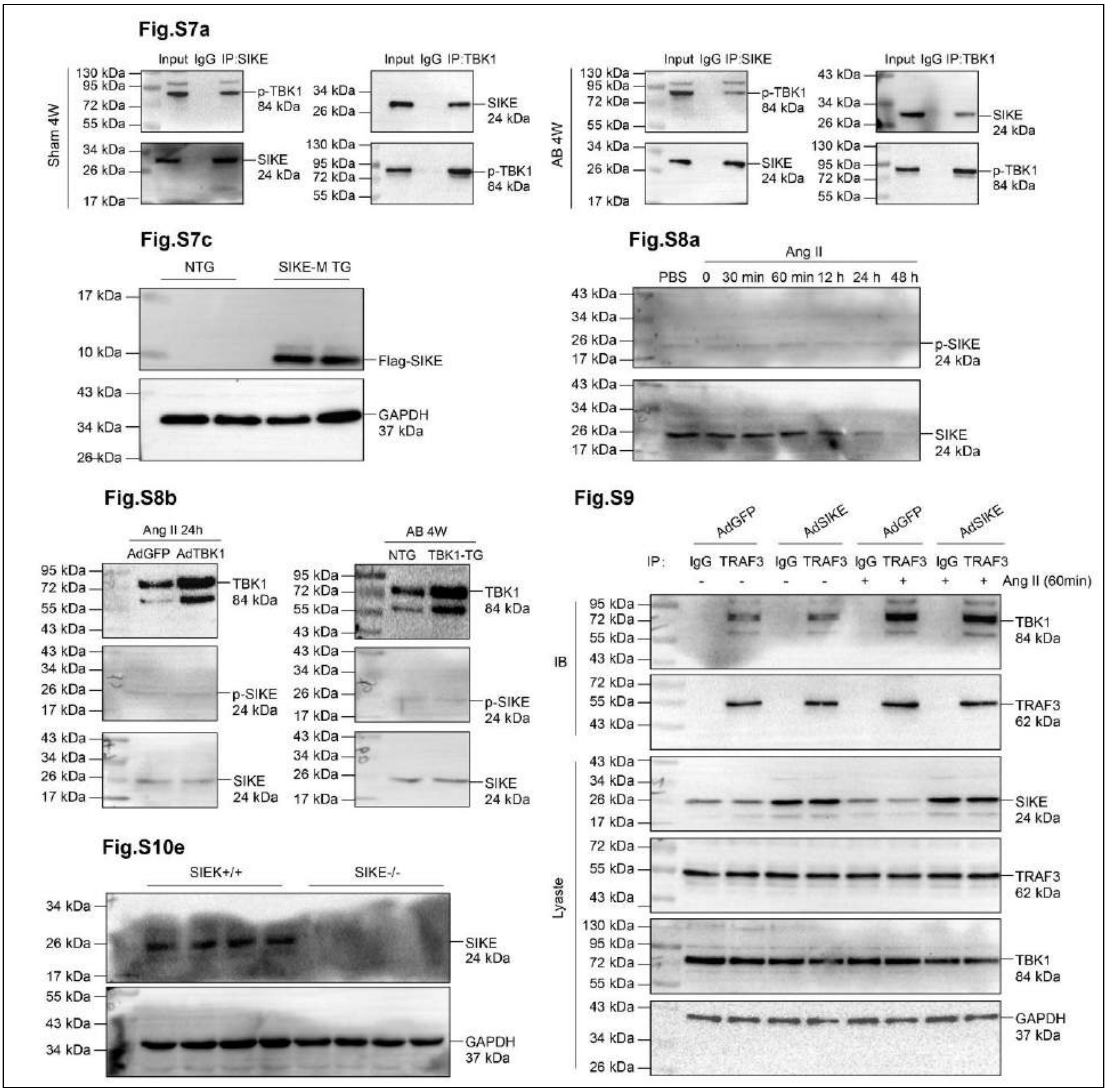

Supplementary Figure. 12 Full gel scans relating to indicated figures (continued).

Supplementary Table 1. The diameter and stenosis percentage of the aorta in each monkey

Group	Diameter (mm)		Stenosis Percentage (%)
	Proximal Aorta	Constricted Aorta	
Lenti-Vector 1	9.0	4.0	80.2
Lenti-Vector 2	8.5	3.4	84.0
Lenti-Vector 3	7.1	3.5	75.7
Lenti-Vector 4	9.1	3.6	84.3
Lenti-Vector 5	8.0	4.0	75.0
Lenti-Vector 6	8.1	4.2	73.0
Lenti-Vector 7	8.0	4.0	75.0
Lenti-Vector 8	9.2	4.4	77.1
Lenti-Vector 9	8.0	4.0	75.0
Lenti-Vector 10	8.4	3.3	84.6
Lenti-*SIKE* 1	9.0	4.0	80.2
Lenti-*SIKE* 2	8.3	3.9	77.9
Lenti-*SIKE* 3	8.0	4.2	72.4
Lenti-*SIKE* 4	8.3	4.1	75.6
Lenti-*SIKE* 5	7.8	4.0	73.7
Lenti-*SIKE* 6	10.0	3.8	85.6
Lenti-*SIKE* 7	6.3	3.1	75.8
Lenti-*SIKE* 8	7.2	3.1	81.5
Lenti-*SIKE* 9	8.3	3.3	84.2
Lenti-*SIKE* 10	10.4	4.2	83.7

Supplementary Table 2. The primers used to genotype the conditional cardiac-specific *Sike* knockout mice

**Primer name**	**Sequence (5' to 3')**
Sike-F1	TTGCGGAACCCTTCGAAGTTCC
Sike-R1	GATAGAAGATGAAACGTGGTGC
Sike-F2	ATTTAGATCAGTGCTTCAAGGGTC
Sike-R2	GGCTCCATCAGACAACTACCATC
Sike-F3	GAGGGAGGAAAGGAGAGGTTCC
Sike-R3	CGCAGAGGTGGACTCTTGGTTA

Sike-F1+ *Sike*-R1 to detect the gene targeting; product: 1690bp

Sike-F2+*Sike*-R2 to detect the wild type (WT) allele or FloxNeo allele; product: the WT band was 338bp, and the floxNeo allele band was 2kb

Sike-F3+*Sike*-R3 to detect the existence of left Loxp site; product: ΔLoxP→255bp; LoxP insert→290bp

Supplementary Table 3. The primers used to genotype the conditional cardiac-specific *Tbk1* knockout mice

Primer name	Sequence (5' to 3')
Tbk1-F1	TTGCGGAACCCTTCGAAGTTCC
Tbk1-R1	CATGCAGACTTGACAGCCATTG
Tbk1-F2	TGGTGGCTTTAGGAAGGATGTA
Tbk1-R2	CAGCCTTCTGGTGCAGTCTTAAA
Tbk1-F3	CACAACATAATTCCAGCTCCCG
Tbk1-R3	TCCATCATTCTTGTTCCGCTCT

Tbk1-F1+*Tbk1*-R1 to detect the gene targeting; product: 1240bp

Tbk1-F2+*Tbk1*-R2 to detect the WT allele or FloxNeo allele; product: the WT band was 242bp, and the floxNeo allele band was 1.87kb.

Tbk1-F3+*Tbk1*-R3 to detect the existence of left Loxp site; product: ΔLoxP→242bp; LoxP insert→280 bp

Supplementary Table 4. Antibodies used in this study

Antibody	Manufacturer	Catalogue number	Sources of species	Application and working dilution
SIKE	Abcam	ab183509	rabbit	IHC-P (1:50)<br>WB (1:10000)<br>IP (1:30)
p-AKT	CST	4060	rabbit	WB (1:2000)
AKT	CST	4691	rabbit	WB (1:1000)
p-mTOR	CST	2971	rabbit	WB (1:1000)
mTOR	CST	2983	rabbit	WB (1:1000)
p-GSK3β	CST	9322	rabbit	WB (1:1000)
GSK3β	CST	9315	rabbit	WB (1:1000)
p-P70S6K	CST	9208	rabbit	WB (1:1000)
P70S6K	CST	2708	rabbit	WB (1:1000)
p-TBK1	CST	5483	rabbit	WB (1:1000)
TBK1	CST	3013	rabbit	WB (1:1000)<br>IP (1:100)
p-PI3K	CST	4228	rabbit	WB (1:1000)
PI3K	CST	4257	rabbit	WB (1:1000)
p-ILK	Abgent	AP3679a	rabbit	WB (1:500)
ILK	CST	ab76468	rabbit	WB (1:5000)
p-MEK1/2	CST	9154	rabbit	WB (1:1000)
MEK1/2	CST	9122	rabbit	WB (1:1000)
p- ERK1/2	CST	4370	rabbit	WB (1:2000)
ERK1/2	CST	4695	rabbit	WB (1:1000)
p-P38	CST	4511	rabbit	WB (1:1000)
P38	CST	9212	rabbit	WB (1:1000)
p-JNK	CST	4668	rabbit	WB (1:1000)
JNK	CST	9252	rabbit	WB (1:1000)
p-FAK	CST	3284	rabbit	WB (1:1000)
FAK	Bioworld	BS3583	rabbit	WB (1:1000)
TRAF3	CST	4729	rabbit	WB (1:1000)
β-MHC	SCBT	sc-53090	rabbit	WB (1:200)
ANP	SCBT	sc-20158	rabbit	WB (1:200)
p-NFATc3	GeneTex	GTX52339	rabbit	WB (1:500)
NFATc3	SCBT	sc-8405	mouse	WB (1:200)
Lamin B	SCBT	sc-6217	goat	WB (1:200)
Myc	Roche	11814150001	mouse	IP (1:100)
Flag	Sigma	F3165	mouse	IP (1:100)
GAPDH	Bioworld	MB001	mouse	WB (1:10000)

IHC-P: Immunohistochemitry (paraffin-embedded sections); WB: Western blot; IP: Immunoprecipitation.

Supplementary Table 5. The primers for Real-Time PCR

Gene	Forward Primer (5' to 3')	Reverse Primer (5' to 3')
SIKE-Human	GGCTTACATCCCTCATGCTGT	ATAGCTGGCACAGGCCATTT
GAPDH-Human	CATCACCATCTTCCAGGAGCGAGA	TGCAGGAGGCATTGCTGATGATCT
ANP-Monkey	TCCGATCGATCTGCCCTCTT	GAAGCAACTGGATCTCCGCA
β-MHC -Monkey	GGAGCTGATGCGCCTATTGA	TGGAGCGCAAGTTGGTCATC
GAPDH-Monkey	CCCATGTTCGTCATGGGTGT	TCTTCTGGGTGGCAGTGATG
Sike-Mouse	ATGGTCGCTAAGAAAGCCGT	CTGAACAGCTCTCCGCATCA
Anp-Mouse	ACCTGCTAGACCACCTGGAG	CCTTGGCTGTTATCTTCGGTACCGG
Bnp-Mouse	GAGGTCACTCCTATCCTCTGG	GCCATTTCCTCCGACTTTTCTC
β-Mhc-Mouse	CCGAGTCCCAGGTCAACAA	CTTCACGGGCACCCTTGGA
Ctgf-Mouse	TGACCCCTGCGACCCACA	TACACCGACCCACCGAAGACACAG
Collagen I-Mouse	AGGCTTCAGTGGTTTGGATG	CACCAACAGCACCATCGTTA
Collagen III-Mouse	CCCAACCCAGAGATCCCATT	GAAGCACAGGAGCAGGTGTAGA
Gapdh-Mouse	ACTTGAAGGGTGGAGCCAAA	GACTGTGGTCATGAGCCCTT

Supplementary Table 6. The primers for the creation of SIKE/TBK1 construct

Primer name	Primer (5' to 3')
h-*SIKE*-F-BamH I	CGCGGATCCATGAGCTGCACCATCGAGAAGA
h-*SIKE*-R70- Xho I	CCGCTCGAGTTAGTGAGGTTTGTATTTGGACA
h-*SIKE*-F71-BamH I	CGCGGATCCATGATTCTGCTGTCCCAAGAGAA
h-*SIKE*-R- Xho I	CCGCTCGAGTTATTTGATGGCTTGGGAAG
h-*SIKE*-R162- Xho I	CCGCTCGAGTTACTGGTCATCATCCACCTGAA
h-*SIKE*-F163-BamH I	CGCGGATCCATGTTTTGTAAGATTCAGGAAAAAT
h-*TBK1*-F-BamH I	CGCGGATCCATGCAGAGCACTTCTAATCA
h-*TBK1*-R301-Xho I	CCGCTCGAGCTATTCTGCAAAAAACTGGTCAA
h-*TBK1*-F302-BamH I	CGCGGATCCATGACTAGTGATATACTTCACCGA
h-*TBK1*-R-Xho I	CCGCTCGAGCTAAAGACAGTCAACGTTGC
h-*TBK1*-R383-Xho I	CCGCTCGAGCTAGCTTACTACAAATATAGGGT
h-*TBK1*-F384-BamH I	CGCGGATCCATGCGGGAACCTCTGAATACCATAGG
